# Fruit Morphology and Anatomy of the Spondioid Anacardiaceae

**DOI:** 10.1007/s12229-018-9201-1

**Published:** 2018-08-03

**Authors:** Fabiany Herrera, John D. Mitchell, Susan K. Pell, Margaret E. Collinson, Douglas C. Daly, Steven R. Manchester

**Affiliations:** 1 Chicago Botanic Garden, Glencoe, IL USA; 2 Institute of Systematic Botany, The New York Botanical Garden, Bronx, NY USA; 3 United States Botanical Garden, Washington, DC USA; 4 Department of Earth Sciences, Royal Holloway University of London, Egham, Surrey UK; 5 Florida Museum of Natural History, University of Florida, Gainesville, FL USA

**Keywords:** Anacardiaceae, Fruit, Endocarp, Fossils, Phylogeny, Spondioideae, Systematics

## Abstract

The Spondioideae subfamily of the Anacardiaceae is widely distributed today in tropical regions. Recent molecular phylogenetic investigations indicate that the Spondioideae are not monophyletic, but rather comprise at least two separate clades that are difficult to distinguish using vegetative and floral characters. Nevertheless, the syndrome of fruit characters traditionally used in identifying the subfamily is useful in discriminating genera of these clades and for identification of both modern and fossil anacardiaceous fruits. Here we document the morphology and anatomy of endocarps for representatives of all extant genera traditionally treated as Spondioideae, plus two genera that have been placed close to them in molecular investigations, *Buchanania* and *Campnosperma*. All genera are characterized by drupe-like fruits with sclerified stones that vary from uni- to multilocular depending on the genus. Germination modes vary throughout the Spondioideae. Some have characteristic plug-like opercula; others have recessed bilabiate germination valves, and still others open by apical flaps or simple slits. Although most currently recognized genera appear to be monophyletic, fruit morphology indicates that current circumscriptions of *Cyrtocarpa*, *Poupartia* and *Tapirira* are in need of revision.

## Introduction

The Anacardiaceae are a widely distributed family of the order Sapindales (Muellner-Riehl et al., 2016), particularly well represented in tropical regions of the world today. The family includes economically important members such as cashew, pistachio, and mango, various timber woods, and some dermatologically toxic plants like poison ivy (Pell et al., 2011). Fruits of the family vary from multilocular to unilocular drupes to samaras with conspicuous wings (Wannan & Quinn, 1990; Pell et al., 2011). Endocarp morphology is informative for the recognition of genera in the Anacardiaceae, so the recovery of fossil endocarps can provide insight into the evolution and biogeographic history of major clades. However, the limited amount of detailed comparative information on fruit morphology of extant representatives has hampered the systematic evaluation of fossil representatives.

Recent classifications of Anacardiaceae recognize two subfamilies, Anacardioideae and Spondioideae (Mitchell et al., 2006; Pell, 2004; Pell et al., 2011). However, molecular investigations, based on three DNA sequence regions [nuclear ribosomal external transcribed spacer (*ETS*), chloroplast *trnL* intron and *trnL-F* intergenic spacer (*trnL-F* region), and the chloroplast *rps16* intron)], encompassing 67 anacardiaceous genera (out of 82), as well as 16 genera of the related family Burseraceae, indicate that both subfamilies are polyphyletic (Weeks et al., 2014). Here we focus on the genera traditionally treated as Spondioideae. In the analyses of Weeks et al. (2014), the Spondioideae fall into two unrelated clades (named informally Spondioideae 1 and 2).

The genera of “Spondioideae 1” sensu Weeks et al. (2014), here called the *Sclerocarya* complex, constitute a clade that includes *Antrocaryon*, *Choerospondias*, *Cyrtocarpa*, *Harpephyllum*, *Lannea*, *Operculicarya*, *Pleiogynium*, *Poupartia*, *Poupartiopsis*, *Sclerocarya*, and *Tapirira*. Genera of “Spondioideae 2,” here called the *Spondias* complex, including *Allospondias*, *Dracontomelon*, *Pegia*, *Pseudospondias*, and *Spondias,* are depicted as early-divergent taxa within the familial phylogeny, being sister to all other Anacardiaceae except *Pentaspadon*. The precise relationships of the two complexes to each other remain poorly supported (pp < 0.7 for intervening nodes), but the monophyly of each is well supported (pp = 1; Weeks et al., 2014). Some genera that have been classified within Spondioideae have not yet been analyzed molecularly namely: *Haematostaphis*, *Haplospondias*, *Koordersiodendron*, and *Solenocarpus*.

Spondioideae are characterized by drupe-like fruits with stones that are somewhat woody and usually composed of irregularly oriented fibers and brachysclereids. The fruits have one to several locules, each with a single seed, with or without specialized structures for germination, such as stopper-like opercula or valves that hinge open. Diagnostic features for the distinction of genera include shape and symmetry of the fruit, number and configuration of locules and lacunae, the kind of germination apparatus (e.g., presence or absence of opercula), the arrangement of sclerenchyma within the fruit stone, and the presence/absence and distribution of surface apertures linked by channels to the lacunae (Mitchell et al., 2006).

In this treatment, we summarize fruit morphology and endocarp anatomy for all genera of Spondioideae following the classification of Pell et al. (2011). We provide new imagery and observations for all extant genera and review prior treatments for those genera studied previously. This information is intended to aid in evaluating relationships among the genera, to facilitate the identification of modern and fossil representatives, and to inform considerations of fruit adaptation in relation to climate. In a forthcoming treatment, we intend to review the fossil fruit record of spondioid Anacardiaceae in relation to what we have learned from this survey of the living members.

## Materials and Methods

Fruit characters of all genera, and most species, of Spondioideae were investigated (Table 1) and compared with reference to previous studies by Grote (1989), Mitchell et al. (2006), Bachelier & Endress (2009), and Mitchell (2015) as well as Von Teichman (1987, 1990, 1992) and others. We also included the genus *Campnosperma*, which is sister to a clade that includes both Spondioideae 2 and Anacardioideae in the analyses of Weeks et al. (2014).

**Table 1:** Voucher specimen data for fruits examined in this study

A	*Allospondias lakonensis* Stapf	C. Wang 33,758	Hainan	China
A	*Allospondias lakonensis* Stapf	F.C. How 73,253	Hainan	China
A	*Allospondias lakonensis* Stapf	J.F. Maxwell 97–833	Muang: Doi Luang National Park	Thailand
F 222209	*Allospondias lakonensis* Stapf	D.D. Soejarto & N.M. Cuong		Vietnam
OSA 3236	*Allospondias lakonensis* Stapf	Miki & Sakamoto		Philippines
US 1670086	*Allospondias lakonensis* Stapf	C. Wang 33,151	Hainan	China
UC 243708	*Allospondias lakonensis* Stapf	H.H. Chung 3066	Foochow, Fujian	China
K	*Allospondias laxiflora* Lace	Murray s.n.		Myanmar
US 3017984	*Antrocaryon amazonicum* (Ducke) B.L. Burtt & A.W. Hill	T. Plowman et al. 8823	Pará	Brazil
US 2439182	*Antrocaryon amazonicum* (Ducke) B.L. Burtt & A.W. Hill	Lemos Fróes 20,295	Pará	Brazil
NY	*Antrocaryon klaineanum* Pierre	J.M. Reitsma 1978		Gabon
K 53/1	*Antrocaryon micraster* A. Chev. & Guillaumin	M.T. Dawe 797	Budongo Forest	Uganda
K	*Attilaea abalak* E. Martinez & Ramos	E. Martínez & D. Alvarez 30,821	Campeche	Mexico
NY	*Buchanania arborescens* (Blume) Blume	C. Niyomdham 1687	Narathiwat, Paawai	Thailand
US 43698	*Buchanania arborescens* (Blume) Blume (syn.: *florida* Schauer)	E.D. Merrill 1987	Luzon	Philippines
F 2078990	*Buchanania sessifolia* Blume	E. Soepadmo & Suhaimi 5139	Kelantan, Pasir Mas	Malaysia
P 6634080	*Campnosperma micranteium* Marchand	R. Capuron 9013		Madagascar
US 1317201	*Campnosperma panamense* Standl.	G.P. Cooper & G.M. Slater 154	Bocas del Toro: Changuinola Valley	Panama
UF 1117	*Choerospondias axillaris* (Roxb.) B.L. Burtt. & A.W. Hill	B.R. Moore et al. s.n.	Khao Yai National Park	Thailand
UF 1166	*Choerospondias axillaris* (Roxb.) B.L. Burtt. & A.W. Hill	D.L. Dilcher s.n.	Gainesville, Florida	United States
UF 1253	*Choerospondias axillaris* (Roxb.) B.L. Burtt. & A.W. Hill	Kokawa & Manchester	Botanical Garden of Osaka City Univ. Kisaichi, Katano City, Osaka Pref.	Japan
UF 2552	*Choerospondias axillaris* (Roxb.) B.L. Burtt. & A.W. Hill	S.R. Manchester s.n.	Xishuangbanna Tropical Botanical Garden, Yunnan	China
UF 1038	Choerospondias axillaris (Roxb.) B.L. Burtt. & A.W. Hill	S.R. Manchester s.n.	Gasoline filling station, Xichou, Yunnnan	China
US 3396073	*Cyrtocarpa caatingae* J.D. Mitch. & Daly	D. Alvarenga et al. 1291	Goiás	Brazil
NY	*Cyrtocarpa caatingae* J.D. Mitch. & Daly	Cardoso et al. 525	Bahia: Quijingue	Brazil
NY	*Cyrtocarpa caatingae* J.D. Mitch. & Daly	A. Carvalho et al. 3762	Bahia: Caetité	Brazil
NY	*Cyrtocarpa caatingae* J.D. Mitch. & Daly	Tameirão Neto 3278 (BHCB 69724)	Minas Gerais: Berilo	Brazil
NY	*Cyrtocarpa caatingae* J.D. Mitch. & Daly	A. Carvalho et al. 3776	Bahia: Caetité	Brazil
UC 703692	*Cyrtocarpa edulis* (Brandg.) Standl.	J.T. Howell 10,616	Baja California Sur: Cabo San Lucas	Mexico
K 81604	*Cyrtocarpa edulis* (Brandg.) Standl.	A. Carter 2460	Baja California Sur	Mexico
F 2120859	*Cyrtocarpa edulis* (Brandg.) Standl.	T.S. Elias et al. 10,714	Baja California Sur	Mexico
UC 125741	*Cyrtocarpa procera* Kunth	C.A. Purpus s.n.	Puebla: Santa Lucia	Mexico
MO 3487625	*Cyrtocarpa procera* Kunth	J. Garcia P. 1013 & A. Delgado	Guerrero	Mexico
F 2067768	*Cyrtocarpa procera* Kunth	G. Flores 1064		Mexico
US 1269540	*Cyrtocarpa procera* Kunth	B.P. Reko 4967	Guerrero: Achotla	Mexico
MO 4908162	*Cyrtocarpa velutinifolia* (R.S. Cowan) J.D. Mitch. & Daly	B. Hoffman 1071 & H. Jacobs	Upper Takutu-Upper Essequibo Region: NW of Karasabai village	Guyana
US 3344305	*Cyrtocarpa velutinifolia* (R.S. Cowan) J.D. Mitchell & Daly	T.W. Henkel & R. James 3562	Upper Takutu-Upper Essequibo Region: South Rupununi Savanna	Guyana
K	*Cyrtocarpa velutinifolia* (R.S. Cowan) J.D. Mitchell & Daly	M.J. Jansen-Jacobs et al. 2120	Rupununi Distr.	Guyana
US 3006219	*Dracontomelon costatum* Blume	A.J.G.H. Kostermans 13,229	East Kalimantan	East Indonesia
NY	*Dracontomelon dao* (Blanco) Merr. & Rolfe	A.D.E. Elmer 8307	Luzon: Laguna	Philippines
NY	*Dracontomelon dao* (Blanco) Merr. & Rolfe	D.D. Soejarto et al. 7822	Luzon	Philippines
A	*Dracontomelon dao* (Blanco) Merr. & Rolfe	s.n.	Luzon	Philippines
NY	*Dracontomelon dao* (Blanco) Merr. & Rolfe	M. Ramos Edano 75,781	Luzon: Cantanduanes	Philippines
UF 1434	*Dracontomelon dao* (Blanco) Merr. & Rolfe	S.K. Pell 807- BKL	Hawaii: Kauai, National Tropical Botanical Garden	United States
UF 2497	*Dracontomelon duperreanum* Pierre	Xiaoyan Liu s.n.	Guangzhou: S. China Botanical Garden	China
UF 2546	*Dracontomelon macrocarpum* H.L. Li	Zhekun Zhou s.n.	SW Yunnan	China
K H2001/ 02777	*Haematostaphis barteri* Hook.f.	N.C. McLeod 840		Ghana
MO 2284758	*Haematostaphis barteri* Hook.f.	Latilo et al. 69,351	Kwara: Ilorin	Nigeria
XTBG 2481	*Haplospondias brandisiana* (Kurz) Kosterm. (syn.: *haplophylla* (Airy Shaw & Forman) Kosterm.)	Jianwu Li 847	Yunnan: Puer City,	China
MO 1606941	*Harpephyllum caffrum* Bernh. ex Krauss	I.F. Stebbins 133	Western Cape: Wellington	South Africa
MO 5655228	*Harpephyllum caffrum* Bernh. ex Krauss	A. Booi 188	Eastern Cape: Grahamstown	South Africa
NY	*Harpephyllum caffrum* Bernh. ex Krauss	D. Clemens 258	California: San Diego	United States
K	*Harpephyllum caffrum* Bernh. ex Krauss	carpological collection 6247	Gauteng: Pretoria	South Africa
BARC 162	*Harpephyllum caffrum* Bernh. ex Krauss	C.P. Lounsbury	West Cape: Cape Town	South Africa
NY	*Harpephyllum caffrum* Bernh. ex Krauss	J. Lau 1588	Hawaii	United States
A	*Koordersiodendron pinnatum* (Blanco) Merr.	Soepadmo 100	Kalimantan	Indonesia
NY 02100053 & F 2023855	*Koordersiodendron pinnatum* (Blanco) Merr.	D.D. Soejarto et al. 6571	Palawan	Philippines
F	*Lannea acida* A. Rich.	B.O. Daramola s.n.		Nigeria
P 5285392	*Lannea antiscorbutica* (Hiern) Engl.	A.R. Torre & M.F. Correia 16,198	Zambézia	Mozambique
P 5284657	*Lannea barteri* (Oliv.) Engl.	A. Chevalier 22,546		Ivory Coast
NY	*Lannea coromandelica* (Houtt) Merr.	D.B. Sumithraarachchi 741		Sri Lanka
F 2243252	*Lannea coromandelica* (Houtt) Merr.	W. Meijer 193		Sri Lanka
US 258427A	*Lannea coromandelica* (Houtt) Merr.	C.J. Saldanha 16,725	Hassan District: Karnataka	India
A	*Lannea coromandelica* (Houtt) Merr.	S.K. Lau 5852	Hainan	China
P 5191049	*Lannea coromandelica* (Houtt) Merr.	M. Perrottet s.n.	Puducherry: Pondicherry	India
UC 1037609	*Lannea coromandelica* (Houtt) Merr.	W. Koeltz 20,545	Uttarakhand: Kumaon, Harsila	India
UC 1419530	*Lannea edulis* (Sond.) Engl.	J. Pawek 59,538		Malawi
UC 1169187	*Lannea fulva* (Engl.) Engl.	R.E.S. Tanner 1184	Lake Province: Juma Island	Tanzania (ex-Tanganyika)
US 2480904	*Lannea schimperi* (A. Rich.) Engl.	W. Burger 2596	Harar Province	Ethiopia
K 452108	*Lannea velutina* A. Rich.	A.J.M. Leeuwenberg 4375		Burkina Faso
P 4852302	*Lannea velutina.* A. Rich.	P. Jaeger 5025		W. Africa
UC 1284129	*Lannea welwitschii* (Hiern) Engl.	A.J.M. Leeuwenberg 3755		Ivory Coast
MO 6605509	*Operculicarya calcicola* Randrian. & Lowry	L.P. Gautier et al. 5829	Mahajanga (Majunga) Province	Madagascar
MO 3662939	*Operculicarya decaryi* H. Perrier	P.B. Phillipson 2525	Toliara Province	Madagascar
MO 3662888	*Operculicarya decaryi* H. Perrier	P.B. Phillipson 2315	Toliara	Madagascar
US 2888387	*Operculicarya decaryi* H. Perrier	F.R. Fosberg 52,472	Toliara	Madagascar
K	*Operculicarya hyphaenoides* H. Perrier	R. Capuron (SF) 20,614	Toliara	Madagascar
MO 6446890D	*Operculicarya multijuga* Randrian. & Lowry	R. Rakotondrajaona 398	Antsiranana	Madagascar
MO 5794982	*Operculicarya* sp.	R. Rabevohitra et al. 4263	Antsiranana	Madagascar
NY	*Pegia nitida* Colebr.	Henry 11729A	Yunnan	China
P 6634428	*Pegia sarmentosa* (Lecomte) Hand.-Mazz.	L’Abbé Bon 2679 (HN)		Vietnam
K	*Pegia sarmentosa* (Lecomte) Hand.-Mazz.	L. Lugas 789	Sabah	Malaysia
GH	*Pleiogynium hapalum* A.C. Sm.	A.C. Smith 1499	Vanua Mbalava	Fiji
US 2190144	*Pleiogynium hapalum* A.C. Sm.	A.C. Smith 7164	Viti Levu	Fiji
A	*Pleiogynium timoriense* (DC.) Leenh.	H.S. McKee 40,734		
UF 1341	*Pleiogynium timoriense* (DC.) Leenh.	Mike Pole s.n.	Cultivated University of Queensland	Australia
UF 1433	*Pleiogynium timoriense* (DC.) Leenh.	S.K. Pell 802- BKL	Hawaii: Kauai, National Tropical Botanical Garden	United States
NY 40077	*Pleiogynium timoriense* (DC.) Leenh.	P.A. Cox 1113	Island of Eua	Tonga
FLAS 158900	*Pleiogynium timoriense* (DC.) Leenh.	W.L. Stern s.n.	Cultivated, Homestead, Florida	United States
P JU-15906	*Poupartia borbonica* J.F. Gmel.	Holotype: Commerson s.n.	Bois de Poupart	Réunion Island
P 295773	*Poupartia borbonica* J.F. Gmel.	Isotype: Commerson s.n.	Bois de Poupart	Réunion Island
MPU 020599	*Poupartia borbonica* J.F. Gmel.	Isotype: Commerson s.n.	Circa St Paul, Legot	Réunion Island
MSB 523570	*Poupartia borbonica* J.F. Gmel.	P. Seepaul 365	Roches Noires	Mauritius
NY	*Poupartia chapelieri* (Guillaumin) H. Perrier	Ravelonarivo & Rabesonina 674	Antsiranana	Madagascar
K	*Poupartia chapelieri* (Guillaumin) H. Perrier	L.M. Randrianjanaka et al. 603	Toamasina	Madagascar
P 00120505	*Poupartia gummifera* (Sprague) Capuron	SF/CTFT 21070	Morondava, Menabe	Madagascar
K	*Poupartia gummifera* (Sprague) Capuron	O. Andrianantoanina & R. Bazara 904	Antsiranana	Madagascar
K	*Poupartia minor* (Bojer) L. Marchand	M. Adriamahay & S. Rakotoarisoa 2350	Toliara	Madagascar
P	*Poupartia minor* (Bojer) Marchand	C. Bourgeois 67	Toliara	Madagascar
P 5198195	*Poupartia minor* (Bojer) L. Marchand	M.H. Perrier de la Bathie 12,784	Toliara	Madagascar
P 5198200	*Poupartia minor* (Bojer) L. Marchand	R. Ranaivojaona 483	Toliara	Madagascar
P	*Poupartia minor* (Bojer) L. Marchand	M.H. Humbert [carpological collection]		Madagascar
MO 2766559	*Poupartia orientalis* Capuron ex Randriamasolo & J.S. Miller	A. Randrianasolo et al. 1033	Toamasina	Madagascar
MO 5666318	*Poupartia orientalis* Capuron ex Randrianasolo & J.S. Miller	N.M. Andrianjefy et al. 292	Toamasina	Madagascar
K	*Poupartia orientalis* Capuron ex Randrianasolo & J.S. Miller	L. Nusbaumer & P. Ranirison, LN 1510	Antsiranana	Madagascar
K M.1344/79 4	*Poupartia pubescens* Marchand	Horne 4/75		Mauritius
K H. 1344/79 3	*Poupartia pubescens* Marchand	M. Mouton		not indicated
K	*Poupartia sahafariensis* Capuron in sched., nomen nudum.	F.D. 7016SF	Sakoala, Sahafari-Diego	Madagascar
MO 6088121	*Poupartia silvatica* H. Perrier	R. Ranaivojaona et al. 1706	Toliara, Tulear II, belalanda, Ranobe	Madagascar
MO 6605509	*Poupartia silvatica* H. Perrier	L.P. Gautier et al. 5829	Province de Majunga/ Mahajanga, Region Melaky	Madagascar
F 2287148	*Poupartia silvatica* H. Perrier	G.E. Schatz 4229		Madagascar
MO 3191961	*Poupartia silvatica* H. Perrier	S. Randrianasolo et al. 540	Toliara	Madagascar
NY	*Poupartia* sp.	S. Malcomber et al. 1911	Ankarana	Madagascar
MO 6443312	*Poupartia* sp.	Tefy Andriamihajarivo et al. 1387	Antsiranana	Madagascar
MO 4816066 & P 571141	*Poupartiopsis spondiocarpus* Capuron ex J.D. Mitch. & Daly	Capuron (SF) 18,188	Toamasina	Madagascar
MO 4848710	*Poupartiopsis spondiocarpus* Capuron ex J.D. Mitch. & Daly	Anonymous 14,988-SF		Madagascar
P 5198123	*Poupartiopsis spondiocarpus* Capuron ex J.D. Mitch. & Daly	Service Forestier Madagascar s.n.		Madagascar
P	*Poupartiopsis spondiocarpus* Capuron ex J.D. Mitch. & Daly	Capuron 8930-SF	Tenina	Madagascar
NY	*Pseudospondias longifolia* Engl.	J. Reitsma 1339		Gabon
MO 3097751	*Pseudospondias microcarpa* (A. Rich.) Engl.	W. Meijer 15,034	Yaoundé	Cameroon
NY	*Pseudospondias microcarpa* Engl.	D.W. Thomas & J.M. Fay 7284		Central African Republic
UF 1431	*Pseudospondias microcarpa* Engl.	A. Randrianasolo 809		Gabon
UC 1340029	*Pseudospondias microcarpa* Engl.	A. Leonard 2212		Congo
US 3497063	*Sclerocarya birrea* (A. Rich.) Hochst. subsp. *caffra* (Sond.) Kokwaro	J.S. Miller 10,666	Antsiranana	Madagascar
UF 2535	*Sclerocarya birrea* (A. Rich) Hochst	S.R. Manchester s.n.	Cultivated, Brisbane Botanical Garden	Australia
MO 6087493	*Sclerocarya birrea* subsp. *caffra* (Sond.) Kokwaro	R. Rabevohitra et al. 4543	Antsiranana	Madagascar
A	*Sclerocarya birrea* subsp. *caffra* (Sond.) Kokwaro	E.H. Wilson s.n.		Zimbabwe
K H2725/60259	*Sclerocarya birrea* subsp*. caffra* (Sond.) Kokwaro	A.A. Bullock 1316	Kaputa,	Zambia
K 2002/03216 68/68	*Sclerocarya gillettii* Kokwaro	J.B. Gillett 21,245		Kenya
F 1463003	*Sclerocarya birrea* subsp. *caffra* (syn.: *schweinfurthiana* Schinz)	R.J. Rodin 2622		Namibia
UC 1342531	*Solenocarpus indicus* Wight & Arn.	Thakur Rup Chand 2938	Assam	India
A	*Solenocarpus philippinensis* (Elmer) Kosterm.	J.S. Burley et al. 4276	Sulawesi: Utara	Indonesia
NY	*Solenocarpus philippinensis* (Elmer) Kosterm.	A.J.G.H. Kostermans 4661	Kalimantan	Indonesia
US 3002912	*Solenocarpus philippinensis* (Elmer) Kosterm.	A.J.G.H. Kostermans 10,502	East Kalimantan	Indonesia
US 2450012	*Solenocarpus philippinensis* (Elmer) Kosterm.	D.R. Mendoza 42,448	Mindanao	Philippines
UF 2219	*Spondias bipinnata* Airy Shaw & Forman	S.R. Manchester s.n.	Lop Buri Province: Chai Badan Distr.	Thailand
GH	*Spondias bipinnata* Airy Shaw & Forman	S. Chongko 675	Kanchanaburi Prov: Sai Yok Distr.	Thailand
UF 163	*Spondias dulcis* Parkinson	P. Grote s.n.	Store Bought Bangkok	Thailand
NY	*Spondias globosa* J. D. Mitch. & Daly	A.H. Gentry 68,896	Madre de Dios: Tambopata	Peru
NY	*Spondias macrocarpa* Engl.	W.W. Thomas & T. D. Pennington 6823	Bahia	Brazil
UF1594	*Spondias mombin* L.	V. Call s.n.	Flagler Beach, Florida	United States
UF 1157	*Spondias mombin* L.	D.L. Dilcher s.n.	Botanical Garden of Rio de Janeiro	Brazil
UF 1601	*Spondias mombin* L.	V. Call s.n.	Florida: Flagler Beach	United States
UF 1603	*Spondias mombin* L.	V. Call s.n.	Florida: Flagler Beach	United States
UF 38	*Spondias* cf. *mombin* L.	D.L. Dilcher s.n.	Guanacaste	Costa Rica
UF 40	*Spondias pinnata* (L.f.) Kurz	P. Grote 162	Market, Bangkok	Thailand
GH	*Spondias pinnata* (L.f.) Kurz	D.K. Harder et al. 5685	Lai Chau	Vietnam
GH	*Spondias purpurea* L.	E. Palmer 408	Jalisco	Mexico
UF 1435	*Spondias purpurea* L.	S.K. Pell s.n.		Costa Rica
US 3107537	*Spondias radlkoferi* Donn. Sm.	M.J. Balick et al. 1824	Cayo District	Belize
UF 1451	*Spondias* sp.	S.R. Manchester		Panama
UF 1187	*Spondias* sp.	D.L. Dilcher	Gamboa, Canal Zone	Panama
UC 1420517	*Tapirira guianensis* Aubl.	J. de Bruijn	Border between Departamentos Antioquia and Bolívar	Colombia
UC 1442206	*Tapirira guianensis* Aubl	Hatschbach 41,738	Paraná: Bairro Alto	Brazil
UC 252334	*Tapirira guianensis* Aubl	Klein 9634	Santa Catarina	Brazil
P 5190727	*Tapirira guianensis* Aubl	Aubreville 388	Riv. Compte’	French Guiana
NY	*Tapirira guianensis* Aubl	G. Herrera 2281	Talamanca	Costa Rica
US 2780052	*Tapirira obtusa* (Benth.) J.D. Mitch. (syn.: *marchandii* Engl.)	M. Kuhlmann 757	São Paulo	Brazil
MO 2088147	*Tapirira myriantha* Triana & Planch.	G. Davidse & A.C. Gonzales 13,772	Miranda	Venezuela
GH	*“Tapirira” mexicana* Marchand	R. Hernández M. 01615	Veracruz	Mexico
MO 4590657	*“Tapirira” mexicana* Marchand	W. Haber & W. Zuchowski 8726	Monteverde,	Costa Rica
MO 50454832	*“Tapirira” mexicana* Marchand	T. Wendt et al. 5326	Chiapas: Altamirano	Mexico
NY	*“Tapirira” mexicana* Marchand	B. Gutiérrez 3557	Veracruz	Mexico
NY	*“Tapirira” mexicana* Marchand	Z. Fuentes 327	Puntarenas	Costa Rica
A	*“Tapirira” mexicana* Marchand	M. Martínez 2	Veracruz	Mexico
GH	*“Tapirira” mexicana* Marchand (syn.: *macrophylla* Lundell)	D.E. Breedlove 53,500	Chiapas: La Trinitaria	Mexico

Endocarp surface morphology was revealed by boiling the dried fruits in water and physically removing the rehydrated exocarp and/or mesocarp by hand. Internal fruit structure was generally revealed by physical sections made with a Microslice II annular diamond saw with minimal kerf loss of about 30 μm from the path of the blade, or in some instances using a 2.5 diameter cm circular diamond blade mounted on a hand drill. Where physical sectioning was not permitted, or where it was especially valuable to examine multiple planes of section in a single specimen, X-ray datasets were obtained for many specimens using a GE Vtome-xm 240 Nano CT scanner at the Nanoscale Research facility at the University of Florida. Voltages, current, and timing were varied according to the resolution desired. The University of Montpellier micro CT (Skyscan 1076) was used for the *Poupartia borbonica* isotype only. The accelerating voltage was 40Kv, current of 250 μA and exposure time of 1100 ms giving a scan time of 72 min. The theoretical pixel size is 9 μm. The SRXTM facility of the TOMCAT beamline, Swiss Light Source, Paul Scherrer Institut, Villigen, Switzerland was used for *Lannea velutina* only. For SRXTM the specimen was wrapped in polyester batting, placed in a plastic tube and imaged using a 300 μm thick LuAg:Ce scintillator screen (FEE, Idar-Oberstein, Germany) to convert X-rays into visible light. Samples were scanned using a X1.25 microscope objective, digitized by a sCMOS camera (PCO.edge 5.5; PCO GmbH, Kelheim, Germany) with 18 keV and an exposure time per projection of 50 ms. The theoretical pixel size is 5.2 μm. Virtual sections and surface renderings were prepared from the CT datasets using Avizo 9 software.

Information on synonymies and geographic distribution was derived from regional floristic works, e.g., Airy-Shaw and Forman (1967), Hou (1978), Kostermans (1981, 1991, 1992), Kochummen (1996), Ashton (2003), Chayamarit (2010) for Tropical Asia and Malesia; Kokwaro (1986), Kokwaro and Gillett (1980) for Africa; Friedmann (1997) for the Mascarene Islands, as well as monographs and systematic treatments.

We studied extant genera of Spondioideae using fruiting specimens available in the herbaria of Field Museum (F), Kew (K), Missouri Botanical Garden (MO), Université de Montpellier (MPU), Muséum National d’Histoire Naturelle (P), New York Botanical Garden (NY), United States National Herbarium (US), United States National Seed Herbarium (BARC), University of California, Berkeley (UC), University of Florida (FLAS), Xishuangbanna Tropical Botanical Garden, Yunnan (XTBG), and the modern fruit and seed reference collection of the Paleobotanical Collection of the Florida Museum of Natural History at the University of Florida, Gainesville (UF). Additional material was collected from South China Botanical Garden, Guangzhou, and Xishuangbanna Tropical Botanical Garden, Yunnan. Preparations of extant species were also studied by transmitted and reflected light in the S. Miki collection of Osaka Museum of Natural History (OSA). Collection information for extant taxa in plate captions is presented as: (herbarium abbreviation, herbarium number, collector number), country. The authorities for all binomials used here are presented in the listing of specimens examined (Table 1).

## Terminology

### Planes of Section

To decipher internal fruit morphology and anatomy, we relied on physical and sometimes virtual sections in different orientations. Transverse sections perpendicular to the axis of symmetry were usually done “equatorially,” approximately halfway between base and apex, but sometimes additional cuts closer to the base or apex were made to intercept the germination openings and/or opercula or valves and to understand the positioning of lacunae. Longitudinal sections in the figures can be described more specifically as sagittal, or coronal. The *sagittal* section coincides with the plane of symmetry (bilateral or radial) of a fruit, or of a particular locule, while the *coronal* plane is at right angles to the sagittal section and divides the fruit or locule into dorsal and ventral (back and front) portions. The sagittal section of the locule typically shows the extent to which it is curved or straight, while the coronal section may show the shape of the apical keel if present.

### Pericarp Layers

We treat the fruits of this group uniformly as drupes with a single hard endocarp or “stone” (sclerocarp) representing one or more carpels, rather than speaking of multiple endocarps per fruit. The endocarp is surrounded by a soft, fleshy, or leathery mesocarp and a thin-skinned exocarp. Some disagreements exist regarding the terminology for different layers of the pericarp in Spondioideae fruits. Standard fruit nomenclature used for angiosperms (e.g., Roth, 1977) recognizes just three layers of the fruit wall, or pericarp, i.e., endocarp, mesocarp, and exocarp. Spondioideae fruits may possess four or five discrete layers (e.g., *Pleiogynium*, *Spondias*). Should just one of these layers be treated as endocarp, followed by inner and outer mesocarp, or should the “endocarp” be treated as two layers, inner and outer? We consider the outer epidermis (and some underlying cell layers per Von Teichman investigations) to be the exocarp, and the fleshy or parenchymatous part as mesocarp. We refer to the sclerenchymatous stone as the endocarp; but others have treated the outer part of the stone as mesocarp. Von Teichman and Hardy (1992) noted that the outer and inner epidermis of the mature pericarp are readily recognized, and treat these as the exocarp and endocarp (*s.s*. [sensu stricto]), considering everything in between as mesocarp (*s.s.*). However, they recognized that mesocarp, defined in this way, includes part of the stone, “which forms a natural, functional unit and as such represents the endocarp *s*.*l*. [sensu lato].” For consistency among the descriptions presented here, we recognize the following four layers:

1. *Locular envelope*. This is a layer of dense sclerenchyma tissue immediately surrounding the locule, referred to as the endocarp sensu stricto by Von Teichman and Hardy (1992), and as inner endocarp by Rozefelds et al. (2015). Developmental studies indicate that this layer represents the inner epidermis of the gynoecium. Depending on the genus, composition of the locular envelope may be of circumferential fibers, or isodiametric sclereids, or both. In multilocular fruits the locular envelopes can be largely free from one another, but partially to completely fused to adjacent envelopes in the septal area.
2. *Endocarp s*.*l*. (*sclerocarp*) is the predominantly sclerenchymatous layer that includes and surrounds the locular envelope(s), uniting them into a common structure known as the “stone.” This tissue, composed mainly of tortuous tracts of fibers, but sometimes also of brachysclereids, forms the main part of the septa as well as the external wall of the stone. In multicarpellate fruits, the endocarp may form a star-like and/or spiny configuration when seen in transverse sections. In some genera, the endocarp accommodates prominent lacunae, which often alternate with the locules in multilocular fruits.
3. *Mesocarp* (*sarcocarp*) is the soft or fleshy parenchymatous layer that makes these fruits attractive for animal dispersal, and is the part eaten also by people (e.g., *Choerospondias*, *Harpephyllum*, *Lannea*, *Sclerocarya*, and *Spondias*). Because of its ephemeral nature, this layer is commonly missing from fossil fruits, although there are some exceptions (e.g., Collinson et al., 2012; Herrera, 2014).
4. *Exocarp* is the cutinized layer of epidermal cells forming the outer layer of the pericarp. It may bear stomata and/or tanniniferous cells (Von Teichman & Hardy, 1992).

### Germination mechanisms

**Fig. 1:**
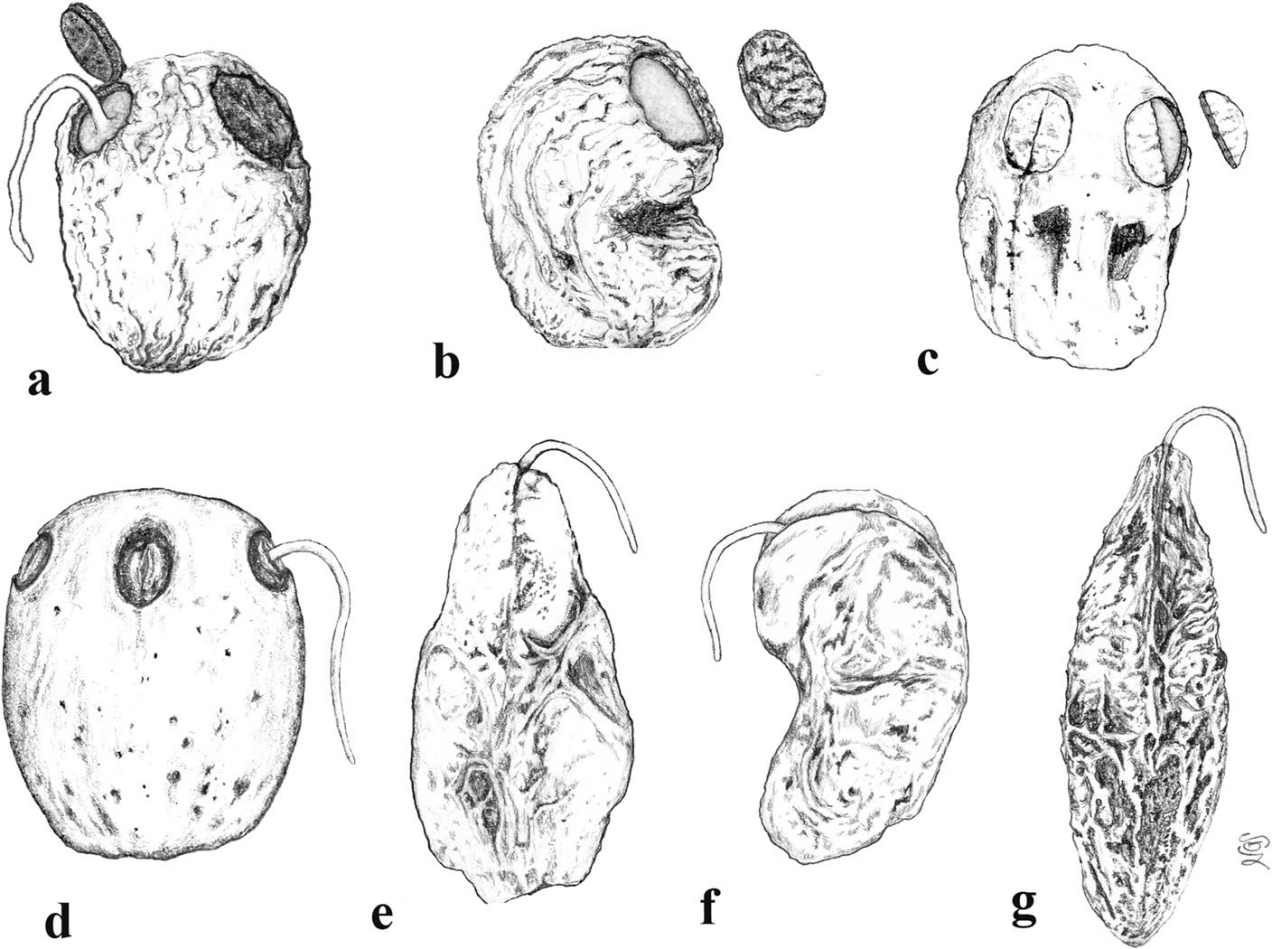
Drawings of germination mechanisms found in fruits of Spondioideae. **a** Simple opercula (cf. *Sclerocarya*). **b** Another example of simple operculum (cf. *Operculicarya*). **c** Bipartite opercula (cf. *Pseudospondias*). **d** Recessed bilabiate (cf. *Choerospondias*). **e, f,** Paired apical flaps (cf. *Solenocarpus,* cf. *Haplospondias*). **g** Apical slit (cf. *Koordersiodendron*)

Spondioid fruits are diverse in germination mechanisms that are specialized for the exit of the radicle through the stony endocarp (Fig. 1). For consistency among the descriptions presented here, we recognize the following germination modes--each with a characteristic morphology. We use the general term *pore*, to refer to the germination perforation which may, or may not, be operculate.

1. *Simple opercula* (Fig. 1a, b). Some endocarps have well-defined stopper-like plugs, usually at the apical end of each locule (but variable in position in *Cyrtocarpa*). These opercula are similar in thickness and cellular composition to the surrounding endocarp wall. Simple opercula occur in *Antrocaryon*, *Cyrtocarpa*, *Dracontomelon*, *Lannea*, *Operculicarya* (Fig. 1b), *Poupartia*, and *Sclerocarya* (Fig. 1a).
2. *Bipartite opercula* (Fig. 1c). In some genera, each locule of the fruit possesses an operculum formed of two symmetrical shutter-like plugs with a median plane of separation. This kind of bipartite germination mode was first noted by Hill (1933): “*the lid separates into two equal halves which are pushed apart and thrown aside by the radicle as it emerges*.” (Hill, 1933, p. 29). Such opercula are seen exclusively in *Pseudospondias* (Fig. 1c), *Haematostaphis*, and the extinct genus, *Pentoperculum* (Manchester, 1994). Contrary to earlier work, we maintain a distinction between endocarps with this kind of thick, bipartite plug-like operculum and, those with a bilabiate opening, where the apical keel of the locular envelope and/or endocarp splits open, allowing the radical to exit (see below).
3. *Recessed bilabiate* (Fig. 1d). Mitchell et al. (2006) recognized “internal germination opercula” in some of the genera, e.g., *Harpephyllum* and *Spondias*. We also examined these and found that, rather than a true stopper-like operculum, these genera possess an internal “bilabiate” dehiscence mechanism that has not been described in detail before. In this type of dehiscence mechanism, seen in *Allospondias*, *Attilaea***,** *Choerospondias* (Fig. 1d), *Pleiogynium*, *Spondias* (at least some), and “*Tapirira*” *mexicana*, the locular envelope opens along its apical keel, which is recessed within a pore of the endocarp wall. The locular envelope opens like a pair of lips, or clam-like valves, allowing for emergence of the radical through the external pore of the endocarp. These valves are usually cryptic, but can be seen within, or slightly protruding from, germination pores of the endocarp. They are composed of the locular envelope and do not seem to be homologous with the bipartite external opercula of *Haematostaphis* and *Pseudospondias*.
4. *External valves or flaps* (Fig. 1e, f). Fruits of *Haplospondias* (Fig. 1f) and *Solenocarpus* (Fig. 1e) have an external pair of apical valves. In these cases, the apical portion of the stone is thinner, and less ribbed/ornamented than the main part of the stone, forming a reniform flap that may hinge or break along its base to open apically at germination. Valves can be simply interpreted as an external extension of the locular envelope and its surrounding tissue.
5. *Apical slit* (Fig. 1g). A slit in the endocarp wall, where no pore or operculum is present, can provide another means of opening at germination. This characterizes *Koordersiodendron* (Fig. 1g), and possibly *Buchanania.*
6. *Unspecialized/not obvious*. In some cases, there is no obvious opening mechanism, and it is likely that the radicle might penetrate any part of the pericarp, as in the case of the thin-walled endocarp of *Pegia*.

### Shape of Germination Pore and/or Opercula

Typically, the germination pores and opercula are not circular but rather are elliptical or broadly elliptical. We describe the shapes of the germination pores or valves with reference to the symmetry of the endocarp on which they are borne. “Elliptical,” when used without qualification, refers to an ellipse that has its long axis aligned with the central axis of the fruit; with its length greater than its width. Broadly elliptical refers to a pore or operculum that is wider than high, with its long axis perpendicular to the central axis of the fruit.

### Lacunae

The presence or absence and configuration of empty cavities or spaces (lacunae) within the endocarp wall can also be diagnostic (Table 1). Lacunae are often present in the septal region of multilocular fruits, but absent or inconspicuous in unilocular fruits. They may be filled with parenchyma contrasting with the sclerenchymatous wall of the endocarp, or more commonly are empty at maturity. As viewed in equatorial section, there may be the same number of lacunae as locules, with lacunae alternating in position with the locules, or there may be twice as many lacunae as locules with a pair of lacunae flanking each locule. Some species have scattered irregular cavities in the wall, which we also treat as lacunae.

### Apertures

Dimples on the surface of the endocarp are often the external expression of small channels that penetrate the endocarp wall. Such apertures occur in a conspicuous basal whorl in some genera (e.g., *Antrocaryon*, *Choerospondias*, *Poupartia*, *Sclerocarya*) and in these instances, they alternate with the locules and connect directly with the lacunae. In other cases, the apertures are in a near equatorial whorl (*Dracontomelon*) or scattered over the periphery of the stone (e.g., *Choerospondias*, *Haematostaphis*, some species of *Cyrtocarpa*). We also describe *punctae* and *depressions* on the endocarp, i.e. sunken areas that do not perforate the endocarp. The depressions may be irregularly or symmetrically arranged.

## Survey of Extant Spondioid Genera

Here we present characters of fruit morphology for all genera of Spondioideae, plus *Campnosperma* in alphabetical order by genus. Table 2 provides endocarp dimensions, and summarizes selected morphological and anatomical characters that are useful for the recognition of living genera and fossil taxa.

**Table 2:** Comparison of selected fruit morphological & anatomical characters of for Spondioideae

Genus	Typical Maximum dimensions of endocarp (mm)	Endocarp shape & symmetry	Germination mechanism	Endocarp surface	Endocarp apertures	Locule number	Lacunae (at equator)	Locule outline, sagittal section	Locular envelope composition
*Allospondias*	16 L; 10 W	obovoid, prolate; radially symmetrical; rounded to polygonal in ts	apical pores, recessed bilabiate	finely reticulate & striate; with elongate lateral depressions	absent	2–5	present; same number as locules; can be large & circular	straight	1) inner layer ca. 5-seriate of horizontal fibers; 2) surrounding layer 0.2–0.3 mm, of isodiametric brachysclereids
*Antrocaryon*	14 L; 25 W to 22 L; 35 W	oblate to subglobose, radially symmetrical; circular to pentagonal in ts	simple plug-like opercula near apex	minutely reticulate to smooth with scattered dimples	whorl of small basal apertures alternating with locules	5	present; same number as locules	curved	0.2–0.4 mm: 1) brachysclereids few-seriate; 2) interwoven tracts of fibers
*Attilaea*	12 L; 8 W	prolate, obovoid; bisymmetrical; elliptical in ts	apical pore, recessed bilabiate	ribbed, reticulate	a few prominent near apex	1	present on both sides of locule	not seen in ls, but certainly elongate	0.1 to 0.2 mm: horizontal encircling fibers
*Buchanania*	8 L; 6 W	prolate, subglobose; bisymmetrical with dorsal keel, rounded to lensoidal in ts	not obvious, possibly splitting apically	smooth	absent	1	absent	straight	ca. 0.3–0.4 mm: variously oriented elongate brachysclereids
*Campnosperma*	10 L; 9 W	prolate, obovoid, rounded in ts	unspecialized/ not obvious	smooth	absent	1	one central cylindrical	strongly curved	lacking; endocarp of isodiametric sclereids
*Choerospondias*	23 L; 17 W	prolate; radially symmetrical; circular in ts	apical pores, recessed bilabiate	smooth & finely fibrous with scattered punctae	whorl of small basal apertures alternating with locules & scattered smaller apertures	5 (3–6)	present; 2× number of locules near equator	straight	0.2–0.8 mm: 1) isodiametric sclereids 1- to few-seriate; 2) 60 to 100 μm layer encircling horizontal fibers
*Cyrtocarpa* type 1 (*C. procera*, others)	20 L; 13 W	prolate; obovoid; asymmetrical; subcircular in ts	simple plug-like opercula apical & basal	smooth & minutely reticulate	absent	3–5	present	straight to slightly curved	0.2–0.3 mm: anticlinal fibers
*Cyrtocarpa* type 2 (*C. velutinifolia*)	15 L; 11 W	prolate, elliptical, bisymmetrical (with keel); elliptical in ts	simple plug-like operculum near apex	smooth, punctate	scattered	1	present	curved	<0.1 mm: encircling horizontal fibers
*Cyrtocarpa* type 3 (*C. caatingae*)	13 L; 5.5 W; 10D	prolate, elliptical; bisymmetrical (with keel)	apical pore, recessed bilabiate	ribbed, punctate	scattered	1	present on both sides of locule	curved	0.2–0.3 mm: isodiametric brachysclereids
*Dracontomelon*	25 L; 33 W	oblate to subglobose; radially symmetrical to strongly asymmetrical; circular to polygonal in ts	simple plug-like opercula in apical half of stone	smooth, ridged, grooved	10 equatorial depressions with channels to lacunae	5 (−4)	present; samenumber as locules	curved	0.3 mm: encircling horizontal fibers
*Haematostaphis*	18 L; 11 W	prolate, elliptical; bisymmetrical (with keel); subcircular in ts	apical bipartite (shutter-like) operculum	finely reticulate	scattered	1	absent	straight	0.25 mm: encircling horizontal fibers
*Haplospondias*	7 L; 3 W	prolate, elliptical; bisymmetrical (with keel); ~elliptical to lenticular in ts	apical, external bivalvate	reticulate & ribbed	absent	1	absent	straight	0.1 mm: a few rows of encircling horizontal fibers
*Harpephyllum*	25 L; 13 W	prolate, obovoid; bisymmetrical (with dorsal keel) to ~ radially symmetrical at base; ~irregularly rounded in ts	apical pores, recessed bilabiate	ribbed, reticulate	absent	1–2	irregular lacunae	slightly curved	0.5–1.0 mm: mixed anticlinal fibers & isodiametric brachysclereids
*Koordersiodendron*	30 L; 7 W; 18 D	prolate, bisymmetrical (with keel); elliptical to lenticular in ts	apical split	strongly fibrous with minor ribs	absent	1	absent	straight	ca. 0.07 mm: uniseriate encircling fibers; or) uni- to biseriate with slightly anticlinally elongate brachysclereids
*Lannea*	1.2 L; 4 W; 6 D	prolate, reniform, bisymmetrical (with keel); elliptical to lenticular in ts	simple plug-like operculum near apex	strongly reticulate to ribbed	sometimes scattered, minute	1	absent	weakly to strongly curved	0.15 to 0.2 mm: mostly horizontal & periclinal fibers
*Operculicarya*	11 L; 5 W; 7 D	prolate, irregularly ovoid to subglobose, asymmetrical; irregularly rounded to polygonal in ts	simple plug-like operculum near apex	~smooth, minutely fibrous	sometimes scattered, minute	1, rarely 2	absent	strongly curved	thin; 0.05 mm: brachysclereids
*Pegia*	13 L; 5 W; 7 D	prolate, obovoid; bisymmetrical (with keel); elliptical in ts	unspecialized or apical splitting	prominently ribbed & grooved in a reticulate pattern	absent	1, rarely 2	absent	straight	0.2–0.4 mm: 1 to 3 seriate brachysclereids
*Pleiogynium*	18 L; 29 W	oblate, globose, turbinate; radially symmetrical; rounded polygonal in ts	apical pores, recessed bilabiate	smooth to punctate, & basally radially striate or ribbed.	absent	5–12	present; same number as locules	curved	0.5 mm: periclinal, horizontally arranged fibers or elongate brachysclereids
*Poupartia* type 1 (e.g*., P. borbonica, P. gummifera*)	10 L; 5 W	prolate, obovoid; asymmetrical; irregularly pentagonal in ts	simple plug-like opercula/operculum near apex	irregularly ribbed & covered with small depressions	absent	1–3	absent?	straight to slightly curved	0.05 mm; very thin: isodiametric brachysclereids
*Poupartia* type 2 (*P. chapelieri*)	11 L; 6 W	prolate, bisymmetrical; elliptical in ts	simple plug-like opercula/operculum near apex	finely striate	absent?	2	absent	straight to curved	0.1 mm; isodiametric brachysclereids
*Poupartia* type 3 (*P. minor, P. orientalis, P. silvatica*)	17 L; 15 W	prolate to sub globose; ± radially symmetrical; circular to irregularly pentagonal in ts	simple plug-like opercula /operculum near apex	smooth	whorl of basal apertures alternating with locules	4–5	present; same number as locules	curved	0.1 mm: dense, encircling horizontal fibers & some brachysclereids
*Poupartiopsis*	63 L; 43 W	prolate, elliptical; radially symmetrical; circular in ts	apical pores, recessed bilabiate	conspicuously spinose, with fine, elongate hooked projections	absent	3–4	not obvious	curved	0.15–0.2 mm: encircling horizontal fibers
*Pseudospondias*	~17 L; 11 W	prolate, oblong, obovoid; bisymmetrical (with keel) to radially symmetrical; ~circular to polygonal in ts	apical bipartite (shutter-like) opercula	mostly smooth with prominent longitudinal ribs	absent	4–5 (1–6)	present, small, alternating with locules	straight	0.1–0.2 mm: encircling horizontal fibers
*Sclerocarya*	30 L; 17 W	prolate, obovoid-subglobose, oblate; bisymmetrical (without keel) to radially symmetrical; elliptical to subcircular in ts	simple plug-like opercula near apex	~smooth, minutely reticulate	whorl of 3–5 basal apertures alternating with locules	2–4	a pair of crescent-shaped lacunae flanking each locule, plus scattered irregular lacunae	straight to slightly curved	0.3–0.6 mm: encircling horizontal fibers
*Solenocarpus*	9 L; 5 W; 6 D	prolate, reniform; bisymmetrical (with keel); elliptical, lenticular in ts	apical, external bivalvate	minutely reticulate to strongly ribbed	absent	1	absent	straight	0.1 mm: encircling horizontal fibers, surrounded by 0.3–0.6 mm layer of isodiametric sclereids
*Spondias*	44 L; 33 W	oblate to prolate, ovoid to obovoid; radially symmetrical, circular to elliptical in ts	apical pores, recessed bilabiate	strongly reticulate, pitted, spinose, fibrous, smooth	apparently absent	5 (rarely fewer)	alternating with locules, in New World species containing longitudinal rows of orbicules	straight	0.1–0.3 mm: encircling horizontal fibers
*Tapirira*	25 L; 13 W; 16 D	prolate, bisymmetrical (with keel); lenticular to elliptical in ts	unspecialized/ not obvious	reticulate, finely ribbed	absent	1	absent	straight	0.15 to 0.5 mm: encircling fibers
*“Tapirira” mexicana*	22 L; 11 W; 12 D	prolate; bisymmetrical (with keel); elliptical in ts	apical pore, recessed bilabiate	longitudinally & obliquely ribbed	both apical & scattered	1	absent	straight	0.18 mm: brachysclereids,

*D* Depth, *L* Length, *W* Width, *ts* Transverse section

### *Allospondias* Stapf (Fig. 2)

This genus has two species in tropical Asia (Kostermans, 1981, 1991). The type species, *Allospondias lakonensis*, occurs in China (Fujian, Guangdong, Guangxi, Hainan, and Yunnan Provinces), Laos, Thailand, and Vietnam. *Allospondias laxiflora* occurs in Myanmar and Thailand. Pell et al. (2011) commented that *A. laxiflora* might represent a distinct genus due to differences in connation of the stylodia, for having only two locules, and the absence of lacunae in the fruit. Airy-Shaw and Forman (1967) treated this species as *Spondias laxiflora*.

**Fig. 2:**
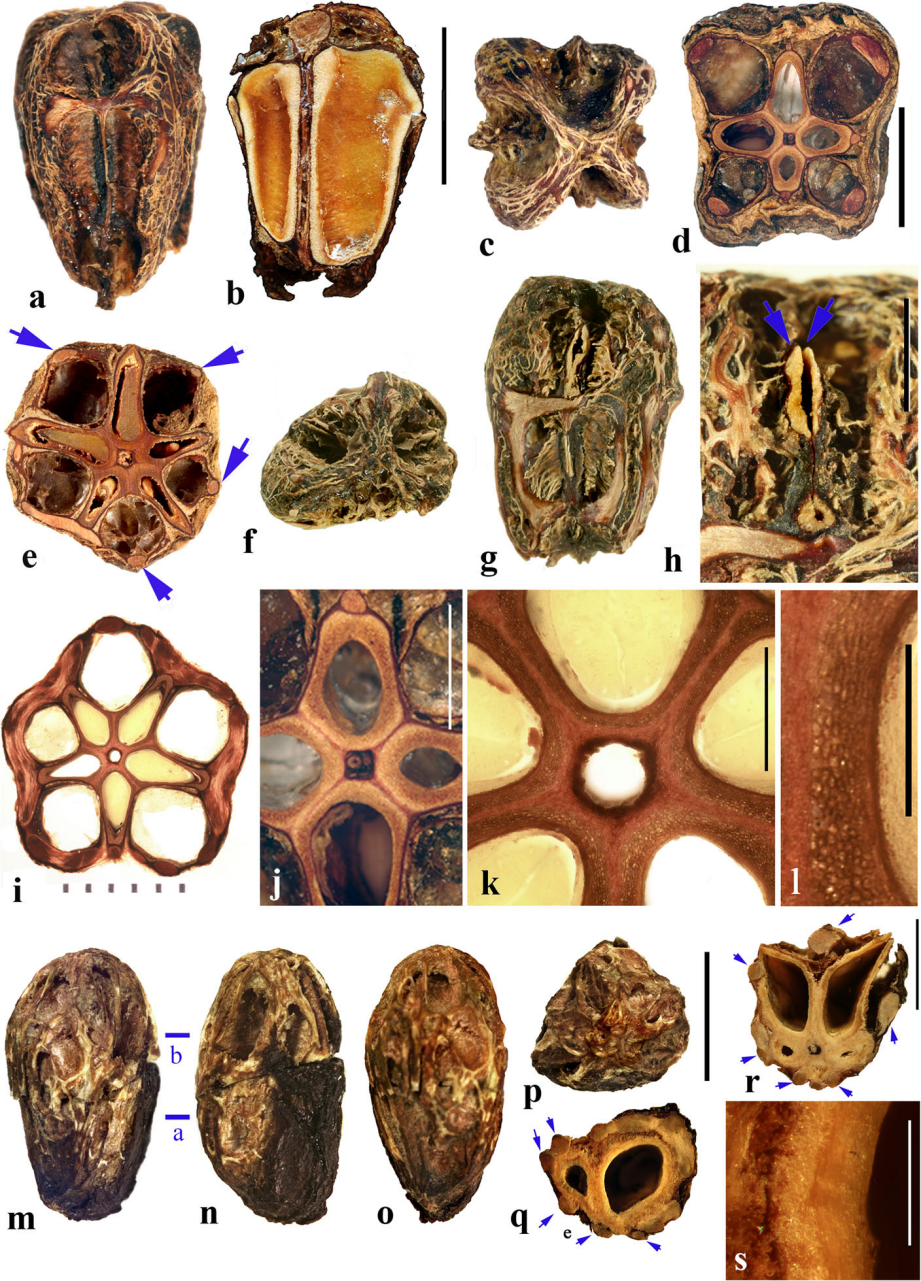
*Allospondias.* **a–l** *A. lakonensis***. a–c** Endocarp in lateral view, sagittal section and apical view indicating pores with recessed bilabiate valves (US 1670086: C. Wang 33,151; Hainan, China). **d** Typical tetralocular fruit in transverse section with intact meso- and exocarp showing four large lacunae alternating with locules (A: C. Wang 33,758; Hainan, China). **e** Pentacarpellate fruit in transverse section; arrows indicate terete fiber bundle at the peripheral edge of each locule (UC 243708: H.H. Chung 3066; Fukien, China). **f, g** Relatively rare trilocular specimen in apical and lateral views (F 222209: D.D. Soejarto and N.M. Cuong; Vietnam). **h** Detail of recessed bilabiate germination mechanism (arrows) from specimen in g. **i** Pentalocular specimen, transverse section by transmitted light. (OSA L3236: Miki & Sakamoto [1961, Pl 2 k b]; Philippines). **j** Detail from d showing thick locular envelopes. **k** Detail of septa from i. **l** Detail of locular envelope from k. **m–s** *Allospondias laxiflora* (K: Murray s.n.; Myanmar). **m–o** Lateral views of the same fruit rotated on its axis, **p** apical view. **q, r** Transverse sections at the levels indicated in a, b in fig. m; arrows indicate terete fiber bundles within the endocarp. Bilabiate locular opening is seen in r. **s** Magnification from q, showing locular envelope composed of fibers. Scale bars 5 mm in b (also applies to a), d (also applies to c, e**–**g), i and p (also applies to m**–**o); 2 mm in h, j; 3 mm in q, r; 1 mm in k; 0.5 mm in l, s

We studied several specimens of *Allospondias lakonensis* (Fig. 2a**–**l). The endocarp surface is finely reticulate and striate. The drupes are slightly obovoid, longer than wide (Fig. 2a, b, g). A pair of elongate surface depressions corresponds to each carpel in the lower 2/3 of the endocarp (Fig. 2a, g). *Allospondias* flowers are pentacarpellate (Mitchell et al., 2006), but most of the fruits we observed of *A. lakonensis* are tetracarpellate with four radially symmetrical locules (Fig. 2c, d, j)*,* although occasional tricarpellate (Fig. 2f) and pentacarpellate fruits (Fig. 2e, i, k) were seen (Table 1). The drupes appear rounded-triangular, to rounded-pentagonal in transverse section, commonly rounded-square in the case of tetralocular fruits. The locular envelopes are fused near the center of the drupe forming a 3 to 5-rayed star shape in cross section depending on the number of carpels (Fig. 2d, e, i). There was no obvious indication of aborted (smaller, squashed) locules in the specimens observed. The drupes have the same number of lacunae as locules. The lacunae are usually larger than the locules when seen in transverse sections near the equator and are nearly circular in transverse section (Fig. 2d, e, i). Sagittal sections of the drupes show that the long axes of the locules are straight and have a phalloid distal portion (Fig. 2b). Although previously reported as inaperturate, careful removal of the mesocarp reveals prominent shallow germination pores on the apical surface of the endocarp over each locule. The keel of the locular envelope protrudes through the pores, and appears to indicate internal recessed bilabiate germination (Fig. 2a, c, g, h).

The locular envelope in *Allospondias lakonensis* consists of two layers: a layer about 5 cells thick consisting of horizontal fibers about 8 μm in diameter, surrounded by a more prominent layer, 0.2**–**0.3 mm thick, of isodiametric brachysclereids 30**–**50 μm in diameter (Fig. 2j**–**l). Adjacent locular envelopes intersect with a well-defined radial suture plane (Fig. 2j), or are separated by a zone of parenchymatous tissue (Fig. 2k). Collectively, the locular envelopes are surrounded by a thin tannin-rich layer of compressed parenchyma and/or stone cells. Outside the locular envelopes the stone is composed mainly of ribs of tortuous fibers with nests of parenchyma. A terete bundle (seen as longitudinal ribs in sagittal and coronal sections), formed of densely packed fibers, occurs at the peripheral edge of each locule and of each lacuna, with the more prominent ribs of the surface of the endocarp aligned with the lacunae rather than with the locules.

We studied one collection of *Allospondias laxiflora* (K: Murray s.n., coll. March 1913; Fig. 2m**–**s). The drupes are obovoid. The endocarps have scabrate ornamentation, and unequal development of locules unlike *A*. *lakonensis* (Fig. 2m**–**p). The specimen we sectioned shows two enlarged fertile locules and three abortive ones (Fig. 2q, r). There are small collapsed areas between these locules filled with parenchyma that might represent lacunae, but they differ from the large ones seen in *A. lakonensis* (Fig. 2d, e, i). Removal of soft mesocarp material revealed two poorly defined pores without opercula; beneath each of these pores, the locular lining is exposed with a distal keel of bilabiate internal germination mechanism. The locular envelope is 0.4 mm thick composed of an inner layer of periclinal, horizontally oriented fibers and an outer layer composed of isodiametric brachysclereids (Fig. 2s). The surrounding tissue is composed mainly of parenchyma, but with large peripheral longitudinal terete fibrous strands, 0.5 to 1.1 mm in diameter.

### *Antrocaryon* Pierre (Fig. 3)

**Fig. 3:**
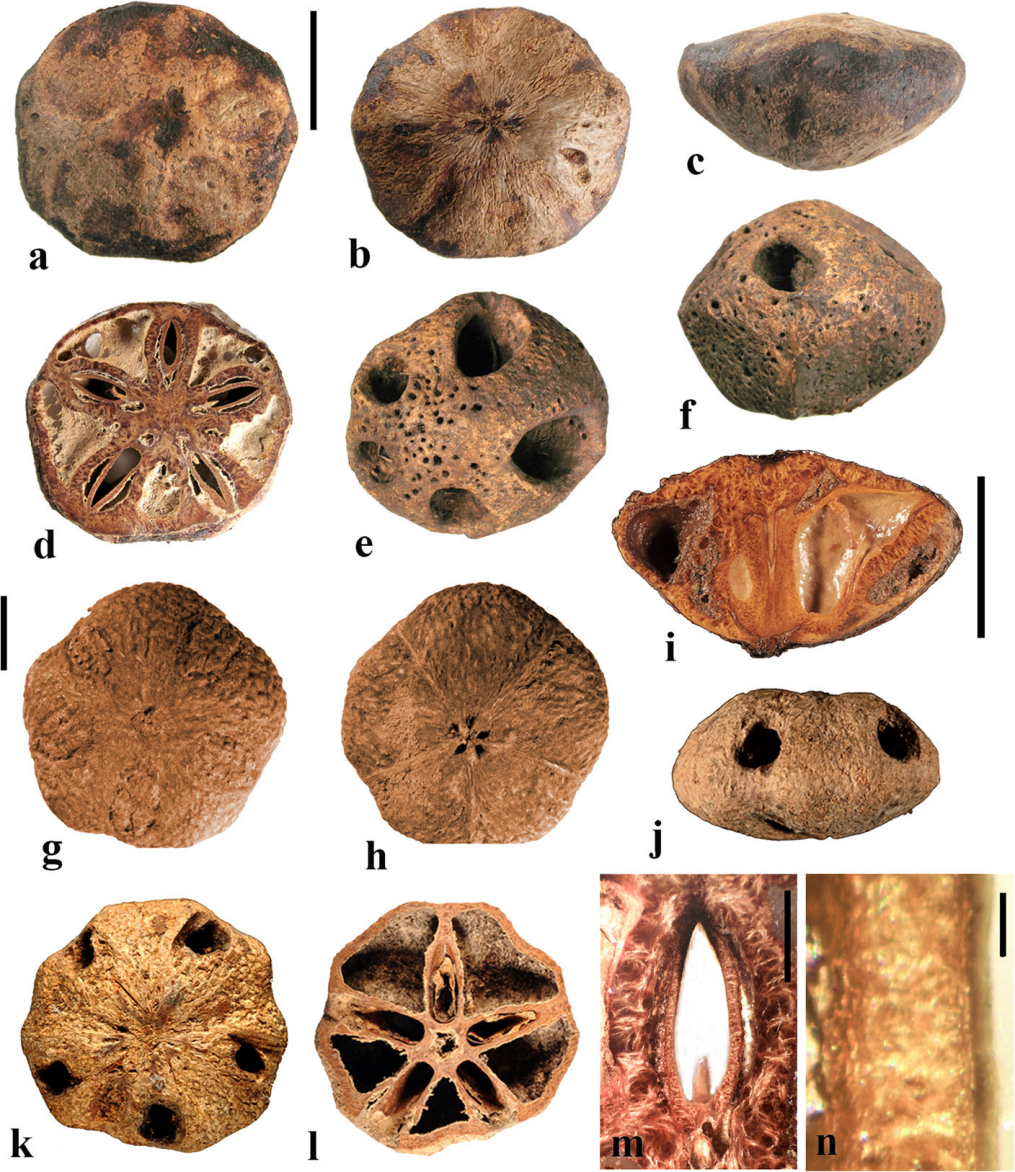
*Antrocaryon* fruits. **a–d** *A. klaineanum* endocarp in apical, basal, lateral views and in transverse section. Note 5 opercula in apical view and 5 basal apertures (NY: J.M. Reitsma 1978; Gabon). **d** Shows five locules arranged in a star pattern and five large lacunae. **e, f** *A. micraster* (K: M.T. Dawe 797; Uganda). **e** Apical view showing five locules with opercula removed. **f** Lateral view showing subapical position of locule opening. **g, h** Apical and basal views of *A. amazonicum* (BARC), note basal apertures in h. **i** Sagittal view of *A. amazonicum* (US 3017984: T. Plowman et al. 8823; Pará, Brazil). **j–l** *A. amazonicum* (US 2439182: Lemos Fróes 20,295; Pará, Brazil), endocarp in lateral and apical views with shed opercula, and transverse section. **m** *A. klaineanum*, detail of locule region, transverse section from d. **n** Detail of locular envelope from m. Scale bars 1 cm in a (also applies to b**–**f), i and g (also applies to h**–**l); 0.1 mm in m, n

This genus includes three or more species in tropical Africa plus one in tropical South America. We studied fruits of the type species, *Antrocaryon klaineanum* (Fig. 3a**–**d, m, n), and *A. micraster* (Fig. 3e, f) from Africa, and the South American species, *A. amazonicum* (Fig. 3g**–**l). Fossil fruits of *Antrocaryon* have been recognized from the Miocene (Tiffney et al., 1994) and Pliocene (Bonnefille & Letouzey, 1976) of Ethiopia, and from the Miocene of Kenya (Chesters, 1957).

The exocarp of *Antrocaryon* is yellowish when ripe and the mesocarp is fleshy, strong smelling, and edible (Pell et al., 2011). The stone is oblate, pentalocular, and radially symmetrical with five prominent, thick, simple plug-like opercula arranged in a circle on the apical surface midway between apex and equator of the stone (Fig. 3a, g, j, k). The endocarp surface ranges from minutely reticulate, fibrous to smooth and is occasionally covered with small and scattered punctae (Fig. 3e, f). A whorl of five depressions occurs at the base of the endocarp, alternating in position with the locules (Fig. 3b, h). The opercula are elliptical in outline. As viewed in equatorial transverse section, the stone appears almost circular to pentagonal with a prominent star-shaped structure formed by the radiating locular envelopes (Fig. 3d, l). Locules are elliptical in cross section and more or less reniform in sagittal section with a phalloid distal portion (Fig. 3i). In *A. klaineanum* and *A. amazonicum*, prominent lacunae alternate in position with the locules. These lacunae are triangularly shaped in equatorial section, and prominent (larger than the locules). However, in *A. micraster* (Fig. 3e), the stone is formed of more continuously sclerified tissue with irregularly shaped lacunae. The lacunae are typically empty at maturity.

The locular envelope (Fig. 3m, n), 0.2–0.4 mm thick, consists of a locule lining a few cell layers thick of brachysclereids (cells about 15 μm in diam.), surrounded by a dense tissue of interwoven tracts of fibers (individual fibers about 8 μm thick). The septa and outer wall of the stone, including the opercula (studied in *A. klaineanum* and *A. amazonicum*) are formed by a tissue distinct from the locular envelopes, composed mainly of tortuous fibers grading from obliquely periclinal near the center to mostly anticlinal near the periphery.

### *Attilaea* E. Martinez & Ramos (Fig. 4a–h)

This genus was established based on a single species, *Attilaea abalak*, from Yucatan, Mexico and Guatemala (Martínez & Ramos, 2007). Pell et al. (2011) treated it provisionally as *Spondias* but noted that more material needed to be examined to confirm the separate generic status. It differs from *Spondias* in having only one developed locule.

**Fig. 4:**
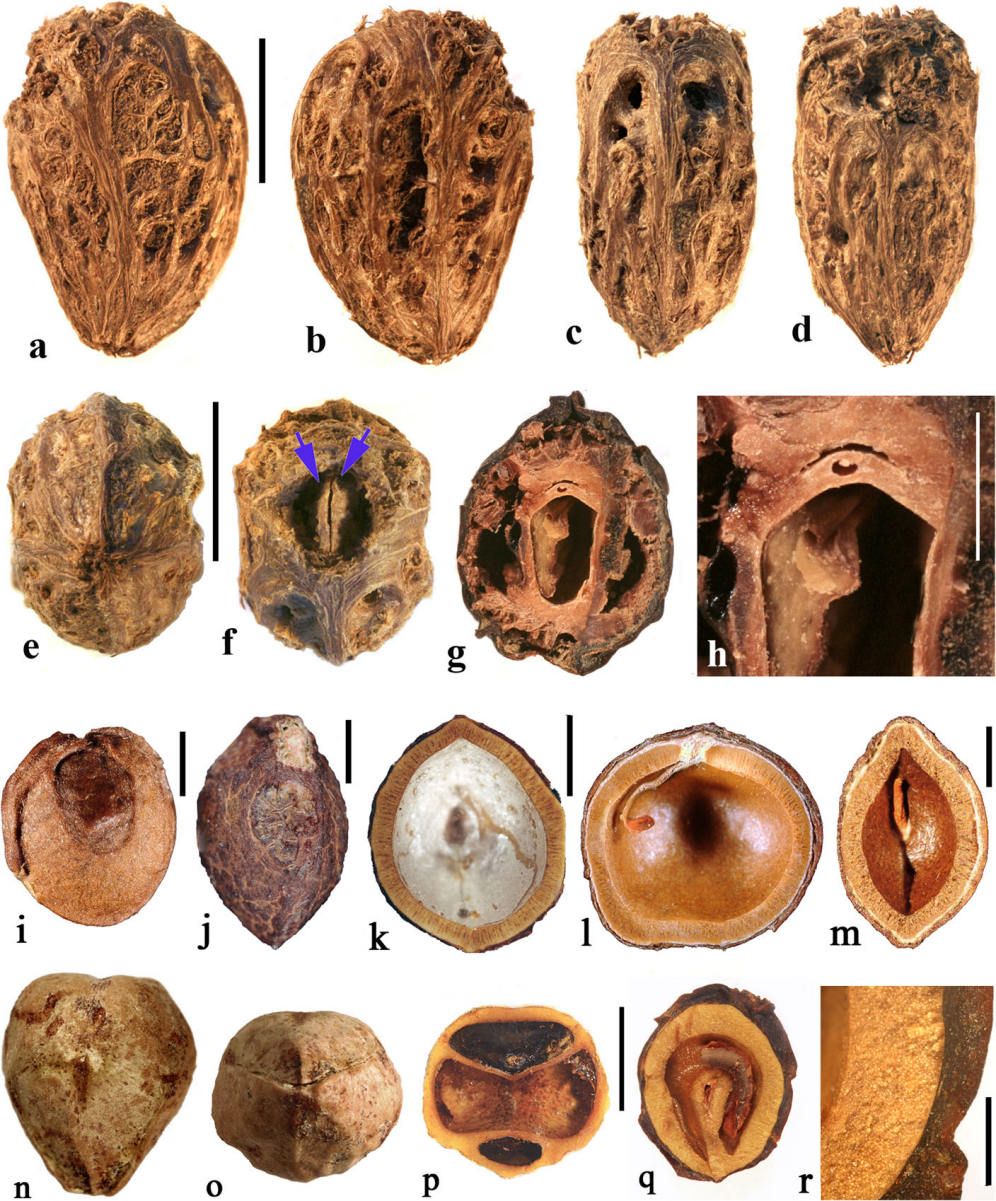
*Attilaea*, *Buchanania,* and *Campnosperma***. a–h** *Attilaea abalak* (K: E. Martínez & D. Alvarez 30,821; Campeche, Mexico). **a–d** Lateral views of the same fruit rotated on its axis, **e** basal view, **f** apical view, note pore with recessed bilabiate internal germination mechanism (arrows). **g** Transverse section, **h** detail of locule area from g. **i–m** *Buchanania* fruits. **i** *B. arborescens.* (NY: C. Niyomdham 1687; Thailand). **j** *B*. *arborescens* (syn.: *florida*) (US 43698: E. D. Merrill 1987; Luzon, Philippines). **k, l** Transverse and sagittal sections of fruit in i. **m** Transverse section of specimen in j. **n–r** *Campnosperma panamense* fruits (US 1317201: G. P. Cooper & G. M. Slater 154; Changuinola Valley, Panama). **n** Endocarp in lateral view. **o,** Same specimen in apical view. **p** Same specimen, transversely sectioned, transecting the single locule twice, on either side of the central lacunae. **q–r** *C*. *micranteium* (P 6634080: R. Capuron 9013; Madagascar). **q** sagittal section showing strongly curved seed. **r** Detail of the pericarp from q; endocarp composed of isodiametric sclereids. Scales bars 5 mm in a (also applies to b**–**d), e (also applies to f, g), and q (also applies to n**–**p); 2 mm in h**–**m; 1 mm in r

The drupe is ellipsoidal, about 15 mm long, and red at maturity, with very acidic taste (Martínez & Ramos, 2007). The stone is prolate, obovoid in lateral view with a keel in the plane of bisymmetry and is essentially unilocular although a collapsed second locule is visible in cross section (Fig. 4g). Endocarp ornamentation includes several prominent longitudinal ribs and intervening broad depressions and reticulate sculpture (Fig. 4a**–**d). Along the dorsal margin near the apex are a few prominent apertures (Fig. 4c). There is a single apical pore, lacking an operculum, but showing a recessed bilabiate germination mechanism (Fig. 4f). A pair of lacunae flanks the locule (Fig. 4g).

The locular envelope of *Attilaea abalak* is 0.1 to 0.2 mm thick, composed of horizontal fibers (Fig. 4h). The rest of the endocarp is composed mainly of isodiametric brachysclereids, with external tracts of fibers that form the sculptural ribs. Except for the reduction in locule number, the fruit morphology and anatomy strongly resembles that of *Spondias*, particularly *S. purpurea* (see below).

### *Buchanania* Spreng. (Fig. 4i–m)

*Buchanania,* originally placed in tribe Anacardieae by Engler (1883), falls between “Spondioideae 1” and “Spondioideae 2” as sister to the former clade in the molecular phylogeny of Weeks et al. (2014). *Buchanania* is a genus of 25–30 species in tropical Asia, Malesia, Australia, Micronesia, Melanesia, and Samoa, of which two were sampled for the present study, not including the type species *B. lanzan* Spreng. Morphologically, it resembles the monotypic genus *Androtium* of the Malay Peninsula and Borneo, which we did not include in this survey. Although *Buchanania* fruits lack specialization in germination mode characteristic of spondioids, the genus has been treated recently as part of the Spondioideae (Pell et al., 2011) and is strongly supported as sister to the clade of Spondioideae 1 in the phylogenetic assessment by Weeks et al. (2014). Flowers of *Buchanania* possess 4 to 6 carpels, but only one is fertile (Pell et al., 2011).

The fruit is obliquely oblong, with a fleshy mesocarp and purple, yellow or orange exocarp. The endocarp is elliptical-obovoid in lateral view (Fig. 4i, j) with a keel in the plane of bisymmetry. It is unilocular, and lacks germination pores, valves, or opercula. The endocarp surface is smooth to minutely reticulate. Transverse sections at the equator of the endocarp show an elliptical locule (Fig. 4k, m) while sagittal sections along the plane of bisymmetry show the locule to be nearly circular in outline (Fig. 4l). Lacunae are lacking.

The endocarp consists of an inner layer about 0.3**–**0.4 mm thick of variously oriented elongate brachysclereids, surrounded by 2–4 layers, collectively 0.2**–**0.3 mm thick, composed of anticlinally oriented elongate brachysclereids (Fig. 4k**–**m). This anatomy resembles that of *Rhus,* with its multiple layers of anticlinal sclereids (Manchester 1994) more than it does fruits of other Spondioideae.

### *Campnosperma* Thwaites (Fig. 4n–r)

*Campnosperma*, originally placed in tribe Rhoeae by Engler (1883), has been inferred as sister to a large clade including both “Spondioideae 1” and subfamily Anacardieae, but with low posterior probability support, in the phylogeny of Weeks et al. (2014). Although its relationship to Spondioideae remains tentative, we review this genus here for comparative studies. The genus includes about 13 species distributed from Honduras to Brazil, and in Madagascar, the Seychelles, Sri Lanka, southeast Asia, Malesia, Micronesia and Melanesia; two species were sampled for this study, not including the type species *C. zeylanicum* Thwaites.

The drupes are subglobose to obovoid and are red to black at maturity. The stone is obovate, approximately circular in cross section, and bisymmetrical and unilocular (Fig. 4n, o). The locule and seed are strongly curved, encircling a central cylindrical lacuna (Fig. 4p, q) with a configuration that is unique in the Anacardiaceae. The endocarp surface varies from smooth to slightly ribbed (Fig. 4n, o). Fruits lack specialization in germination mode. The endocarp wall is uniform, composed of isodiametric sclereids and lacks distinction of a locular envelope (Fig. 4r). Given the highly unusual morphology, lack of a locular envelope, and endocarp composed exclusively of isodiametric sclereids without any fibers, it seems unlikely that *Campnosperma* is correctly placed in the Spondioideae in the traditional sense, or to either of the groups, Spondioideae 1 or 2, discussed by Weeks et al. (2014).

### *Choerospondias* Burtt. & Hill (Fig. 5)

This genus has one extant species, *Choerospondias axillaris*, distributed in northeastern India, Nepal, Bhutan, Myanmar, southeastern China, Japan, Vietnam, and northern Thailand. Fossil fruits of the genus have been recognized from the Eocene London Clay flora (Reid & Chandler, 1933; Chandler, 1961; Manchester et al., 2009), from the Oligocene of China (Fu et al., 2017), and from the early middle Miocene of Poland (Kowalski, 2010); also from Japan (as *Spondias axillaris*) in sediments now considered to be middle Miocene (Tsukagoshi, 2011).

**Fig. 5:**
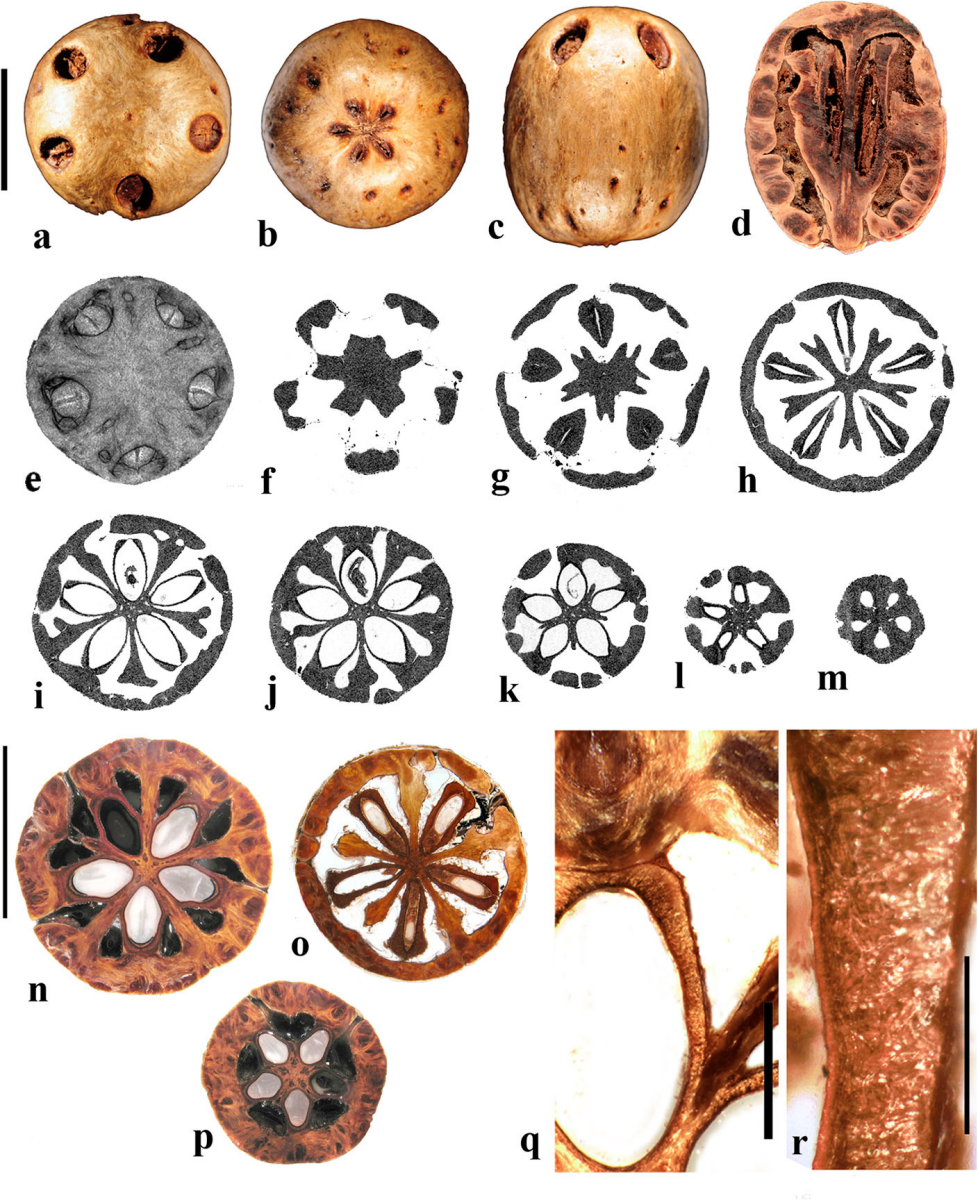
*Choerospondias axillaris*. **a–e** Endocarp (UF1117: Moore et al. sn; Khao Yai National Park entrance, Thailand). **a** Apical view showing five elliptical pores partially covered with parenchymatous tissue. **b** Basal view showing whorl of 5 basal apertures as well as scattered apertures. **c** Lateral view showing prolate shape. **d** Sagittal section showing prominent longitudinal locules and lacunae (UF 1253: Kokawa & Manchester s.n.; Osaka, Japan). **e** Apical view of endocarp: Volume rendering from CT scan data. (UF 2552: S. Manchester s.n.; Yunnan, China). **f–m** Successive transverse sections from apex toward base of a single specimen (UF 2552) showing five apical pores (**f**, **g**), apically thickened locular envelopes (**g**, **h**), and lacunae that are more expanded apically than equatorially or basally and directly linked to the whorl of five basal apertures (**m**). **n** Transverse section near equator, with ten apparent lacunae (two between adjacent locules). Note 5 distinct vascular bundles in central axis. **o** Transverse section below equator with 5 apparent lacunae (one between each of the adjacent locules). **p** Transverse section near apex where the locular envelopes are prominent and the lacunae that appeared distinct in lower sections are coalesced into a large cavity (UF 1117: R. Moore et al. sn; Thailand). **q** Detail of cross section of locule and adjacent lacunae. **r** Anatomy of locular envelope in transverse section. Scale bars 1 cm in a (also applies to b**–**m), and n (also applies to o, p); 2 mm in q; 0.5 mm in r

The drupes are yellow at maturity and the mesocarp is thin and fleshy. The endocarp is prolate, circular in transverse section, and usually pentalocular (Fig. 5a–c, i–p). Occasionally, 3-, 4- and 6-loculed fruits are observed, but regardless of the locule number, the stones retain well developed radial symmetry with no outward indication of abortive locules. The endocarp surface is predominantly smooth with scattered punctae (apertures) (Fig. 5a–c). There is an apical whorl of prominent elliptical germination pores corresponding to the locules (Fig. 5a, c, e) and a basal whorl of apertures alternating in position with the locules (Fig. 5b, m). Typically, all locules are fertile, equally developed, ovate to elliptical in transverse section (Fig. 5f–p), and elongate in longitudinal section (Fig. 5d). The central axis has five (or more or less, depending on the number of locules) distinct vascular strands, one aligned to each of the radiating septa (Fig. 5n, p). Each of the locules terminates beneath an elliptical germination pore. Opercula are lacking, but a membranous layer covers each pore. When this layer is removed, the keeled apical part of the locular envelope is visible (Fig. 5e, h). From this, we infer the germination mechanism to be recessed bilabiate.

Prominent, longitudinally elongate, lacunae are symmetrically arranged about the locules. In transverse sections near the equator, the lacunae appear as ten (twice as many as the locules), arranged in pairs flanking each of the locules and are more or less triangular in this plane of view (Fig. 5i, j, n). Toward the base, the lacunae flanking adjacent locules coalesce, giving the appearance of five cavities (equivalent to the number of locules), alternating with the locules (Fig. 5k, l). Apically, the same lacunae coalesce to form a single common space surrounding the locules, inside the uniformly thick outer wall of the stone (Fig. 5d, g, h, o). Presumably this space near the apex provides room for opening of the locular envelopes at germination. Narrow anticlinal channels extend from the lacunae through the endocarp wall, linked to dimple-like surface apertures (indicated above as punctae) that are scattered over the endocarp surface (Fig. 5d, k, n).

Locular envelopes (Fig. 5q, r) are relatively thick (0.2–0.8 mm), formed by a lining one to a few cells thick of isodiametric sclereids, a thin layer (60 to 100 μm thick) of encircling periclinal fibers (these fibers about 10 μm thick), surrounded by a thick layer (400–700 μm thick) of mostly anticlinal fibers with occasional interspersed sclereids (20 μm in diam.). The surrounding stone is formed mostly by tracts of tortuous fibers, commonly arranged more or less anticlinally (Fig. 5d, n, q).

### *Cyrtocarpa* Kunth (Figs. 6, 7)

As currently circumscribed, this Neotropical genus contains five species; for the current work, we sampled four of them, including the type species, *Cyrtocarpa procera*. A key to four of the species and discussion of distinction from other Spondioideae was provided by Mitchell and Daly (1991) followed by the recognition of an additional species, *C. kruseana* by Fonseca (2005).

**Fig. 6:**
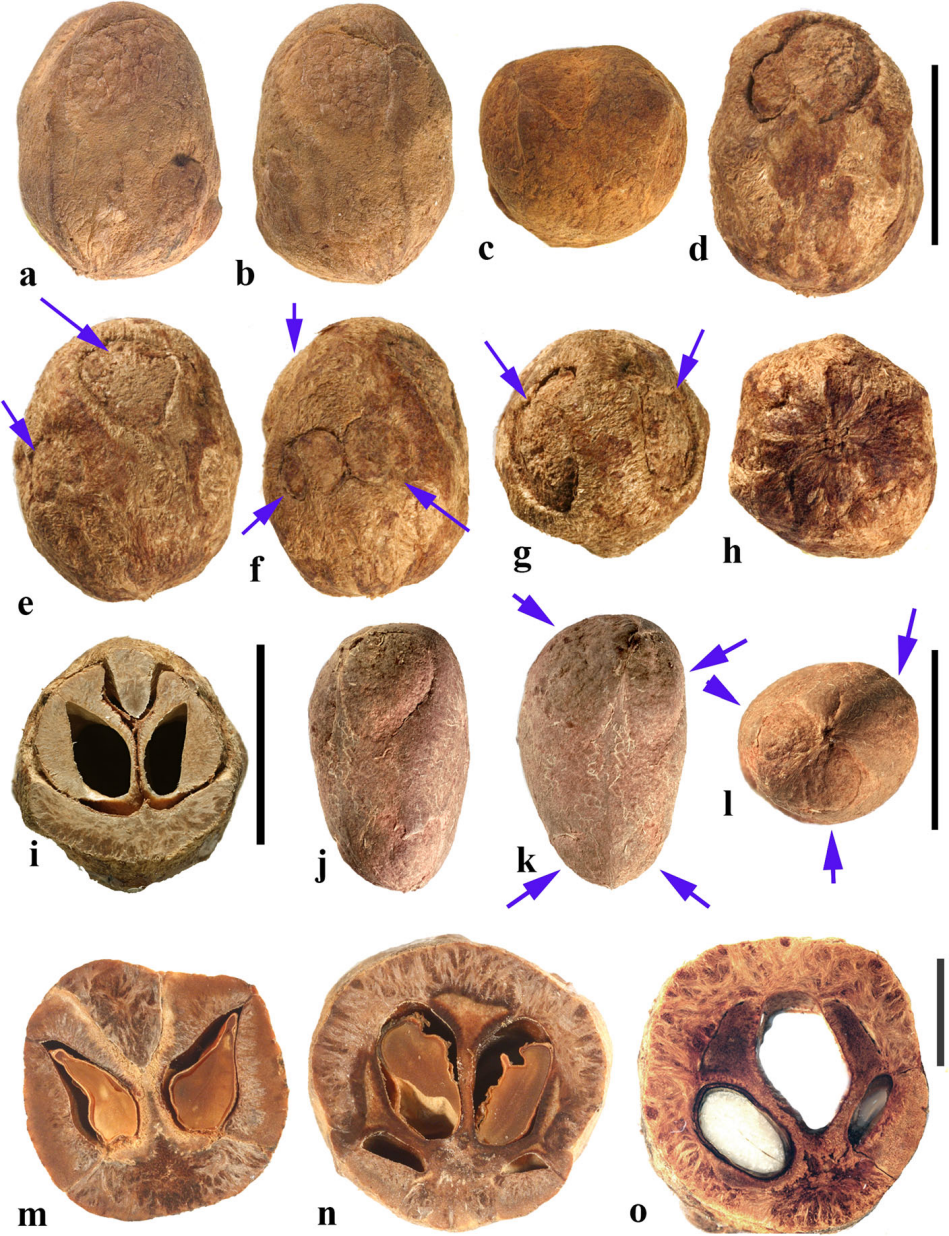
*Cyrtocarpa*. **a–c** *C. procera*, lateral and apical views (MO 3487625: J. Garcia 1013; Guerrero**,** Mexico). **d–i** *C. procera* (UC 125741: C.I. Purpus s.n. coll. June 1908; Santa Lucia, Puebla, Mexico); lateral views in d**–**f, apical and basal views in g and h, showing variously positioned opercula (arrows), equatorial transverse section in i. **j–l** *C. edulis* (K 81604: Carter 2460; Baja California, Mexico); note apical and basal position of opercula (arrows). **m, n** cross sections of specimen in a**–**c. **o** *C. procera* (US 1269540: B.P. Reko 4967; Achotla, Guerrero, Mexico). Scale bars = 1 cm in a**–**l; 5 mm in m**–**o

**Fig. 7:**
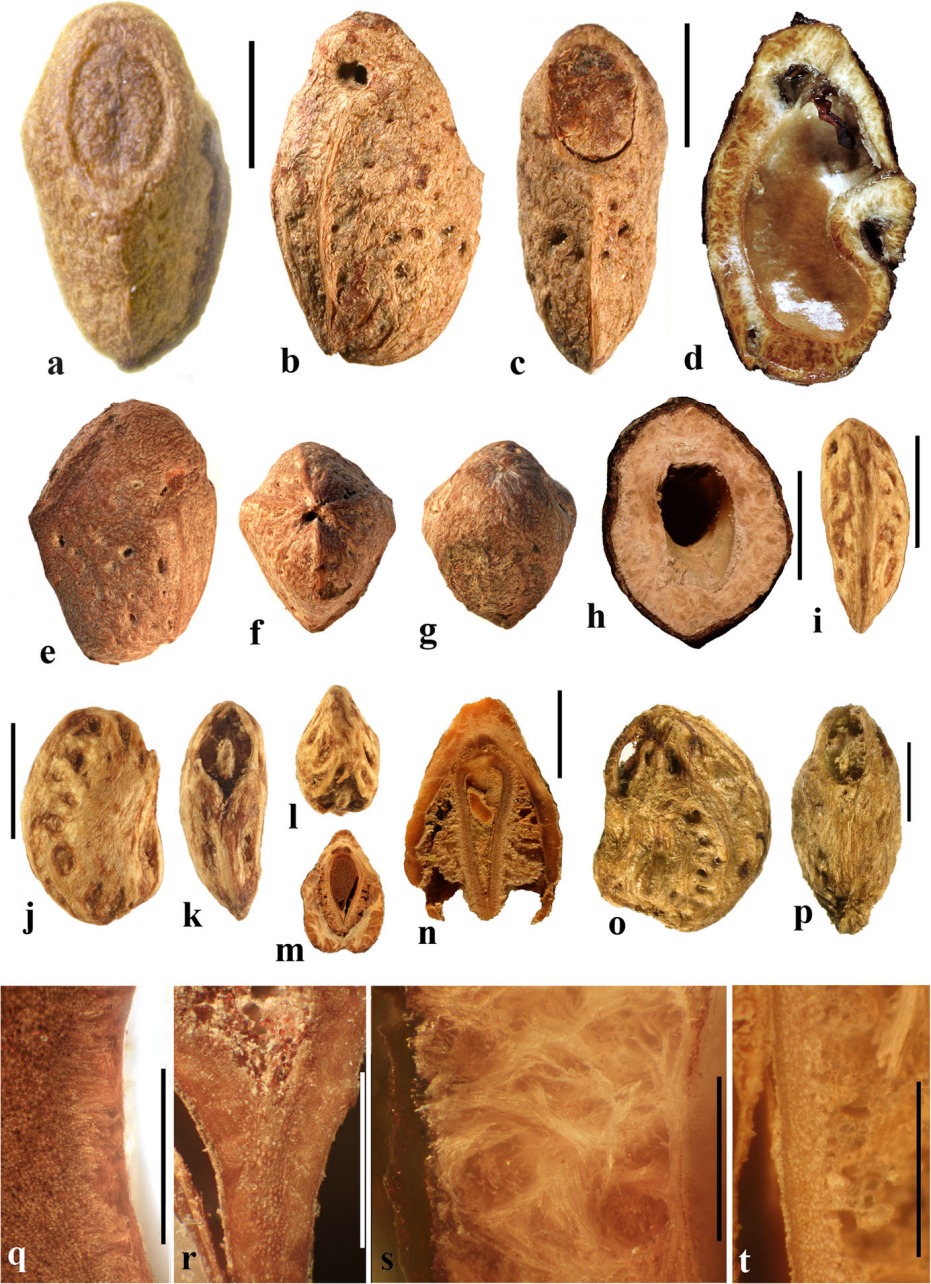
*Cyrtocarpa* continued. **a–h** *C. velutinifolia*. **a** Frontal view of stone, note single apical operculum (K: M.J. Jansen-Jacobs et al. 2120; Rupununi Distr., Guyana). **b–d** Lateral, frontal views and sagittal section; note plug-like operculum seen in d (US 3344305: T.W. Henckel 3562; Guyana). **e–g** Lateral, basal, and apical views of *C. velutinifolia* (K: Hoffman 1071; Guyana). **h** Another specimen from same collection as b-d, transverse section of fruit with exocarp remaining. **i–l** *C. caatingae* endocarp in dorsal, lateral, ventral and apical views (NY: Carvalho et al. 3762; Brazil). **m** Same in equatorial transverse section. **n** Same in transverse section near apex, showing pore opening at base of image, locular envelope and prominent lacunae. **o, p** *C. caatingae* endocarp in lateral and ventral views (US 3396073: D. Alvarenga et al. 1291; Goias, Brazil). **q–t** Anatomical details of locule areas of *Cyrtocarpa* specimens. **q, r** *Cyrtocarpa procera* (US 1269540: B.P. Reko 4967; Mexico). **s** *C. edulis* (UC 703692: J.T. Howell 10,616; Mexico)*.* **t** *C. caatingae*, detail from n. Scale bars = 5 mm in a, d, h (also applies to b, c, e**–**g), i and j (also applies to k**–**m); 2 mm in n; 5 mm in p (also applies to o); 1 mm in q**–**t

Drupes are purple or yellow to orange with fleshy mesocarp. Most species, including *Cyrtocarpa procera, C. edulis,* and *C. kruseana,* have endocarps that are prolate to obovoid, with a smooth surface lacking punctae (Fig. 6a–h, j, k), and 3**–**4 (5) irregularly positioned, elliptical simple opercula (stopper-like plugs). The opercula can be located apically (Fig. 6a, d, e, g, j), equatorially (Fig. 6f), or basally (Fig. 6h, k, l), and are commonly irregular in size and shape. This condition appears to be unique in Spondioideae. In the stones of other operculate genera (e.g., *Antrocaryon, Operculicarya, Poupartia,* and *Sclerocarya*), the opercula are generally confined to the apical portion. In *Cyrtocarpa*, the central axis of the stone is eccentric and the locules unevenly developed and of various shapes in transverse section (Fig. 6i, m–o). Lacunae are usually present between adjacent locules. A few small basal depressions can be present adjacent to the point of fruit attachment. Locular envelopes of *C. procera* (Fig. 7q, r) and *C. edulis* (Fig. 7s) are about 0.2**–**0.3 mm thick and poorly defined at the outer margin, with anticlinal fibers grading to the surrounding endocarp tissue of isodiametric brachysclereids. The outer wall of the stone is composed mostly of variously oriented tortuous tracts of fibers (Fig. 6m**–**o). The operculum is formed of cells similar to and continuous with those of the other parts of the outer endocarp wall.

The specimens we examined of *Cyrtocarpa velutinifolia* (Fig. 7a**–**h) are distinct from the *Cyrtocarpa* species described above in having only one developed locule, and are bisymmetrical with a keel in the plane of symmetry (Fig. 7c). The endocarp is almost smooth, but not so smooth as the species described above, to somewhat ribbed, and has scattered depressions on the surface (Fig. 7b, c, e). A simple, elliptical operculum (Fig. 7a, c**–**e) is on the ventral side of the stone, bisected by the plane of fruit bisymmetry (Fig. 7d). Contrasting with *C. procera* and *C. edulis*, the locular envelope is thin (<0.1 mm) and composed of dense narrow fibers 5**–**8 cells thick that are oriented horizontally and periclinal to the locule (Fig. 7s). The main layer of the endocarp surrounds the envelope and is 1.2**–**1.5 mm thick, composed of variously oriented tracts of tortuous fibers. The operculum is comprised of the same tissue of tortuous fibers as the rest of the endocarp. Lacunae are not obvious. The combined mesocarp and exocarp in dried specimens is about 0.3 mm thick. Differences among these fruits and those of the type species, *C. procera* and other species of *Cyrtocarpa* suggest that *C. velutinifolia* may represent a distinct genus.

*Cyrtocarpa caatingae* (Fig. 7i**–**p, t) has unilocular endocarps similar in shape and bisymmetry to those of C. *velutinifolia* but is readily distinguished from the other species of this genus by the lack of opercula*.* Instead, there is an elliptical apical pore with the keel of locular envelope recessed beneath. The endocarp surface in this species is reticulately ribbed, with concavities between some of the ribs. The locular envelope is 0.2**–**0.3 mm thick, composed exclusively of isodiametric sclereids (20**–**40 μm diam.), and is laterally flanked by prominent parenchyma-filled lacunae (Fig. 7n). The endocarp wall is composed of tortuous fibers (Fig. 7t).

In summary, we observed three categories of fruit morphology within *Cyrtocarpa* as the genus is currently treated. The first category (including the type species, *C. procera*) has smooth, multilocular endocarps with opercula in various positions, basal to apical. The second category, *C. velutinifolia*, differs in its sculptured endocarp surface and is unilocular with a single apical operculum. The third category, *C. caatingae*, is also unilocular and sculptured, but differs from both other kinds by the germination mechanism, which does not include an operculum. These observations suggest that more than one genus is represented. The two species so far included in molecular studies, *C. procera* and *C. edulis,* shown to form a strongly supported sister group (Weeks et al., 2014), both have the first kind of fruit morphology. It will be interesting to learn from additional investigations where *C. velutinifolia* and *C. caatingae* reside in the overall phylogeny.

### *Dracontomelon* Blume (Figs. 8, 9)

About eight extant species of *Dracontomelon* are presently accepted. The genus is distributed from India to Myanmar, Indo-China, tropical China, Malesia, and Fiji (Pell et al., 2011). *Dracontomelon* was established by Blume (1850) but a type species was not designated when he presented the binomials *D. costatum, D. cuspidatum, D. mangiferum,* and *D. sylvestre.* We studied fruits of *D. costatum, D. dao, D. duperreanum,* and *D. macrocarpum.* Fossil fruits of *Dracontomelon* have been recognized from the early Eocene London Clay flora (Reid & Chandler, 1933). We agree that the genus is represented there, but some nomenclatural problems for those fossils still need to be addressed. The fruits that Reid and Chandler (1933) placed in this modern genus differ from the modern species in opercular morphology and were transferred to an extinct genus by Manchester (1994). However, some of the specimens that were attributed by Reid and Chandler to *Pseudosclerocarya*, appear to actually represent *Dracontomelon* (Collinson & Manchester, work in progress). *Dracontomelon* has also been identified based on well-preserved fruits from the late Eocene of Panama (Herrera et al., 2012).

**Fig. 8:**
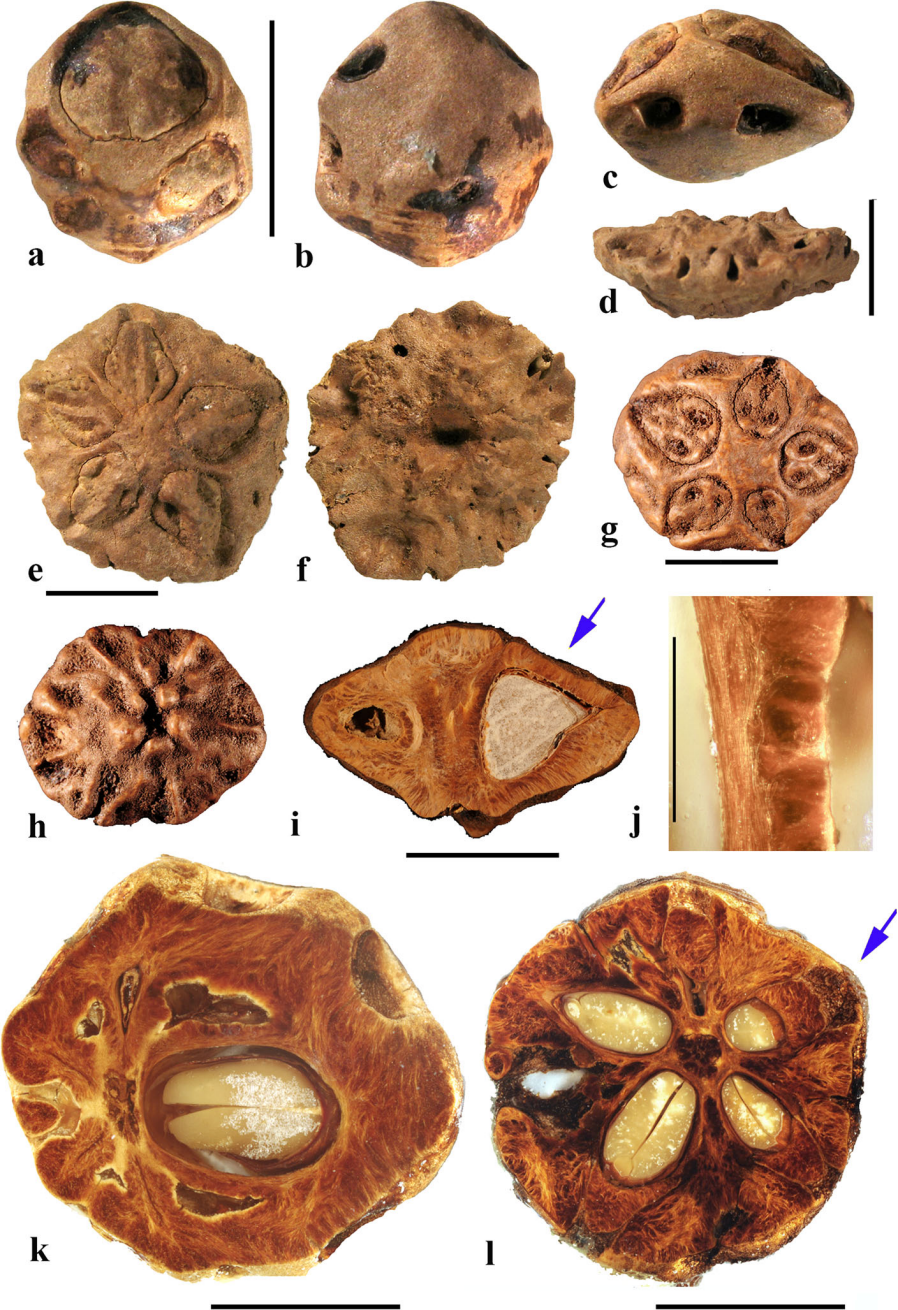
*Dracontomelon dao*. **a–c** Apical, basal and lateral views (NY: Soejarto et al. 7822; Luzon, Philippines). **d–f** Lateral, apical and basal views (NY: Elmer 8307; Luzon, Philippines). **g, h** Apical and basal views (NY: M. Ramos Edano 75,781; Catanuones, Philippines). **i** Sagittal section of the same fruit as g, h**,** showing operculum (arrow). **j** Detail of locular envelope from fruit in a**–**c. **k** Transverse section of *D. dao* from a. **l** Transverse section from g: note pair of channels connecting equatorial external apertures with lacunae (arrow). Scale bar 1 cm in a (also applies also to b, c), d, e (also applies to f), g (also applies to h, i), and l; 1 mm in j; 5 mm in k

**Fig. 9:**
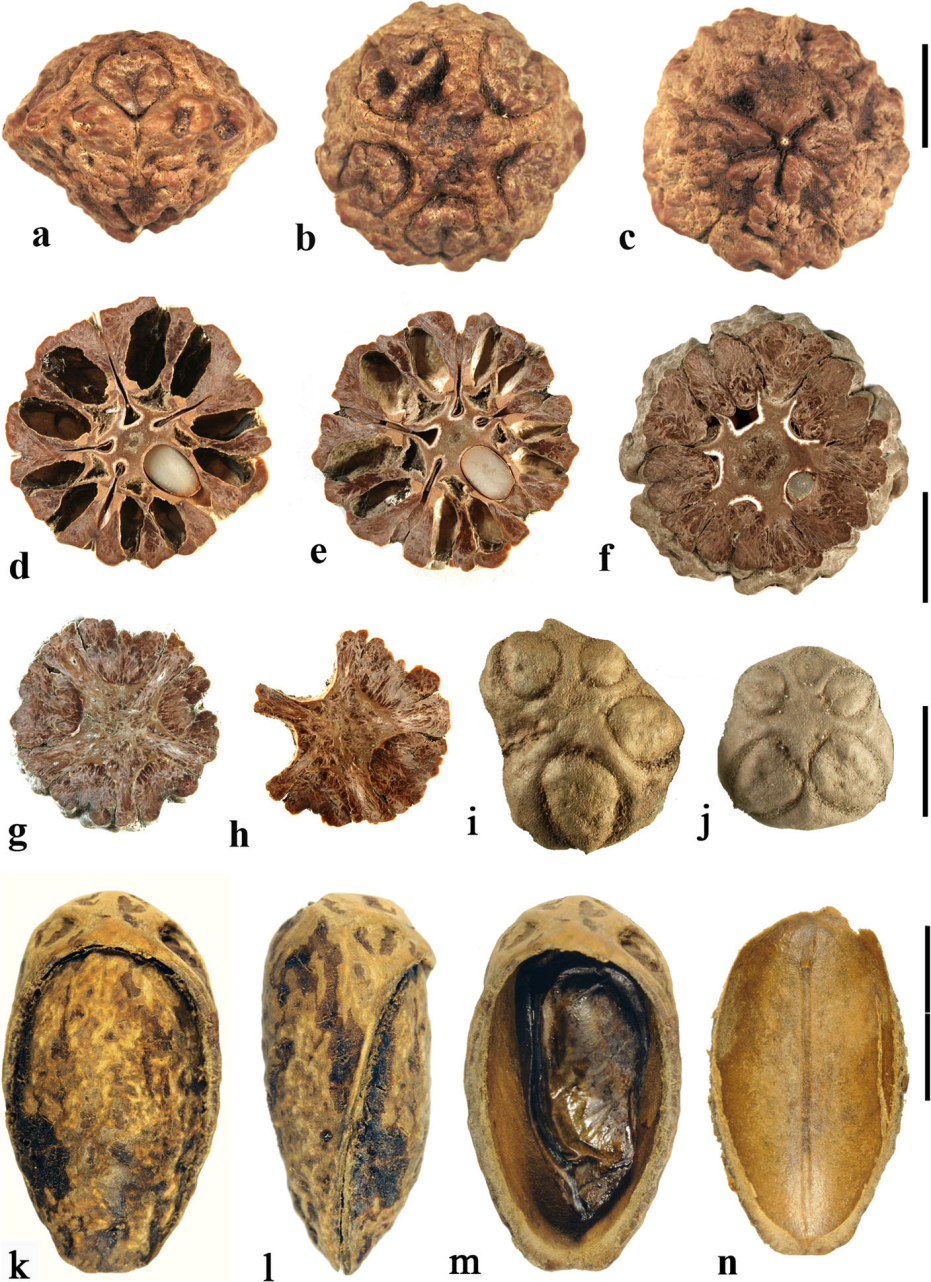
*Dracontomelon,* continued. **a–h** *D. macrocarpum* (UF 2546: Zhou Zhekun s.n.; Yunnan, China). **a–c** Lateral, apical, and basal views. **d–h** Transverse sections. **i, j** *D. duperreanum* (UF 2497: Xiaoyan Liu s.n.; Guangzhou Botanical Garden, Guangdong, China). **k–n** *D*. *costatum* (US 3006219: A. Kostermans 13,229; East Borneo). **k** Lateral view of endocarp, showing single well developed operculum (four abortive). **l** Same specimen, turned 90 degrees. **m** same view as in k with operculum removed; note single locule. **n** Ventral side of operculum; note internal keel. Scale bars 1 cm in c (applies also to a, b), f (applies also to d, e), j (applies also to g**–**i), and n (also applies to k**–**m)

The exocarp of *Dracontomelon* is brown to black and the mesocarp is thin and fleshy. The endocarp surface varies from smooth (Figs. 8a**–**c and 9i, j) to conspicuously ridged or verrucate (Figs. 8e–h and 9a**–**c). This genus is variable in shape, size and symmetry of the endocarp reflecting the number of locules that are developed. The usual number of carpels is five, but their development is often uneven. When five locules are more or less equally developed, then the fruit and stone are oblate to subglobose (Fig. 8a**–**i). Often two or three locules become enlarged while others remain small (Figs. 8k and 9i, j). *Dracontomelon costatum* Blume is exceptional in having only one fully developed locule (four abortive) and a prolate fruit (Wilkinson 1968; Fig. 9k**–**n), whereas fruits in the other species are typically pentalocular and oblate. Equatorial transverse sections show pentagonal to circular endocarps (Figs. 8k, l and 9d). The locules are predominantly elliptical in outline. Longitudinal sections of the drupes show that the long axes of the locules as viewed in sagittal section are reniform (Fig. 8i).

Each locule has an elliptical apical simple operculum (stopper-like plug, Figs. 8a**–**e, g and 9a, b, i). Lacunae alternate with the locules at the equator and are connected by a pair of channels to equatorial external apertures. Thus, in pentalocular fruits there are 10 regularly spaced external equatorial apertures (Figs. 8c, d and 9a). The lacunae are irregularly- to almost triangularly-shaped in transverse section and sometimes appear filled with fibers (Figs. 8k, l and 9d, e). The outer surface of the opercula in *D*. *dao* is ornamented with 2 or 3 symmetrically placed elliptical depressions (Fig. 8e, g). No apertures are observed at the base of the endocarp, but sometimes a deep and single depression can be seen at the point of fruit attachment (Figs. 8f, h and 9c).

The locular envelope is about 0.3 mm thick and 6**–**10 seriate, consisting mainly of periclinal fibers (Fig. 8j). The surrounding portion is formed by variously oriented tracts of tortuous fibers, which are mostly anticlinal in the peripheral parts of the fruit. The same tissue of mostly anticlinal tortuous fibers forms the opercula (Fig. 8i, l).

### *Haematostaphis* Hook. f. (Fig. 10)

*Haematostaphis* is a monotypic genus; *H. barteri* occurs from Tropical West Africa east to South Sudan. The flowers show 3 carpels, 3 styles and 3 stigmas (Mitchell et al., 2006; Pell et al., 2011).

**Fig. 10:**
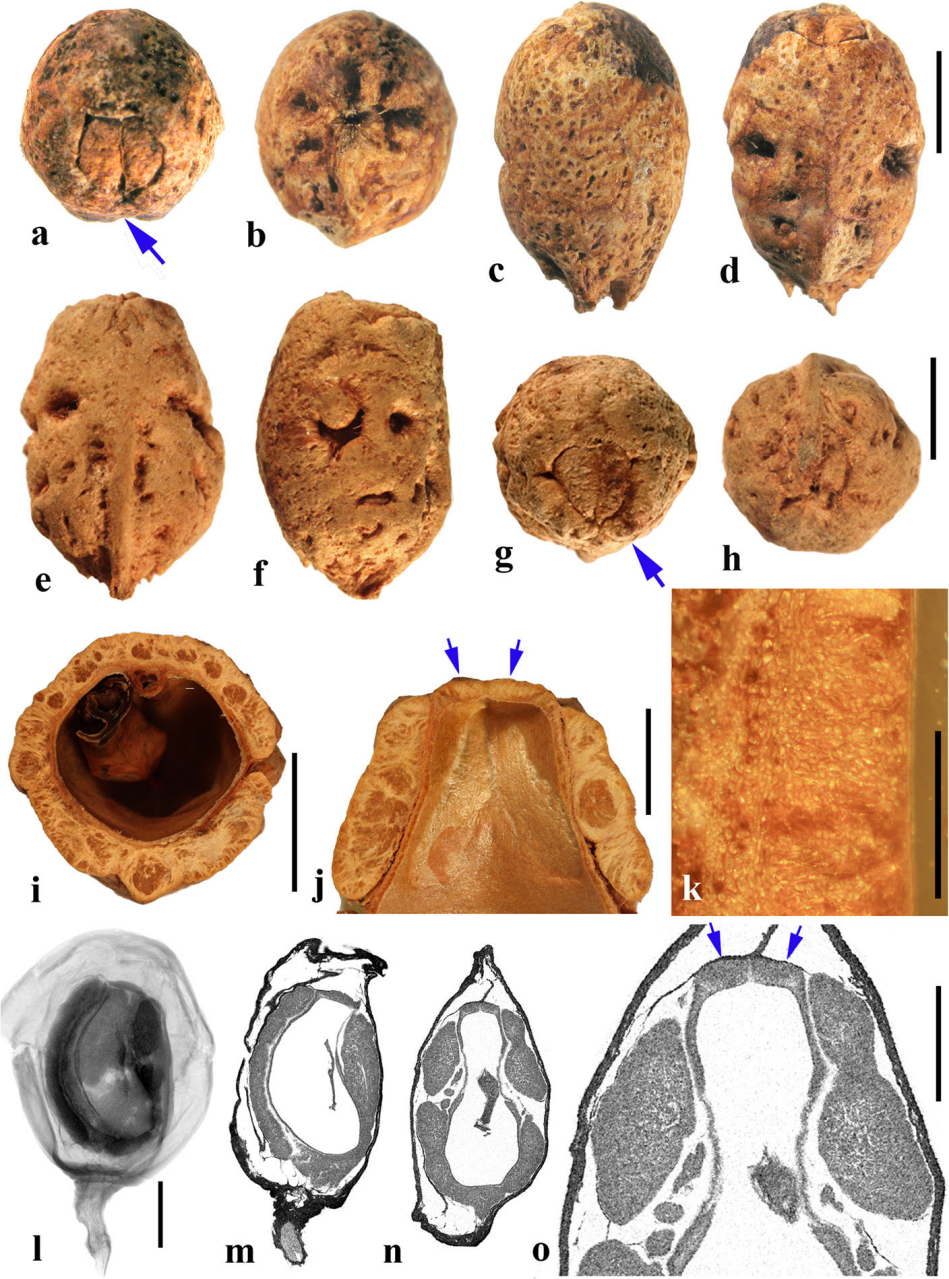
*Haematostaphis barteri*. **a–d** Endocarp in apical view showing bipartite operculum (arrow), basal, lateral and front views (Kew H 2001/02777 15/34: N.C. McLeod 840; Gold Coast, Africa). **e–h** Endocarp in frontal, lateral, apical and basal views (MO 2284758: Latilo, Okeke and Odewo 69,351; Kwara, Nigeria); note bipartite operculum (arrow). **i** Transverse section of the stone in e–h. **j** Coronal section through the operculum of specimen in g, showing bipartite construction (arrows indicate two “shutters”). **k** Detail of locular envelope. **l** Lateral view of fruit from CT-scan data with mesocarp and exocarp rendered translucent, revealing unilocular endocarp (McLeod 840). **m, n** Same specimen in sagittal and coronal digital sections. **o** Enlargement of coronal section showing median split line of the bipartite operculum and two “shutters” (arrows). Scale bars 5 mm in d, h, i (also applies to a**–**c, e**–**g), l (applies also to m, n); 3 mm in j, o; 1 mm in k

The mesocarp is thin and fleshy and the exocarp is red. The endocarp is prolate, obtuse-pointed basally and rounded apically, with a keel in the plane of bisymmetry (Fig. 10a**–**h). The endocarps are circular to lenticular in equatorial transverse section (Fig. 10i). The surface of the endocarp is irregularly pitted and finely punctate (Fig. 10a**–**h) with a few apertures that penetrate to the locule (Fig. 10d, i). Sagittal and coronal sections of the drupes show that the long axis of the locule is straight and has a phalloid distal portion (Fig. 10j, l**–**n). The specimens we studied are unilocular with a single bipartite (shutter-like), apical operculum (Fig. 10a, g, j). The operculum is oval to ovate in outline, equivalent in thickness to the endocarp wall, and bisected by the fruit’s plane of bisymmetry (Fig. 10a, g, j, o). On germination, the operculum separates into two equal halves (i.e., bipartite; Fig. 10a, g, j, o) that are pushed apart by the radicle as it emerges (Hill, 1933). Lacunae were not observed in the endocarp wall. Small basal depressions can be present around the point of fruit attachment and usually vary in number and shape (Fig. 10b, h).

The locular envelope is thick (about 0.25 mm), composed of periclinal horizontal fibers. The endocarp wall is composed of variously oriented tracts of tortuous fibers (Fig. 10i**–**k).

### *Haplospondias* Kosterm. (Fig. 11)

*Haplospondias* is represented by a single species, *H*. *brandisiana* (Kurz) Kosterm. [=*Haplospondias haplophylla* (Airy Shaw & Forman) Kosterm.; syn. *Spondias haplophylla* Airy Shaw and Forman] which occurs in China (Yunnan), Vietnam, Myanmar, and NE India (Assam). It has simple or unifoliolate leaves, contrasting with the pinnately compound leaves of most Spondioideae. Other examples of unifoliolate leaves occur in some species of *Lannea* and *Sclerocarya*. Flowers have a single carpel with a very thick style. Very few collections of this species are known, and until now its fruits have been indicated as “not seen.”

**Fig. 11:**
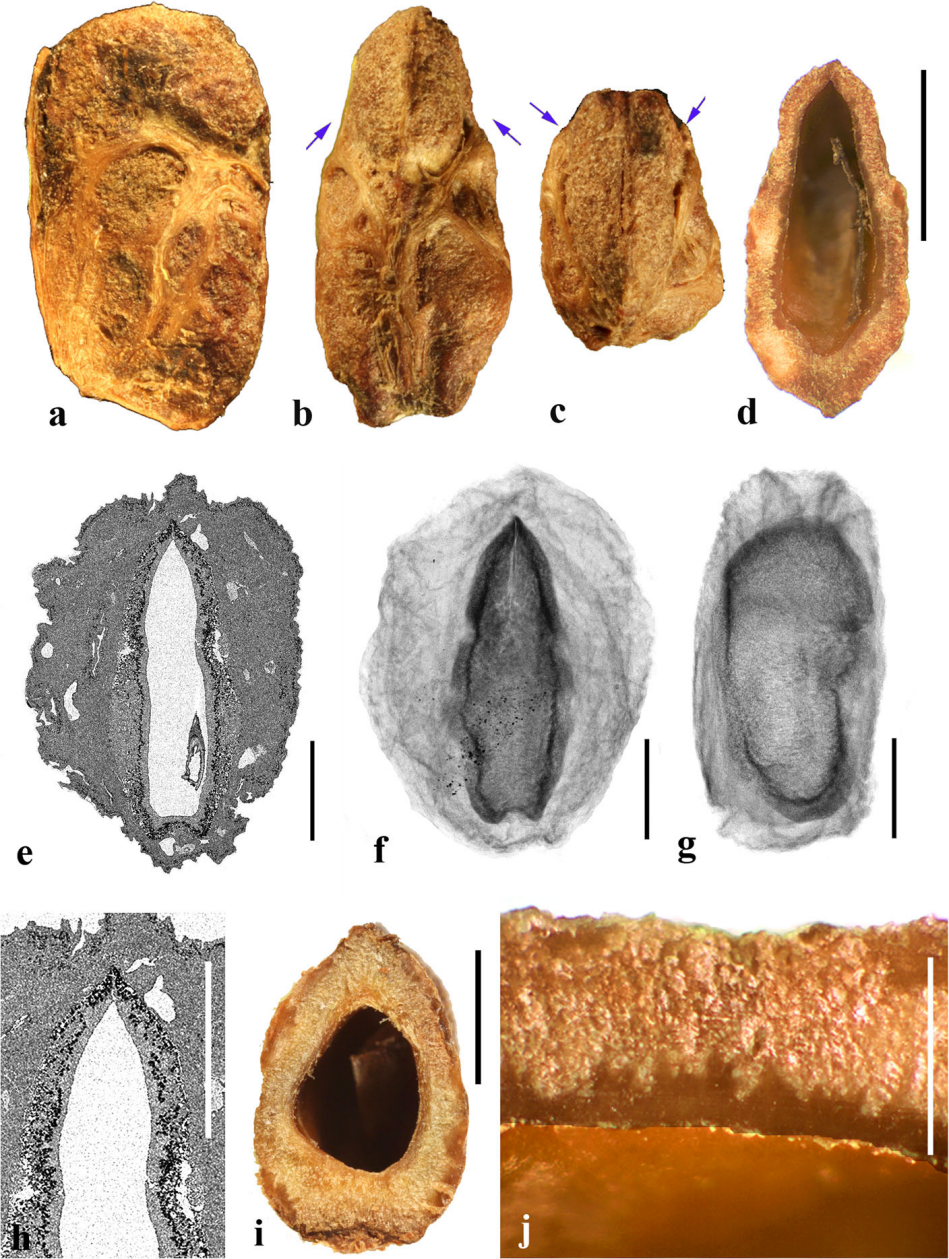
*Haplospondias brandisiana* (XTBG: Jianwu Li 847; Yunnan, China). **a** Endocarp lateral view showing irregular ribs over the lower two thirds of the stone and a smooth, reniform external germination valve. **b** Frontal view showing the keeled apical edge where the germination flaps meet (arrows). **c** Apical view; note external flaps (arrows). **d** Sagittal section of endocarp. **e** Digital sagittal section of fruit from CT scan data showing thick mesocarp. **f, g** Coronal and sagittal volume renderings of fruit. **h** Enlargement from e, showing apically keeled endocarp with vertical line representing separation plane of the apical flaps. **i** Transverse section of endocarp. **j** Anatomical detail of locular envelope and endocarp wall. Scale bars 5 mm in d (also applies to a**–**c); 3 mm in e, h; 2 mm in f, g, i; 0.5 mm in j

*Haplospondias* fruits are ellipsoidal and small (ca. 7 mm long, 5 mm wide) with a fleshy mesocarp (Fig. 11e**–**g) and dark exocarp. The endocarps are unilocular and are laterally compressed with a keel in the plane of bisymmetry (Fig. 11a, b). In equatorial transverse section the endocarps are lenticular (Fig. 11i). The endocarp surface is reticulate ribbed (Fig. 11a, b) except in the apical 1/4, which bears a pair of smooth, broadly elliptical to reniform germination valves (Fig. 11b, c, g). These external valves are oriented parallel to the plane of bisymmetry, extend the full width of the fruit, and appear to split apart from each other along the apical keel (Fig. 11b, c). A prominent longitudinal rib of fibers extends from one of the lateral margins and along the apical keel. Lacunae and basal depressions are absent.

The locular envelope is 0.1 mm thick, and composed of horizontal periclinal fibers about 10 μm in diameter (Fig. 11j). Surrounding tissue of the stone is composed of a 0.4-mm thick layer of isodiametric sclereids (each about 20 μm in diameter). Peripheral strands of fibers contribute to the reticulate surface ribbing of the stone (Fig. 11a). In most features, these fruits resemble those of *Solenocarpus.*

### *Harpephyllum* Bernh. ex Krauss (Fig. 12)

This genus, with a single species, *Harpephyllum caffrum,* is native to the Cape Province, Transvaal and Natal, South Africa. It is a dioecious evergreen tree up to 30 m high (Von Teichman & Van Wyk, 1988).

**Fig. 12:**
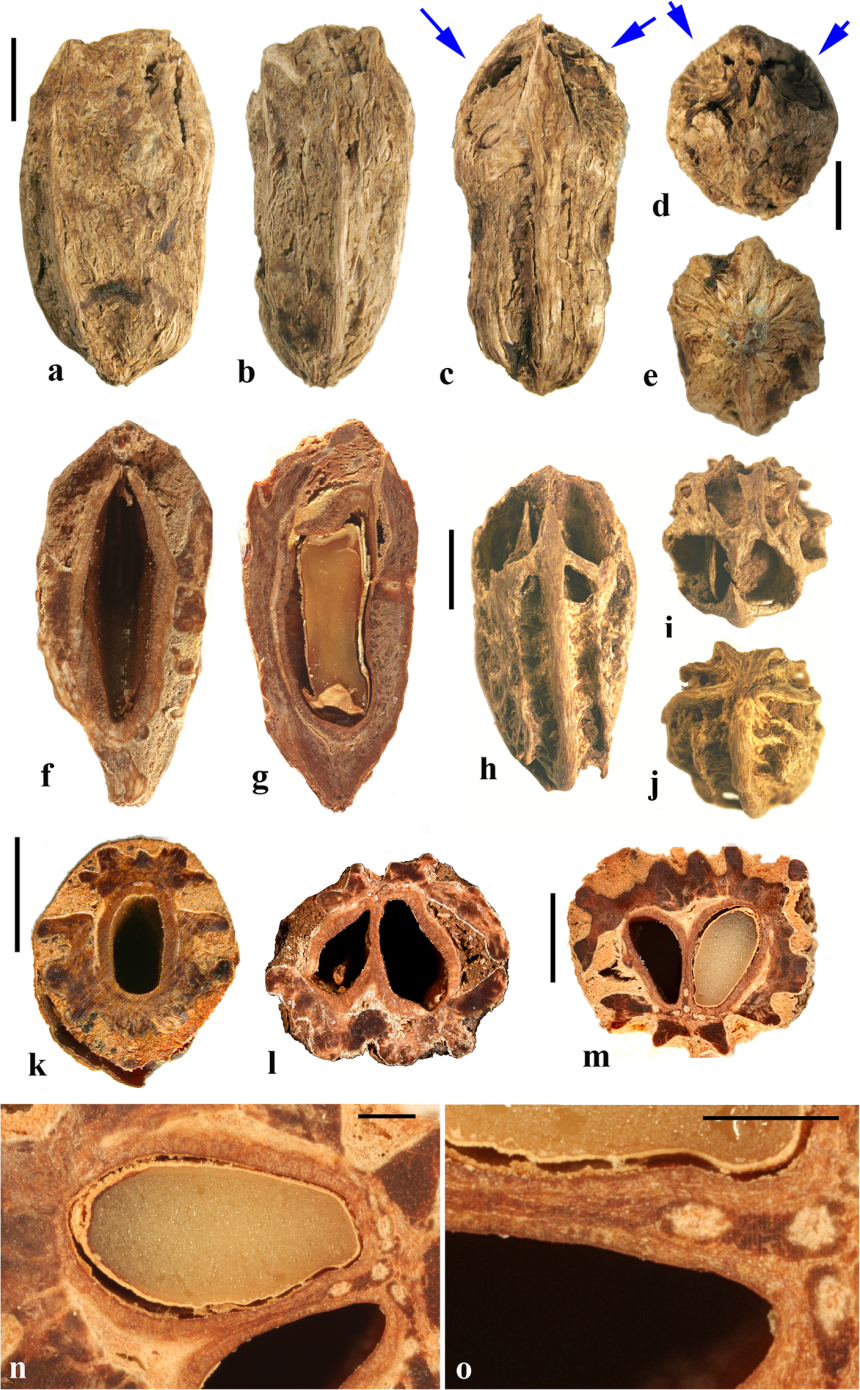
*Harpephyllum caffrum.* **a–e** Lateral, frontal, apical and basal views showing apical pores (arrows) with filling of soft tissue in c, d (NY: Clemens 258; cult, San Diego, California). **f** Coronal section showing apical slit in the locular envelope (MO 5655228 A. Booi 188; Grahamstown, Eastern Cape, South Africa). **g** Sagittal section (MO 1606941: J.F. Stebbins 133; Wellington, South Africa). **h–j** Frontal, apical, and basal views of eroded fruit with endocarp skeleton (Kew carpological collection 6247; Pretoria, S. Africa). **k–m** Transverse sections of stones. **k** Unilocular stone (BARC 162, SPI 18474: C.P. Lounsbury; Cape Town, South Africa). **l** Bilocular stone (NY: J. Lau 1588; cult. Hawaii). **m** Bilocular stone, same collection as f. **n, o** Successive enlargements of **m**, showing locular envelopes. Scale bars 5 mm in a (also applies to b, c), d (also applies to e**–**g), h (also applies to i, j), k and m (also applies to l); 1 mm in n, o

The drupes are about 2.5 cm long, 1.2**–**1.5 mm in diameter, and are unilocular or bilocular. The exocarp is red when ripe. The endocarp is oblong-obovoid; asymmetrical, but nearly radially symmetrical to nearly bisymmetrical, with a dorsal keel (Fig. 12a**–**c, h–j), and is roughly elliptical to circular in transverse section (Fig. 12k–m). The endocarp surface is reticulate and conspicuously and longitudinally ribbed (Fig. 12a**–**e, h**–**j). The ribs are extensions of the bony part of the endocarp, composed mainly of fibers in dense bundles along with brachysclereids; between the ribs are large areas of spongy tissue (Fig. 12f, k, m). Locules are more or less elliptical in cross section (Fig. 12k**–**m) and elongate, only slightly curved in sagittal section (Fig. 12g). Coronal sections (Fig. 12f) reveal an apical keel on the locular envelope that splits open as a pair of internal valves at germination. We did not observe an operculum. Von Teichman and Van Wyk (1988) reported in the same species a woody, bipartite operculum within an apical cavity of the endocarp concealed by spongy tissue of the endocarp, but we consider this to be the apical slit of the locular envelope, unlike the bipartite plug-like opercula of *Haematostaphis* and *Pseudospondias.*

The locular envelope is relatively thick, about 0.5**–**1.0 mm, composed of a mixture of anticlinal fibers and isodiametric brachysclereids (Fig. 12n, o). The remainder of endocarp is composed mainly of tortuous tracts of fibers. Outside the endocarp, the soft tissue of the mesocarp is formed of isodiametric parenchyma. Irregular parenchymatous lacunae, not symmetrically arranged, occur in places between adjacent locular envelopes and the main endocarp tissue (Fig. 12k**–**o).

### *Koordersiodendron* Engler (Fig. 13)

This genus with a single species, *Koordersiodendron pinnatum,* is distributed in the Philippines, Borneo, Sulawesi, Maluku, and western New Guinea. *Koordersiodendron* was included in the Spondioideae by Mitchell et al. (2006) and Pell et al. (2011), but it was not sampled by Weeks et al. (2014). The fruits lack germination pores and opercula typical for Spondioideae.

**Fig. 13:**
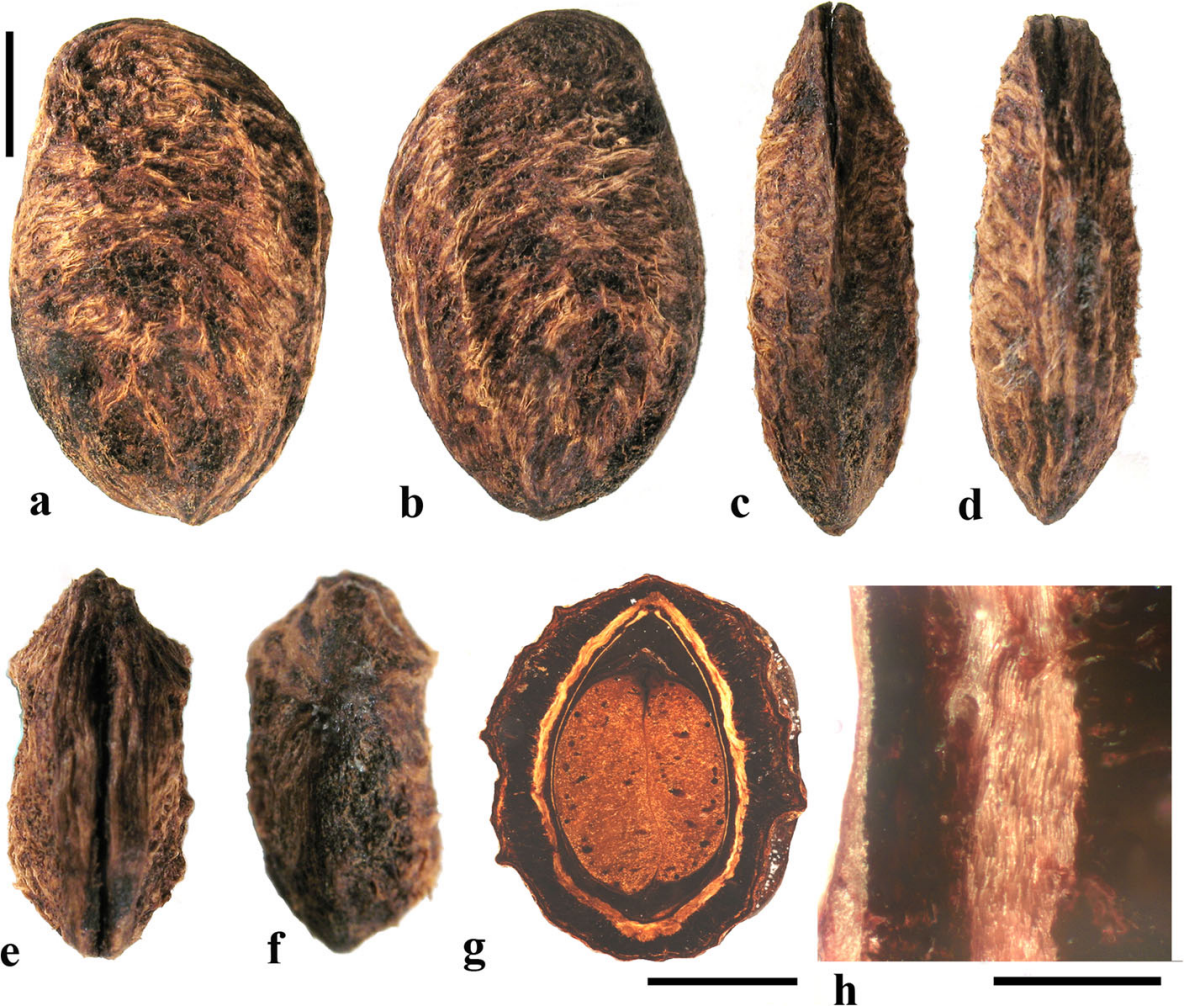
*Koordersiodendron pinnatum.* **a–f** lateral (left, right), frontal, dorsal, apical and basal views (NY: D.D. Soejarto et al. 6571; Palawan, Philippines). **g** Same specimen, transverse section showing single locule, with seed having two semicircular cotyledons, surrounded by thin-walled fibrous endocarp. **h** Enlargement of pericarp, showing thin locular envelope, and thicker fibrous portion of endocarp. Scale bars = 5 mm in a (also applies to b**–**f), and g; 0.5 mm in h

Drupes of *Koordersiodendron* are broadly ellipsoid, oblique at base, and yellow when ripe. The endocarp is unilocular, laterally compressed (Fig. 13c–f), lenticular in transverse section (Fig. 13g), rounded-rhomboidal in lateral view (Fig. 13a, b), keeled in the plane of bisymmetry (Fig. 13d), and has a groove in the same plane over the apical end (Fig. 13e). The endocarp surface is finely striate (Fig. 13a, b). An apical slit that extends up to ~1/3 of the total length in the plane of bisymmetry appears to function in germination (Fig. 13c, e). Lacunae are absent.

The thin locular envelope (ca. 0.07 mm) is composed of a single inner layer of encircling fibers, surrounded by a uni- to biseriate layer of slightly anticlinally elongate sclereids (Fig. 13g, h). Surrounding the locular envelope is a layer of red colored cells, in turn surrounded by a layer of fibers (Fig. 13g, h). The endocarp is composed mainly of horizontally encircling fibers forming a layer ca. 0.5 mm thick, enveloped by a mesocarp ca. 2 mm thick (Fig. 13g, h).

### *Lannea* A. Rich. (Figs. 14, 15)

This genus of sub-Saharan Africa, South and Southeast Asia, and Socotra has about 40 species. We studied fruits of seven species, including the type species, *L. velutina* (Table 1). Weeks et al. (2014) included four species of this genus in their analysis and found them to constitute a monophyletic group with three African species (*L. schweinfurthii, L. rivae*, and *L. welwitschii*) forming a clade sister to the Asian *L. coromandelica*. Acceptable fossil fruit records of this genus occur in the Eocene of Germany (*L*. *hessenensis* Collinson et al., 2012) and Miocene of Kenya (as *Odina miocenica*, Chesters, 1957). The genus was also reported from the London Clay (Reid & Chandler, 1933) but CT scanning results indicate they may represent another genus of Anacardiaceae (Collinson & Manchester, in progress).

**Fig. 14:**
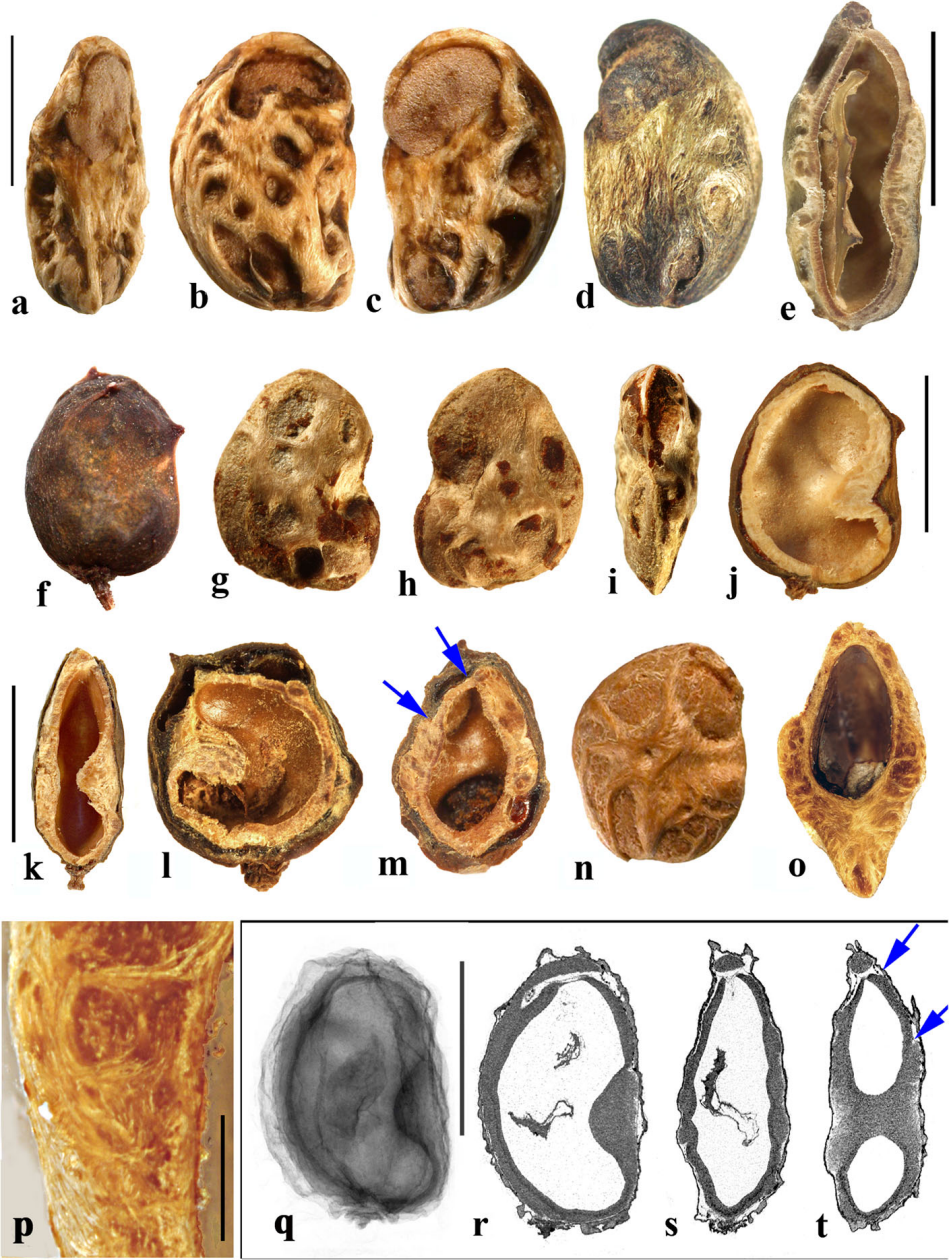
*Lannea*. **a–c** Ventral and lateral views of *L. coromandelica* (US 258427A: C.J. Saldanha 16,725; Hassan Distr., India). **d, e** Lateral view and coronal section of *L. coromandelica* (UC 1037609: W. Koeltz 20,545; India). **f–k** *L. antiscorbutica* (P 5285392: A.R. Torre & M.F. Correia 16,198; Zambezia, Mozambique). **f** Complete fruit with intact pedicel and styles. **g–i** Endocarp in lateral and ventral views. **j** Fruit from the same collection in sagittal section showing curvature of locule. **k** Fruit from the same collection in coronal section. **l, m** Fruit of *L. schimperi* in sagittal and coronal sections showing strongly curved locule; arrows marking edges of the operculum (P 5285113: G. Fotius 2490; S.W. Tchamba, Togo). **n, o** Lateral view and transverse section of *L*. *schimperi* (US 2480904: W. Burger 2596; Herar Prov., Ethiopia). **p** Same as o, enlarged to show endocarp anatomy. **q–t** *L. coromandelica* (UC 1037609: W. Koeltz 20,545; India), C-T scan images. **q** Lateral view of fruit, volume rendering, showing curvature of endocarp and broad apical germination valve. **r** Sagittal section showing rib along apical crest. **s, t** Coronal sections, showing apical rib; arrows marking edges of the operculum. Scale bars 5 mm in a (also applies to b**–**d), e (also applies to f**–**i), and j**–**k, (also applies to l-o), q (also applies r-t); 0.5 mm in p

**Fig. 15:**
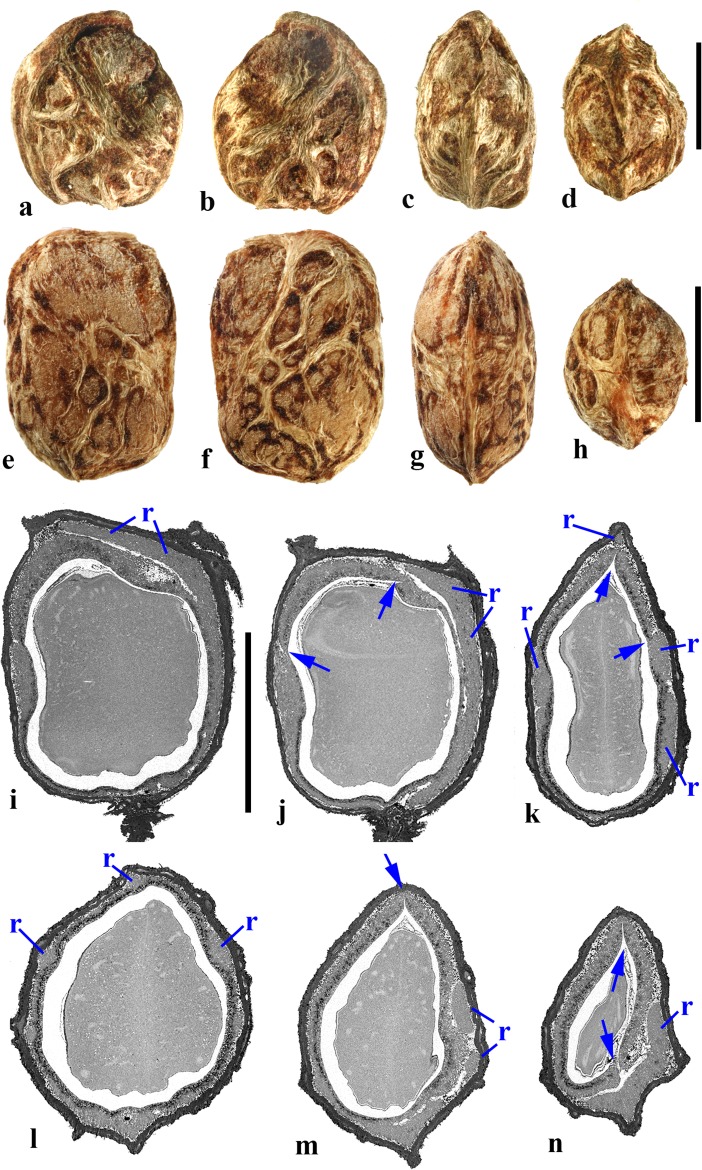
*Lannea*, continued. **a–d** *L. edulis* endocarp in lateral and apical views (UC 1419530: J. Pawek 5953B; Malawi). **e–h** *L. velutina* endocarp in lateral and basal views (P 4852302: P. Jaeger 5025; W. Africa). **i–n** *L. velutina* fruit in SRXTM virtual sections (K 452108: AJM Leeuwenberg 4375; Burkina Faso). **i** Median sagittal section showing pedicel, persistent hypogynous perianth, complete pericarp and the seed. Median rib follows apical crest (r). **j** Sagittal section parallel to i, revealing base of the germination valve (delimited by arrows), and median rib (r). **k** Coronal section revealing two cotyledons within the seed, germination valve on one side (delimited by arrows), and ribs (r). **l** Transverse section near equator, showing ribs (r) in cross section. **m** Transverse section ca. halfway between equator and apex, showing one edge of the germination valve (arrow). **n** Transverse section near apex, showing germination valve delimited by arrows, and fibrous rib (r). Scale bars = 5 mm in d (also applies to a**–**c), and h, i (bar in i also applies to j-n)

The drupes are usually purple-black or red at maturity and show 4 or 5 remnant styles (Von Teichman, 1987) although only one locule develops. A row of secretory cavities was observed along the boundary between exo- and mesocarp (Von Teichman, 1987). *Lannea* endocarps usually have longitudinal and reticulate ribbing visible on both lateral faces (Figs. 14a**–**d, g, h, n and 15a**–**h), which vary from conspicuous to inconspicuous among the species. One of the ribs follows one lateral margin to the apex in the plane of bisymmetry. Particularly in the lower $$ \raisebox{1ex}{$2$}\!\left/ \!\raisebox{-1ex}{$3$}\right. $$of the endocarp, an irregular reticulum is formed together with other ribs, that may delimit prominent depressions (Fig. 15b**–**d). The endocarps are unilocular and more or less bilaterally symmetrical, usually with a keel in the plane of symmetry. *Lannea* endocarps are lenticular or narrowly elliptical in transverse section showing the single locule and absence of lacunae (Fig. 14o). In face view the endocarps have an elliptical to slightly reniform outline. Sagittal sections reveal a strongly curved (Fig. 14j, l, r) to weakly curved (Fig. 15i, j) locule, due to an internal bulge of the endocarp wall on one side, termed the endocarp knob by Von Teichman (1987). This knob is not very prominent in the type species, *L. velutina.*

Von Teichman (1987) reported two or more opercula per fruit in *Lannea discolor*; however, he did not provide photographic documentation. The areas outlined as opercula in the drawings of his Fig. 2 are difficult to distinguish from the sculptural depressions distributed elsewhere over the endocarp surface. Among a sampling of 66 *L. coromandelica* endocarps from a single wild tree (UF2630), 60 specimens were found to show a single operculum confined to one of the lateral faces, and six specimens appeared at first view to have paired opercula, one on either face. However, closer inspection revealed that in these cases there was actually only one operculum which developed on both lateral faces and continuous across the keel. Only a single locule is present in these specimens. Our dissections of multiple species, and X-ray tomography of *L. velutina* and *L. coromandelica,* indicate that the operculum is usually confined to one of the lateral faces near the apex. It is broadly elliptical, contrasting with the narrow-elliptical opercula of several other genera. Usually a valve is visible only on one side of the endocarp, recognizable by its smooth surface contrasting with the reticulately ribbed pattern over the rest of the endocarp (Figs. 14c**–**e, h, m, n and 15a, g, k). The prominent rib along the apical margin of the endocarp (Fig. 15a, i, j) resembles that seen in *Solenocarpus* and *Haplospondias*. The operculum is composed of the same tissue as the rest of the endocarp wall. In some cases, we could not identify any obvious operculum or valve--possibly the borders of the operculum are less obvious in immature fruits. The locular envelope is thin, 0.15 to 0.2 mm, composed mostly of horizontal and periclinal fibers, whereas the rest of the stone is primarily composed of tortuous fibers in various orientations and a surficial network of fiber bundles (mostly forming the external ribs; Fig. 14p).

### *Operculicarya* H. Perr. (Fig. 16)

This genus is known from Madagascar and the Comoros, and Aldabra islands, particularly in arid forest and scrub vegetation. We sampled five species, including the type species *Operculicarya* *hyphaenoides*. Randrianasolo and Lowry (2006) recognized eight species in the genus, and recently added a ninth species (Randrianasolo, 2015). They included *O. gummifera*, which alternatively has been treated in *Poupartia* (e.g., Friedmann, 1994; Randrianasolo & Miller, 1999). The latter species, which has larger leaflets than the other species of *Operculicarya*, has a stone that closely resembles that of the type species of *Poupartia*, *P. borbonica*. Hence we treat that species as *Poupartia*, and consider there to be eight currently recognized species in *Operculicarya*. We studied fruits of *O. calcicola*, *O. decaryi, O. hyphaenoides,* and *O. multijuga* and found them to be consistent in stone morphology and anatomy. Floral and fruit anatomy (Von Teichman & Hardy, 1992), and seed structure and anatomy (Von Teichman, 1992) were studied in detail for *O*. *decaryi*. *Operculicarya* flowers have 5**–**6 styles; however, all but one of the carpels are very reduced and the fruits are typically unilocular.

**Fig. 16:**
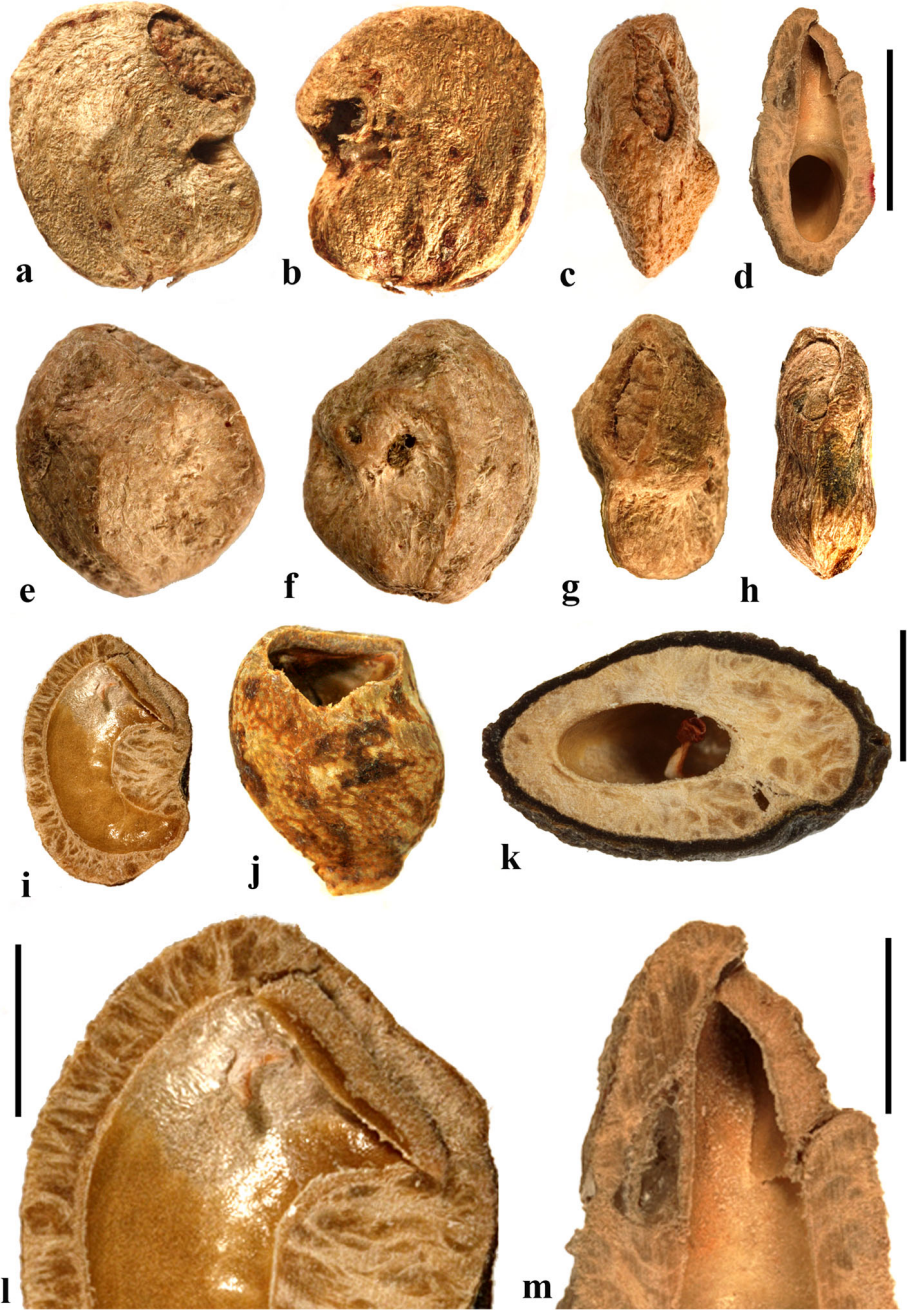
*Operculicarya*. **a–d** *Operculicarya* sp. Lateral view showing apical simple operculum, reverse lateral view without operculum, frontal view showing operculum, and coronal section transecting the operculum (MO5794982: R. Rabevohitra et al. 4263; Antsiranana, Madagascar). **e–i** *O. calicola* in lateral, frontal, dorsal and ventral views, and sagittal section (MO6605509: Gautier et al. 5829 [isotype]; Mahajanga, Madagascar). **j** *O. decaryi* stone with operculum removed (MO 3662939: P. B. Phillipson 2525; Toliara, Madagascar). **k** *O. multijuga* fruit cut transversely with intact meso- and exocarp (MO 6446890D: Roland Rakotondrajaona 398; Antsiranana, Madagascar). **l** Enlargement of apical end of i, note operculum. **m** Enlargement of **d** showing detail of simple operculum and endocarp wall. Scale bar = 5 mm in d (also applies to a**–**c, e**–**j); 2 mm in k**–**m

Drupes are red to purplish black at maturity. According to Von Teichman and Hardy (1992), the mature exocarp is tanniniferous with a well-developed cuticle, ca. 2.6 μm thick. Beneath this is a zone of tanniniferous parenchyma with secretory cavities (or ducts) with closely associated vascular bundles. The endocarp is subglobose and asymmetrical with an inconspicuous keel, laterally compressed and slightly ridged with a prominent alveole on one side (Fig. 16c, h). The endocarp surface is smooth to minutely fibrous (Fig. 16a, b, e, f), sometimes with small scattered depressions (Fig. 16b, f). In lateral and sagittal views the endocarps are strongly c-shaped or reniform (Fig. 16a, b, i). This is caused by a prominent internal bulge on one side of the endocarp wall that is well seen in sagittal section (Fig. 16i, l). In transverse section the endocarp and locule are elliptical in outline, showing the single locule and absence of lacunae (Fig. 16d, k). The usual condition is for fruits to possess one locule and one operculum (Randrianasolo & Lowry, 2006), but Von Teichman and Hardy (1992) observed that about 8% of the fruits of *O. decaryi* possess two locules, often with one of them smaller and presumably abortive; in these bilocular fruits there is an operculum corresponding to each locule. The mechanism of dehiscence is a simple operculum (stopper-like plug), which is thick and woody, located near the apex of the endocarp (Fig. 16a, g, l, m). Typically, the operculum is a simple broadly elliptical to ovate plug, with its long axis opposed to the long axis of the fruit. Von Teichman and Hardy (1992) noted, that in rare cases, bipartite opercula were observed in *O. decaryi*, that separate into two approximately equal parts.

The locular envelope is thin (ca. 0.05 mm), composed of thick-walled brachysclereids forming the shiny lining of the locule (Fig. 16l, m). The rest of the stone is thick and consists mainly of tortuous bundles of thick-walled fibers and brachysclereids. Von Teichman and Hardy (1992) observed that the operculum is delimited by parenchyma and is composed of monomorphic, thick-walled spheroidal and vesiculous sclereids, the latter with undulating outlines usually fitting into each other like pieces of a jig-saw puzzle.

### *Pegia* Coleb. (Fig. 17)

*Pegia* is known from India, Nepal, Bhutan, Myanmar, China, Thailand, Indochina, and Borneo. Two species are recognized, i.e., *Pegia nitida* (the type species) and *P*. *sarmentosa*. We examined specimens of both species (Table 1).

**Fig. 17:**
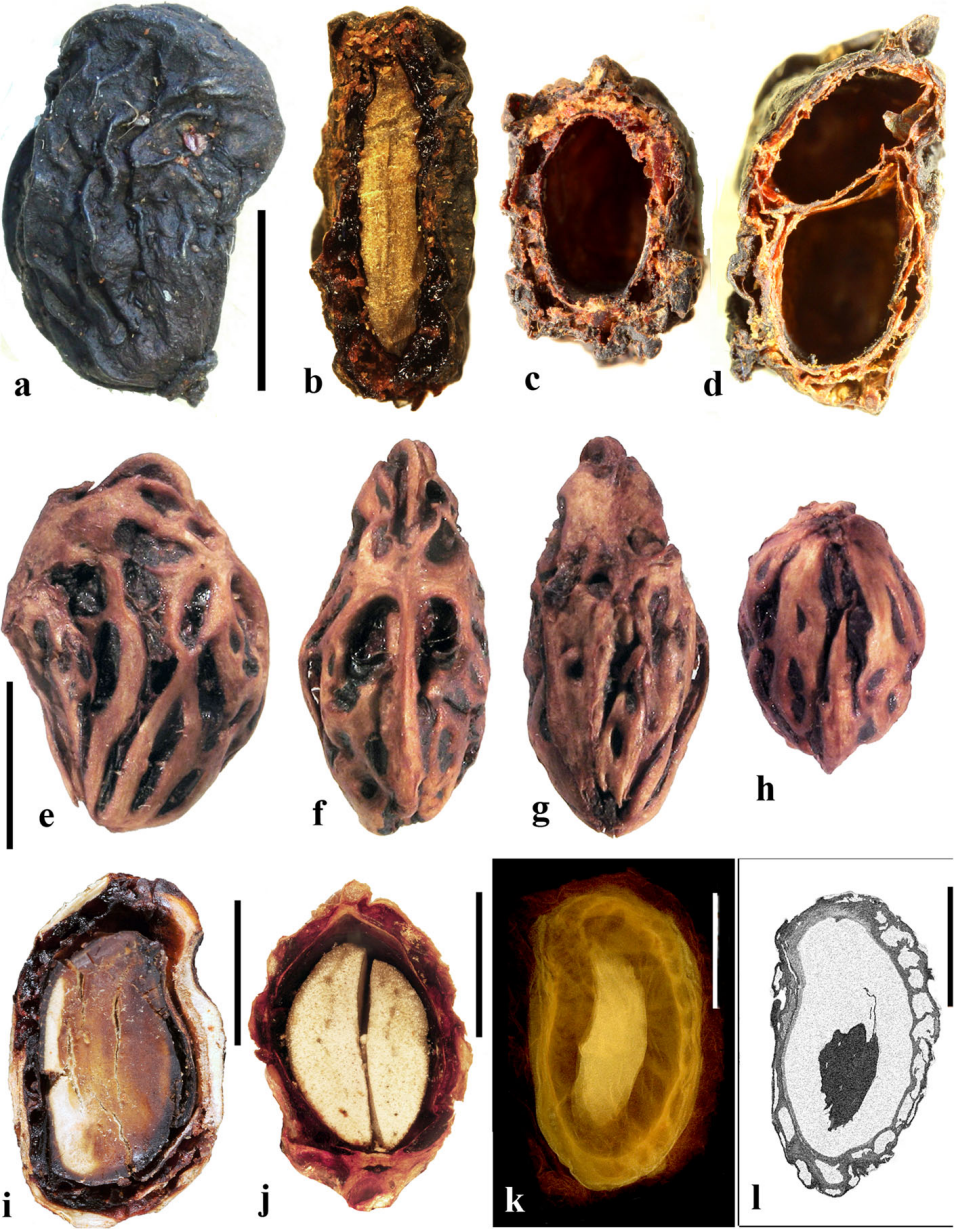
*Pegia*. **a–h** *P. sarmentosa* (P 6634428: L’Abbé Bon 2679; Vietnam). **a** Dried wrinkled fruit viewed laterally. **b** Coronal longitudinal section of complete fruit showing two cotyledons of seed, very thin endocarp, the mesocarp with oozing shiny viscous liquid, and lack of apical plug or valves. **c** Unilocular fruit transverse section. Note very thin endocarp wall. **d** Rare bilocular fruit from the same collection, transverse section. **e-j** *P. nitida* (NY: A. Henry 11729A; Yunnan, China). **e–h** Surface views of endocarp showing ribbed sculpture; lateral, frontal, dorsal and apical views. **i, j** Sagittal and transverse sections of same. **k, l** Digital sagittal sections from CT scan data, *Pegia sarmentosa* (K: L. Lugas 789; Sabah, Borneo). Scale bar 5 mm in a (also applies to b**–**d), e (also applies to f**–**h), i-l

The drupes are red to purple at maturity. The endocarp surface is sculptured with broad depressions between fibrous ribs of the reticulum (Fig. 17e**–**h). The endocarp is obliquely elongate, obovoid, slightly reniform, and bilaterally symmetrical with a keel (Fig. 17f, h). The fruits are typically unilocular (Fig. 17c, j), but we observed a rare bilocular fruit as well (Fig. 17d). In transverse section the endocarps are elliptic (Fig. 17c, j). Sagittal sections show that the long axis of the locule is straight to slightly reniform (Fig. 17i, k, l). There seems to be no obvious germination apparatus but the endocarp wall is so thin that the radicle could probably emerge easily without requiring specialized opercula or valves. It is also likely that germination is facilitated by apical splitting. Lacunae are absent.

The locular envelope in *Pegia* is very thin (0.2**–**0.4 mm), composed of a 1**–**3-seriate layer of brachysclereids. The endocarp wall is composed of thick bundles of fibers (Fig. 17f). Micro-CT scan imagery shows rectangular spaces in the pericarp (Fig. 17k, l), and physical examination revealed a viscous fluid in the wall that remained sticky even in fruits collected more than 80 years ago.

### *Pleiogynium* Engl. (Fig. 18)

**Fig. 18:**
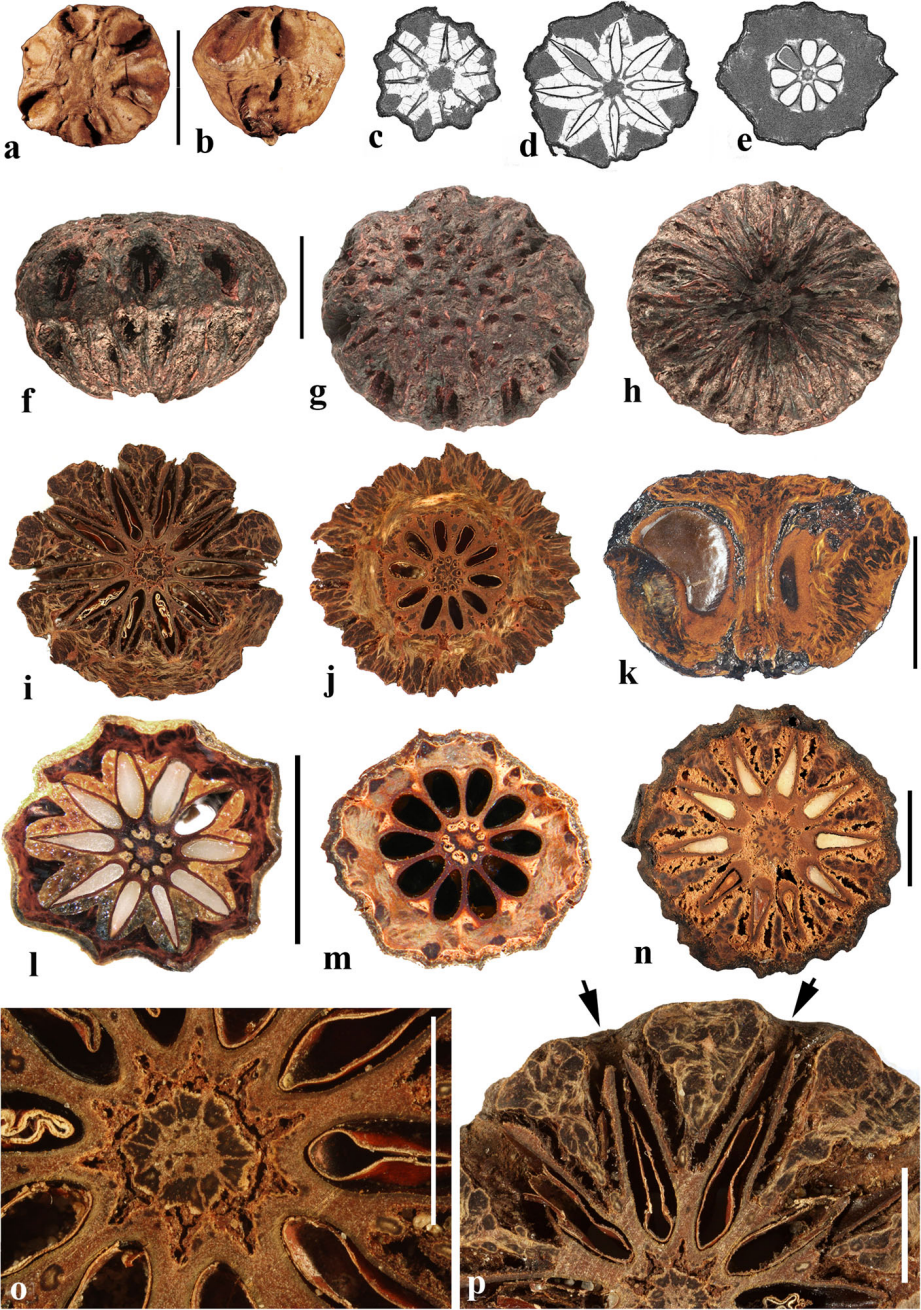
*Pleiogynium*. **a, b** *P*. *hapalum* fruit in apical and lateral views (GH: A.C. Smith 1499; Fiji). **c–e** *P. hapalum* fruit in digital x-ray transverse sections from near apex to near base (US 2190144: A.C. Smith 7164; Fiji). Note prominent lacunae surrounding each locular envelope in **c**, **d**. **f–h** *P. timoriense* lateral, apical, and basal views of stone (UF 1341: M. Pole s.n.). **i, j** Transverse sections of the same fruit as f-h**. i** is in apical area, transecting the germination pores, note recessed internal bilabiate valves. **j** is below the equator. **k** is a sagittal section of another specimen from same collection as f. **l, m** Transverse sections of a fruit of *P*. *hapalum* (NY: P. Cox 1113; Island of Eua, Tonga). **l** is slightly above equator, showing v-shaped lacunae between each pair of locules. **m** is slightly below equator with small triangular lacunae between locules. **n** *P. timoriense,* transverse section of stone (FLAS 158900: W. L. Stern, s.n., 12/26/1985; Homestead, Florida, U.S.A.). **o, p** Detail from i showing circle of 10 vascular bundles and 10 locules. **p.** Detail from i showing seed coat within each locule, and opened germination bilabiate valves within each apical pore (arrows). Scale bars 1 cm in a (also applies to b**–**e), f (also applies to g**–**j), k**–**n; 5 mm in o, p

This genus includes two species in Malesia, Australia, and the South Pacific. Both were sampled for the current study. Fruits of the type species, *P. timoriense* (synonym *P*. *solandri* (Benth.) Engl), from Queensland, Australia (Fig. 18f**–**h) are much larger than those of *P. hapalum* (Fig. 18a, b). Fossil occurrences of *Pleiogynium* include fruits from the Oligocene and/or Miocene of Queensland, Australia (Rozefelds et al., 2015). The same authors questioned the earlier identification of *Pleiogynium* based on a single specimen from the Eocene of Messel, Germany (Collinson et al., 2012); investigation of the Messel fossil is continuing.

The exocarp of *Pleiogynium* is membranous, red to brown or black and depressed over the germination pores (Fig. 18a, b). The mesocarp is fleshy and the endocarps are woody and oblate to globose or turbinate, usually with 8–12 locules--more than observed in all other genera of Spondioideae (Fig. 18a–l). An elliptical apical germination pore is associated with each locule. The pores are radially arranged and located near the periphery of the endocarp (Fig. 18b, d, e). Contrary to the interpretation of Hill (1933), these fruits lack opercula (Rozefelds et al., 2015). Nevertheless, the apical pores are involved in germination. A pair of internal recessed bilabiate valves, through which the radicle must exit, are formed by the distal parts of the locular envelope within each pore (Fig. 18f, i, o, p). In equatorial transverse section the endocarps are almost circular (Fig. 18d, j).

The endocarp in *Pleiogynium timoriense* is conspicuously sculptured with punctae on the apical surface (Fig. 18g) and numerous longitudinal ridges over the lower half (more ridges than the number of locules; Fig. 18h, j). In *P. hapalum*, the sculpture is less prominent, with fewer ridges in the lower half (equal to the locule number, Fig. 18b, e). The prominent basal ridges create abundant apertures, seen as channels in transverse and sagittal sections, but these do not reach the locules (Fig. 18h–j).

Locular envelopes of adjacent locules merge ventrally but are free from each other dorsally, together forming a star-shaped structure as viewed in transverse equatorial section of the endocarp (Fig. 18m, o). Each locule is flanked on both lateral margins by a well-developed lacuna (or parenchyma-filled pocket); so, if there are 10 locules, 20 lacunae are seen in equatorial transverse section (Fig. 18d, l). At higher levels of the same fruit, the lacunae of adjacent locules coalesce, resulting in the appearance of 10 lacunae (Fig. 18c, i, m). Sagittal sections show that the long axes of the locules are strongly reniform and have a phallic shaped distal portion (Fig. 18k).

The locular envelope in *Pleiogynium* fruits (Fig. 18o, p) is composed of a layer up to 0.5 mm thick of periclinal, horizontally arranged fibers (or tangentially elongate sclereids sensu Wannan and Quinn (1990)). Occasional vascular bundles are seen in the septa, in addition to a whorl of prominent vascular bundles surrounding the central axis of the fruit (Fig. 18m). Distal to the locules and lacunae there is a prominent layer of tortuous tracts of fibers about 5 mm thick. The surrounding tissue of the endocarp, forming the thick wall, external to the locules and lacunae, is composed of tortuous tracts of fibers. The mesocarp, in our interpretation, is the fleshy layer to the outside of the stone, but an alternative interpretation is to consider endocarp to comprise only the locular envelopes and treat the surrounding fibrous tissue as inner mesocarp (Fig. 18m) (Rozefelds et al., 2015).

### *Poupartia* Comm. ex Juss. (Figs. 19–21)

This genus of ca. eight tree species (Randrianasolo & Miller, 1999) is native to the Mascarene Islands and Madagascar. *Poupartia* was originally described from the island of Réunion, and indicated as having 5 styles, and a 5-locular fruit (de Jussieu, 1789). The type species, *P. borbonica*, has drupes with 2 to 5 locules. The circumscription of *Poupartia* has been problematic and some species previously assigned to the genus in the compilation of Mattick (1935) have been reassigned to other genera of Spondioideae (e.g., *Antrocaryon amazonicum*, *Choerospondias axillaris*). As the genus is currently circumscribed (Pell et al., 2011), endocarp morphology ranges from irregularly shaped in the Mascarene Island species to smooth ellipsoid and globose fruits among some Malagasy species. All of them have simple opercula. Here we recognize three morphological categories of *Poupartia* fruits.

**Fig. 19:**
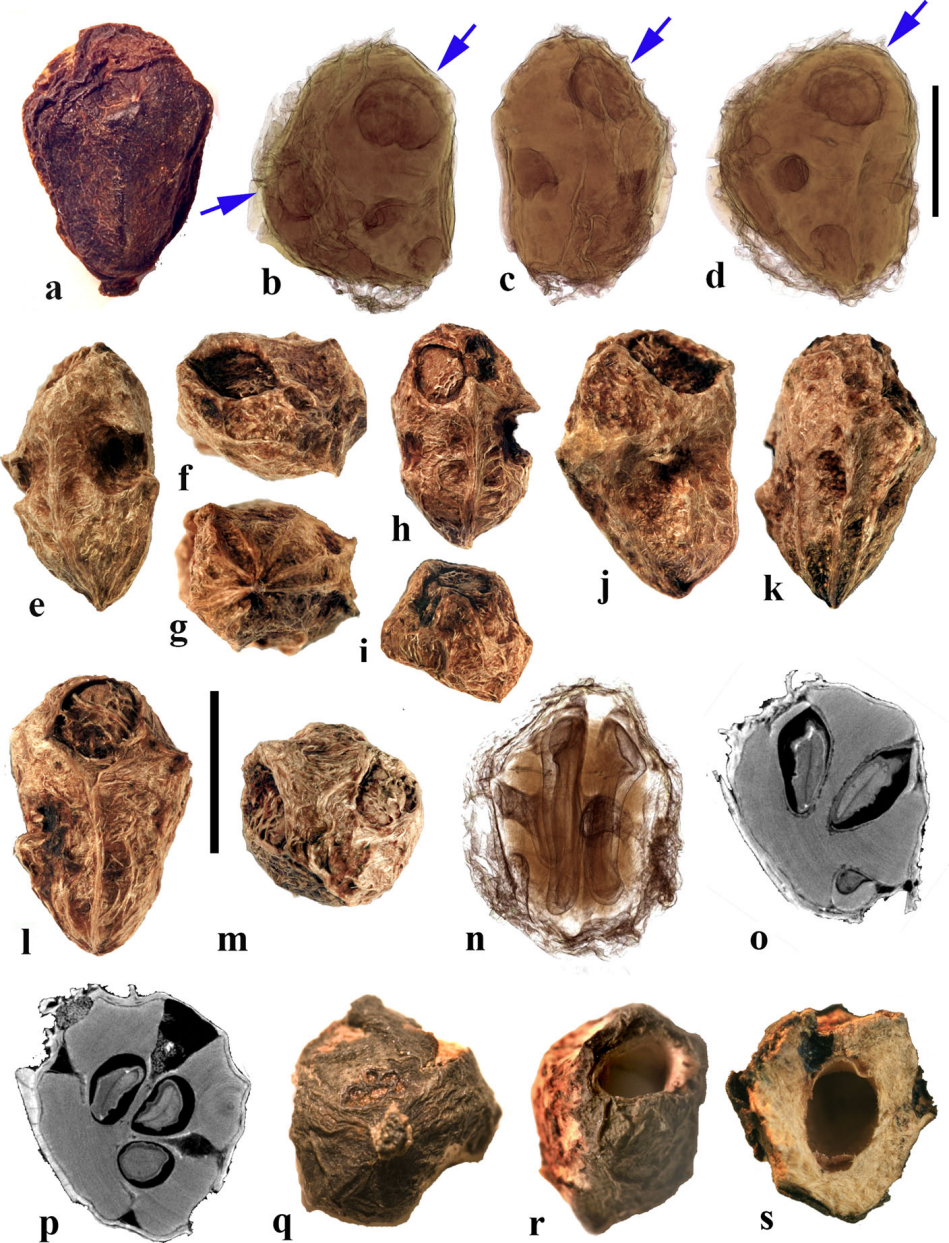
*Poupartia*. **a–d** *P. borbonica* fruit from type collection. **a** Lateral view of holotype fruit showing general shape of dried fruit with mesocarp and exocarp (P JU-15906: Commerson. sn; Reunion Island). **b–d** Another fruit from the isotype collection rotated on long axis to show three lateral views (MPU 20599 Commerson sn in 1717, circa St Paul, Legot, Reunion Island), volume rendering from x-ray data with exocarp and mesocarp translucent showing endocarp with opercula (arrows) and lateral depressions. **e–m** Multiple views of four endocarps of *P. borbonica* (Kew MSB 523570: P. Seepaul 365; Roches Noires, Mauritius). Most specimens observed have one operculum but a few have two (e.g., **m**). **n** Same fruit as b-d, digital longitudinal digital section showing two elongate locules and equatorial lacunae. **o, p** Digital transverse slices of *P. borbonica*, the same specimen as in b-d from CT scan data showing three operculate locules and four septal lacunae connected by channels to the periphery; **o** is closer to the apex, showing germination valves on two of the locules; **p** shows three locules and four lacunae that open toward the periphery. **q–s** *P. pubescens* (K M.1344/79: Horne s.n.; Mauritius) in apical view with the operculum removed, and in transverse section. Scale bar 5 mm in d (also applies to a**–**c), and m (also applies to e**–**l, n**–**s)

**Fig. 20:**
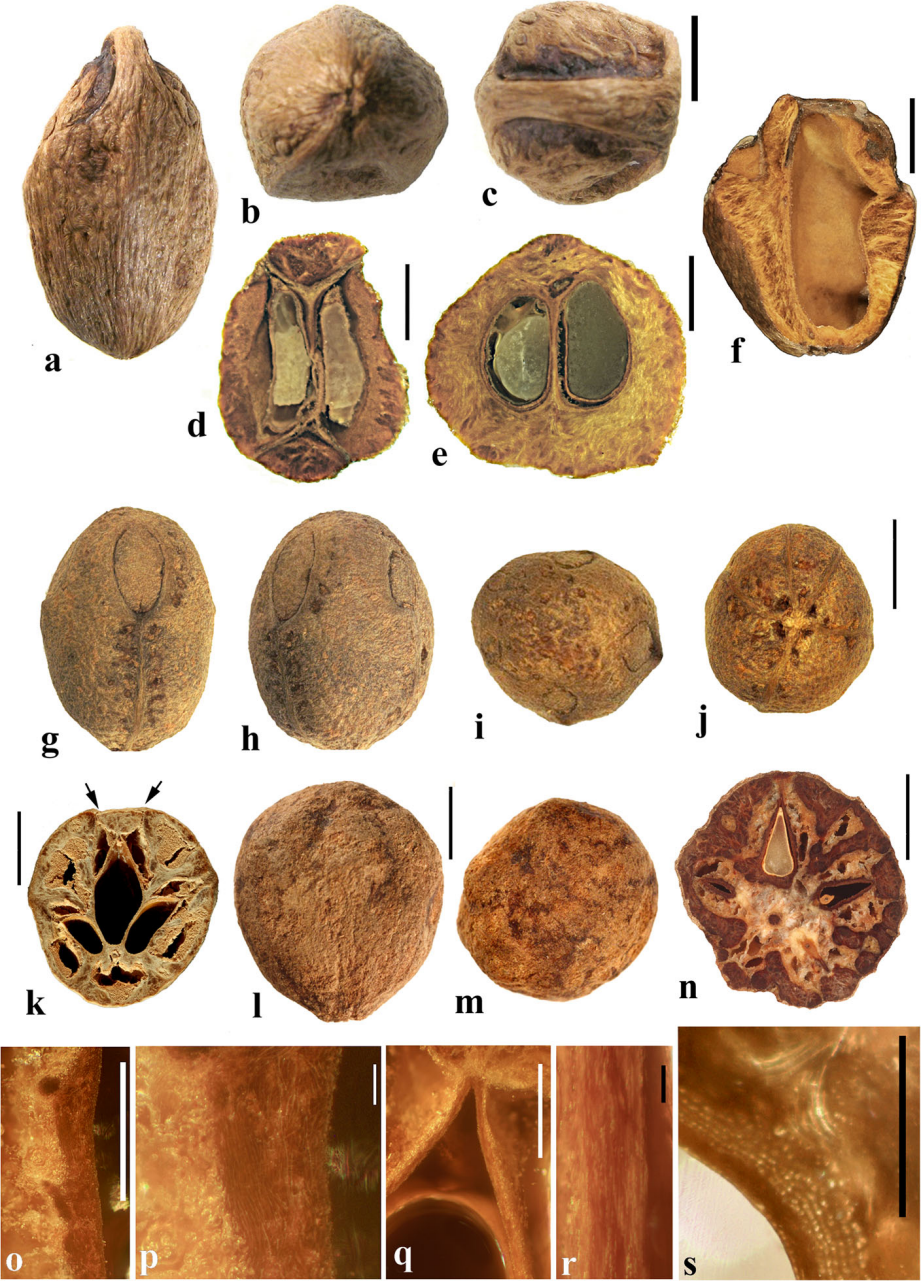
*Poupartia* continued. **a–f** *P. chapelieri* lateral, basal, and apical views, and transverse and sagittal sections (NY: Ravelonarivo & Rabesonina 674; Antsiranana, Madagascar). **g–k** *P. silvatica* Lateral, apical, and basal views and transverse section of the stone; note opercula seen in g**–**i, basal apertures in j, and operculum in transverse section in k (arrows). (MO 6088121: Ranaivojaona 1706). **l–n** *P. orientalis* (MO 5666318: N.M, Andrianjefy et al. 295; Madagascar) longitudinal, apical views and equatorial transverse section. **o–s** *Poupartia* anatomical details. **o, p** *P. orientalis,* enlargements from n**; q, r** *P. silvatica,* enlargements from k. **s** Detail of locular envelope of *P. chapelieri* (Ravelonarivo & Rabesonina 674). Scale bars 3 mm in c (also applies to a, b), f; 2 mm in d, e; 5 mm in j (also applies to g-i), k, l (applies also to m), n; 1 mm in o, q; 0.1 mm in p, r; 0.5 mm in s

**Fig. 21:**
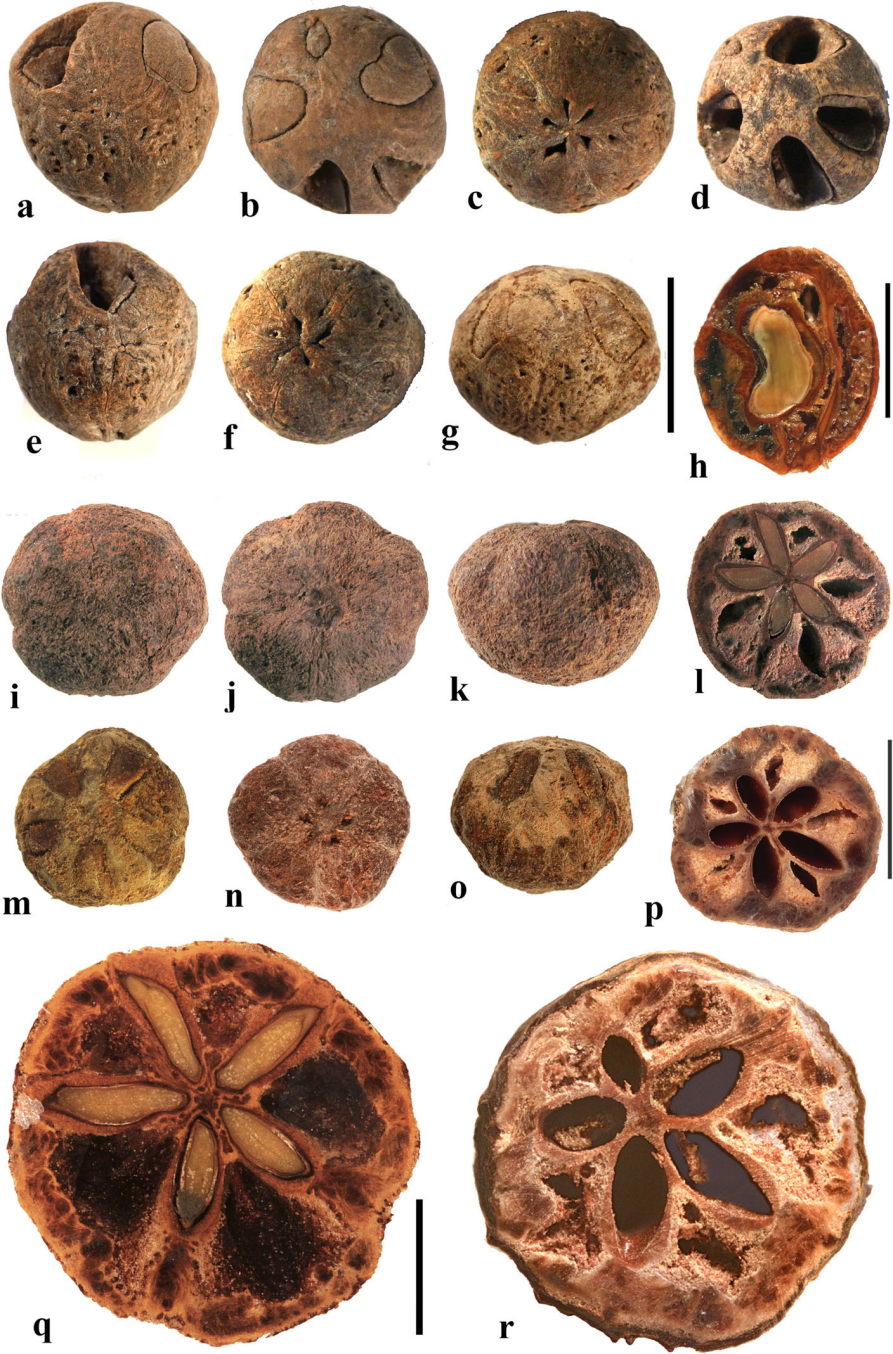
*Poupartia* continued. **a–c** Lateral, apical, basal views of endocarp of *P. minor* (P: M.H. Humbert; carpological coll.; Madagascar). **d–f** Another endocarp from same collection as a**–**c, in apical, lateral and basal views**. g** Apical view of endocarp, *P. minor*. (P 5198195: M. H. Perrier de la Bathie 12,784; Toliara, Madagascar). **h** Sagittal section of *P. silvatica* (F: 2287148: G.E. Schatz 4229; Madagascar) showing curved locule. **i–l** *Poupartia* sp., apical, basal, lateral views, and transverse section (NY: S. Malcomber et al. 2011; Ankarana, Madagascar). **m–p** *Poupartia* sp., apical, basal, lateral views and transverse section (MO 6443312: Tefy Andriamihajarivo et al. 1387; Antsiranana, Madagascar). **q** additional section parallel to that in l, enlarged. **r** Transverse section of globose fruit of *P. minor* (K: M. Adriamahay and S. Rakotoarisoa 2350; Toliara, Madagascar). Scales bars 1 cm in g (also applies to a-f), h (applies also to i-k, m-o) and p (applies also to l); 5 mm in q (also applies to r)

For the first category, we re-examined the holotype and isotype sheets of *P. borbonica* collected by Commerson from Reunion Island in 1771 (Fig. 19a–d, n–p). The fruits have an intact, shriveled pericarp, and none of them have been physically cleaned or sectioned to show clearly the endocarp characters, but one specimen was examined by micro-CT scan (Fig. 19b–d, n–p) to document endocarp morphology. This specimen facilitates the identification of other conspecific material, including similarly small and angular endocarps studied from Mauritius (Fig. 19e–m). Unilocular to trilocular, asymmetrically developed endocarps of *P*. *borbonica* (Fig. 19a**–**p) and *P. pubescens* (Fig. 19q**–**s) are usually obovoid to prolate in shape. The endocarp surface includes prominent longitudinal ribs, and may have prominent irregular depressions (Fig. 19e**–**m). The specimens we examined showed one to three simple apical opercula. The opercula are frequently broadly elliptical and irregular in outline (Fig. 19b**–**d, h, j, l). Transverse sections reveal thick endocarps and elliptical locules (Fig. 19o, p, s). The locules are elongated, slightly curved apically as seen in sagittal section (Fig. 19n). The fruits of *P. gummifera*, placed by some authors in *Operculicarya*, also conform to this morphology. Anatomical details are available from a transverse section of *P. pubescens* (Fig. 19s). The locular envelope is very thin (0.05 mm) and composed of isodiametric sclereids, and the endocarp wall is about 1.2 mm thick, composed of tortuous tracts of fibers.

A second category of fruit morphology in *Poupartia* includes the bilaterally symmetrical bilocular endocarps observed in multiple collections of *P*. *chapelieri* (Fig. 20a–f, s)*.* The endocarps usually show three or more prominent ribs (Fig. 20a–c). Sagittal sections show that the long axes of the locules are straight and have a phalloid apical portion (Fig. 20f). Lacunae are apparently absent. Basal whorled depressions are also absent from these endocarps. The locular envelope, about 0.1 mm thick, is thicker than that in *P. pubescens*, and composed of isodiametric cells (Fig. 20s). Surrounding the locular envelope, the endocarp is composed of tortuous tracts of fibers with interspersed brachysclereids (Fig. 20s).

The third morphological category of fruits *Poupartia* includes the species with pentalocular, prolate to sub globose endocarps that are radially symmetrical externally (Fig. 20g–n and 21a–r) which occur in at least three species of *Poupartia*, i.e., *P*. *minor, P. orientalis,* and *P*. *silvatica*. The endocarp surface is relatively smooth. Transverse equatorial sections show circular endocarps (Figs. 20k, n and 21l, p–r) usually with five locules that are unevenly developed. Typically, they have five lacunae alternating with the locules. The lacunae are usually irregularly shaped, and may coalesce with one another although sometimes they appear triangular in outline when seen in transverse section (Figs. 20k and 21l, p–r). Most of the lacunae appear filled with soft tissue (Figs. 20k and 21h, p–r). The simple opercula are elliptical (Figs. 20g**–**i and 21a, b, e, m). A whorl of small basal apertures similar to that in *Antrocaryon*, *Choerospondias* and *Sclerocarya* is observed around the point of fruit attachment (Figs. 20j and 21c, f, n). The locular envelope is very thin (0.1 mm), composed of dense, periclinal fibers and some sclereids (Fig. 20o**–**r). Between adjacent locular envelopes the septa are composed of isodiametric sclereids or parenchyma cells. The rest of the stone is formed mainly of dense tracts of tortuous fibers (Fig. 21q, r). The opercula are composed of isodiametric sclereids in the inner half, grading to tortuous fibers in the outer portion.

### *Poupartiopsis* Capuron ex J. D. Mitch. & Daly (Fig. 22)

This tree genus has one species, *Poupartiopsis spondiocarpus* found in sandy coastal forests in Madagascar (Mitchell et al., 2006).

**Fig. 22:**
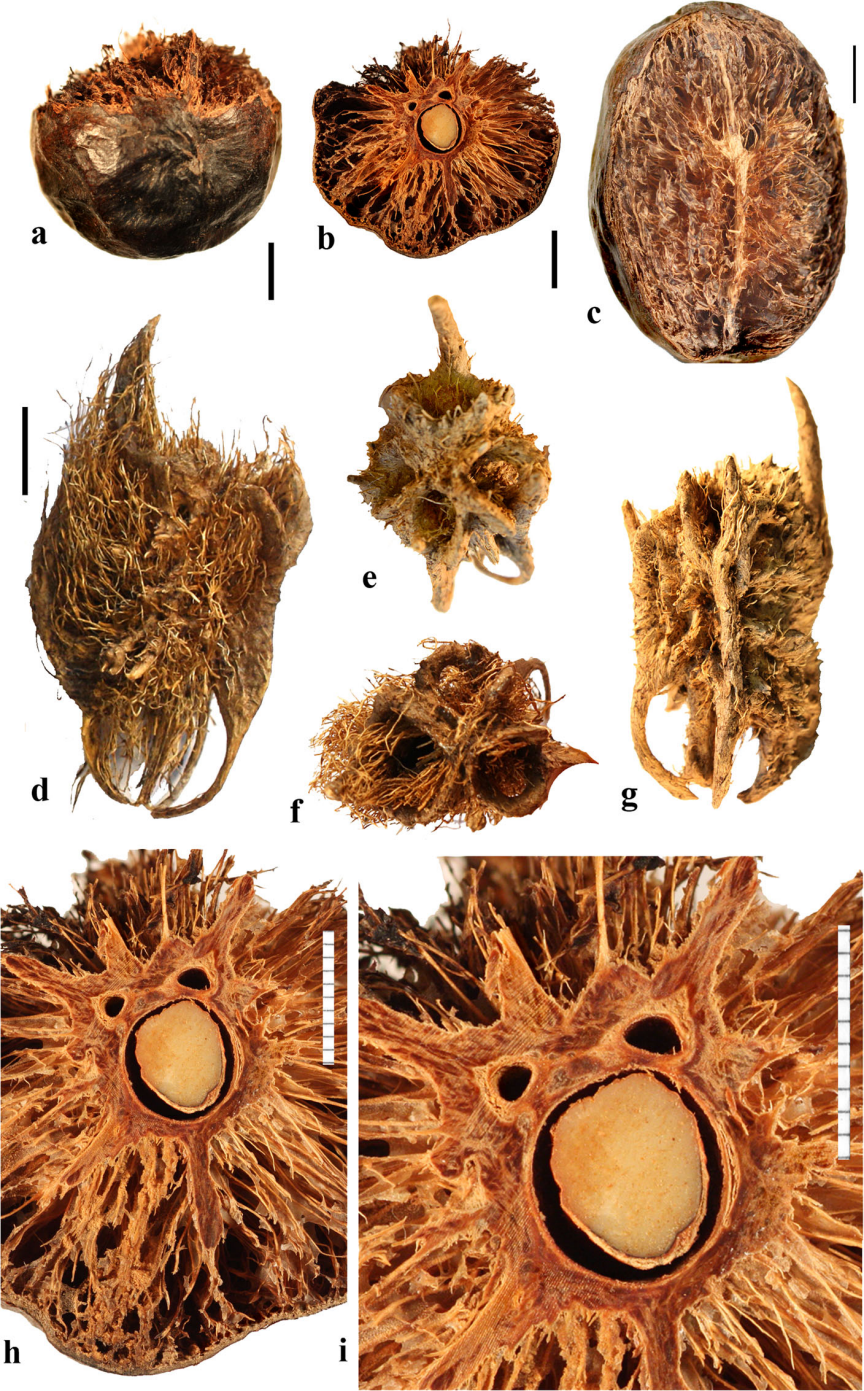
*Poupartiopsis spondiocarpus.* **a–c** Basal view, equatorial transverse section, and lateral view, of fruit with the mesocarp and exocarp removed from one half (MO 4816066: Capuron 18,188-SF; Toamasina, Madagascar). **d** Endocarp in lateral view with mesocarp removed showing spines (P: Capuron 8930-SF; Tenina, Madagascar). **e** Endocarp in apical view showing prominent spines and four germination pores (P 5198123: Service Forestier Madagascar s.n.; Madagascar). **f** Apical view of the specimen in d, showing three germination pores. **g** Lateral view of the specimen in e. **h, i** Successive enlargements of **b**. Scale bars 1 cm in a**–**c, d (also applies also to e**–**g), h and i

The fruits are the largest of all Spondioideae, 6.2–6.6 long by 3.3–4.6 cm in diameter. Exocarp is black, about 0.1 mm thick (Fig. 22a). Mesocarp is thin and coriaceous. The endocarp is oblong-ovoid or slightly oblong-obovoid, rounded apically and truncate basally with prominent basal and apical spines (Fig. 22d**–**g). Tetra- and trilocular endocarps were observed (Fig. 22e, f), and we sectioned a specimen with one functional and two abortive locules (Fig. 22b, h, i). The endocarp has numerous long bristle-like endocarp processes radiating from a central woody core. Longitudinal plate-like ribs extend outward from the distal side of each locule; between these ribs radiate numerous terete fibrous strands, organized like the bristles of a hair brush (Fig. 22d, f**–**i). The bristle layer is about 1.5**–**1.8 cm thick and radiates apically, basally, and laterally from the endocarp; flat, papery layers of fibers interconnect some of these bristles, but mostly the intervening area is hollow. The bristle layer is surrounded by a smooth leathery mesocarp plus exocarp, which together are about 0.5 mm thick. The entire fruit is very light in weight such that it would float easily. Opercula are lacking, but there is a prominent apical pore in the endocarp corresponding to each locule (Fig. 22e, f). Within these pores can be seen the apical portion of the locular envelope. We infer that the seeds germinated with a recessed, bilabiate mechanism.

The locular envelopes are thin (0.15**–**0.2 mm), composed of circumferential fibers. These are surrounded by a dense layer enveloping all three locules composed of variously oriented interwoven tracts of fibers (Fig. 22i). The fibrous composition of the stone resembles that of *Spondias*.

### *Pseudospondias* Engl. (Fig. 23)

This genus of trees and tall shrubs has two species in riverine and rain forests of sub-Saharan Africa. We examined fruits of both species.

**Fig. 23:**
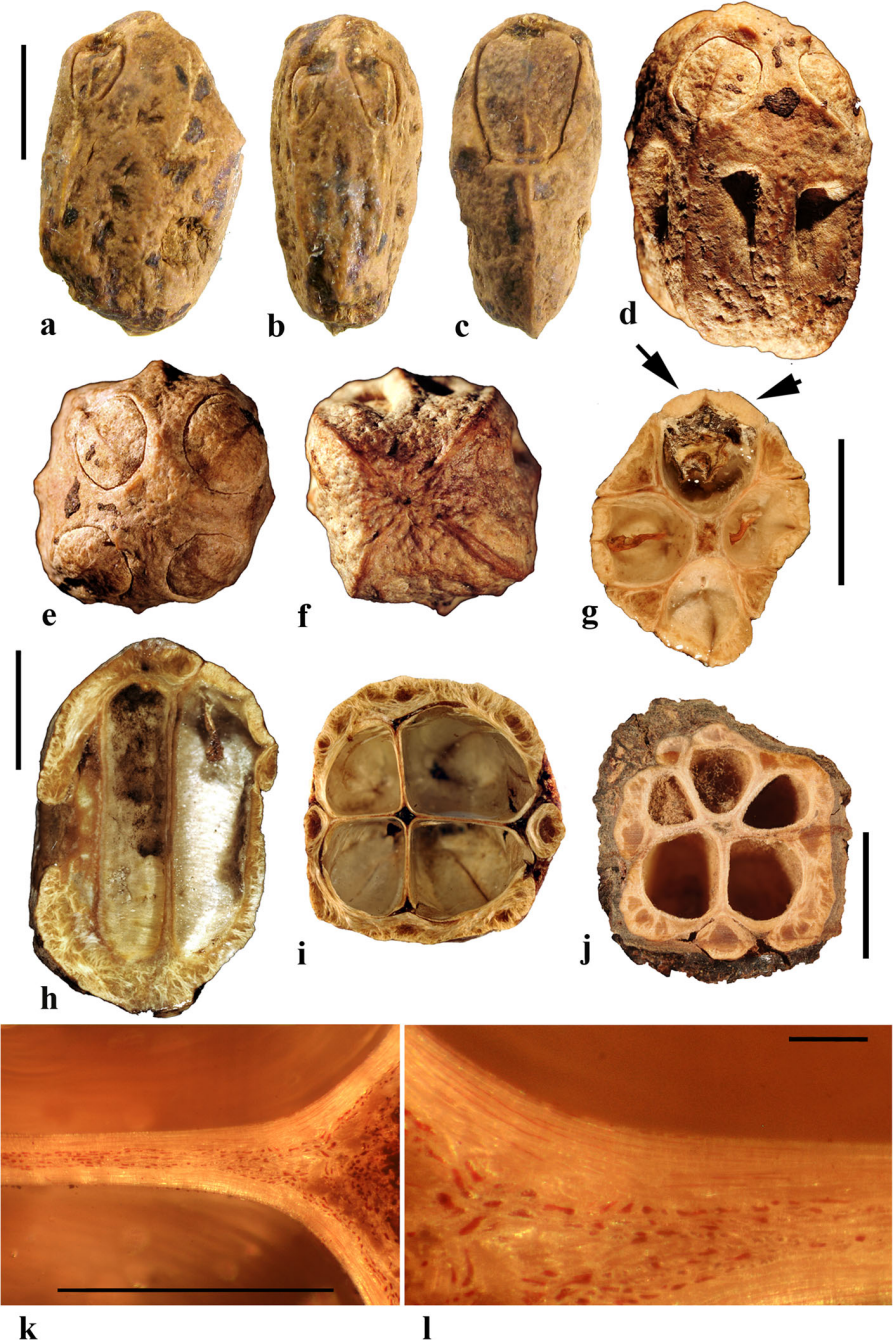
*Pseudospondias*. **a–c** *P. longifolia,* lateral views (NY: J.M. Reitsma 1339; Gabon). **d–h** *P. microcarpa* lateral, apical, and basal views, transverse and sagittal sections (UF 1431: A. Randrianasolo 809; Gabon). Arrows indicate bipartite germination valves. **i** *P. microcarpa*, transverse section of tetralocular fruit (NY: D.W. Thomas and Fay 7284; International Frontier Sangha River). **j** *P. microcarpa*, transverse section of pentalocular specimen with intact exocarp (MO 3097751: W. Meijer 15,034; Cameroon). **k** Anatomy of septum from g. **l** Enlargement from k, showing locular envelope composed of fibers and adjacent tissue of isodiametric cells. Scales bars 5 mm in a (also applies to b, c), g (also applies to d**–**f), h, j (also applies to i); 1 mm in k; 0.1 mm in i

The drupes mature purple. The mesocarp is about 1.2 mm thick in dried condition and usually resinous. The surface of the endocarp is mostly smooth with prominent longitudinal ridges (Fig. 23a**–**f). The endocarps are prolate with usually 4 or 5 locules (3**–**6). Each locule has an apical elliptical bipartite operculum (Fig. 23a**–**e, g). Each operculum has a median line of separation visible at the surface. At germination, they open like a pair of shutters (Hill, 1937). There are also elliptical depressions below the equator that alternate in position with the locules (Fig. 23d). In transverse equatorial section the endocarps appear subquadrangular to subtriangular in outline (Fig. 23i, j). The locules are more or less rounded to almost triangular, and can be equally to unevenly shaped when seen in transverse sections. Sagittal sections show that long axes of the locules are mostly straight with a phalloid distal portion (Fig. 23h). Lacunae are inconspicuous at maturity, but sometimes visible as tangentially flattened spaces at junction of septa with endocarp outer wall (Fig. 23i).

The locular envelopes in *Pseudospondias* fruits are 0.1**–**0.2 mm thick, consisting of horizontal and periclinal fibers encircling the locule (Fig. 23h**–**l). The remainder of the stone is formed mainly of tortuous fibers, but with isodiametric sclereids intervening between adjacent locular envelopes (Fig. 23i**–**l). Typically, there is only a very thin septum between adjacent locules. The opercula show the same wall stratification as the rest of the stone—a lining of periclinal fibers continuous with the locular envelope, grading into tortuous fibers mostly anticlinally arranged in the outer portion.

### *Sclerocarya* Hochst. (Fig. 24)

This genus of small trees and bushes occurs in Madagascar and sub-Saharan Africa. There are two species: *Sclerocarya birrea*, the type species (with three subspecies), and *S. gillettii* (Kokwaro & Gillett, 1980).

**Fig. 24:**
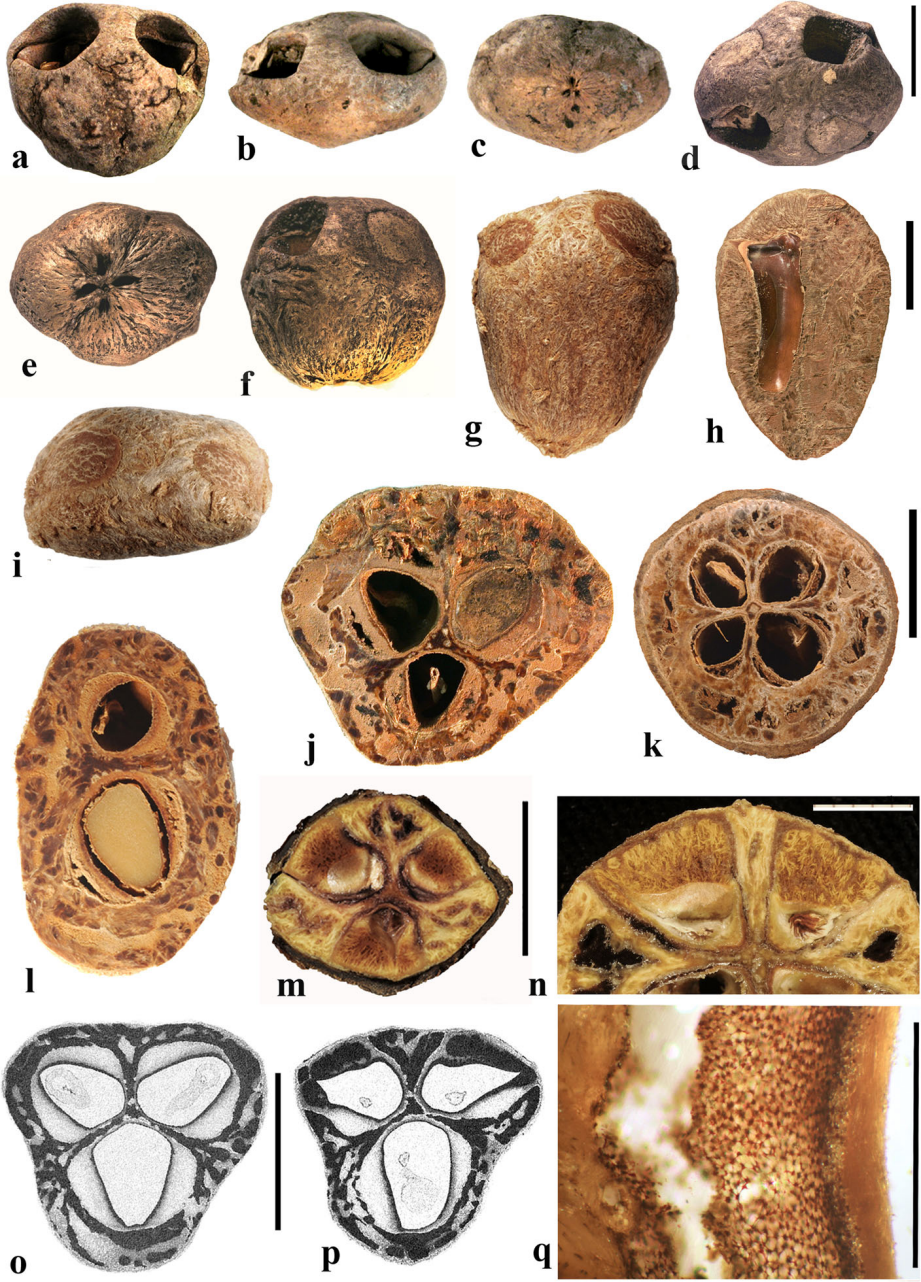
*Sclerocarya.* **a–c** Lateral, apical, and basal views of *S. gillettii* (Kew H2002/03216 68/68: J.B. Gillett 21,245/C; Wajir District, Kenya). **d–f** *S. caffra* (Kew carpological coll.: Barnes s.n.; Mangwe River, S. Africa). **g–i** *Sclerocarya birrea,* lateral and apical views of endocarp with intact germination plugs (US 3515123: J. Wen 9481; Madagascar). **h** Longitudinal section of endocarp showing a locule in sagittal section, *S. birrea* ssp. *caffra* (Kew H2725/60259: A.A. Bullock 1316; Kaputa, N. Rhodesia). **j** Transverse section, trilocular fruit of *S. birrea* (BARC SPI63499: A. Bircher 162; Egypt). **k** Transverse section, tetralocular fruit of *S. caffra* (A: E.H. Wilson sn, January 23, 1922; Rhodesia). **l** Transverse section of 2-locular fruit from g-i. **m** *S. birrea* transverse section near apex, showing germination valves (MO 3497063: J.S. Miller et al. 1066; Foret de Orangea, Madagascar). **n** Enlargement of m. **o, p** Equatorial and near-apical virtual transverse sections of trilocular fruit from CT scan data of *S. birrea* (UF2535: Manchester sn; cult. Brisbane, Australia). Note prominent lacunae on both sides of each locule**. q** Anatomy of endocarp from l, with fibrous locular envelope at right, flanked by parenchymatous tissue of lacuna. Scale bars 1 cm in d (applies also to a**–**i), k (applies also to j, l), o, p; 5 mm in n; 1 mm in q

The ripe fruit is yellow to orange at maturity with a thick, fleshy mesocarp. According to Von Teichman and Robbertse (1986), the mature exocarp *s*.*l.* includes parenchymatous layers with secretory canals, and is developed from the outer epidermis and outer layers of ovary wall, while the mesocarp is parenchymatous with secretory tissue and develops after differentiation and lignification of the endocarp. The endocarp is obovoid to subglobose (Fig. 24a**–**i) with 2**–**4 locules (Fig. 24j–m). Bilocular fruits are approximately bisymmetrical (Fig. 24a, g**–**i) while 3- to 4-locular fruits are more or less radially symmetrical (Fig. 24d, e). The endocarp surface is smooth to finely striate (Fig. 24a**–**i). A basal whorl of apertures, around the point of fruit attachment, corresponds to the number of locules (Fig. 24c, e); these apertures align with the septa rather than the locules. Transverse equatorial sections show elliptical, subangular, triangular, and nearly circular endocarp outline (Fig. 24j**–**m, o, p). Locules viewed in transverse section are usually elliptical in outline and seem to be evenly developed regardless of their number (2, 3, or 4), without obvious abortive portions. Small, irregularly shaped cavities are abundant and dispersed in the endocarp wall (Fig. 24j**–**l, o, p). A crescent-shaped lacuna, sometimes with parenchyma persisting at maturity, flanks both sides of each locule (Fig. 24j–l, o, p). Sagittal sections show that long axes of the locules are straight to slightly curved (Fig. 24h; Fig. 15 in Von Teichman & Robberts, 1986). Each developed locule is associated with a simple operculum (stopper-like plug) (Fig. 24a, b, d, f, g, i, m, n). The opercula are elliptical to broadly elliptical and are located near the apex of the endocarp (Fig. 22m, n).

Locular envelopes in *Sclerocarya* fruits are 0.3**–**0.6 mm thick, and formed by dense tissue of horizontally encircling periclinal fibers (Fig. 24o**–**q). Opercula are formed of tortuous fibers mostly anticlinal in orientation plus sclereids (Fig. 24n) with composition similar to the rest of the endocarp. In micro-CT scan imagery as well as light microscopy the opercula are seen as an abrupt apical thickening of tissue external to the locular envelope (Fig. 24n, p).

### *Solenocarpus* Wight & Arn. (Fig. 25)

*Solenocarpus* occurs in southern India and Malesia. *Solenocarpus indicus* is a tree while *S. philippinensis* can be a tree, (hemi-)epiphyte or liana (Kostermans, 1981, 1991). The flowers have one carpel, and the single style is clavate and partially lateral or apical.

**Fig. 25:**
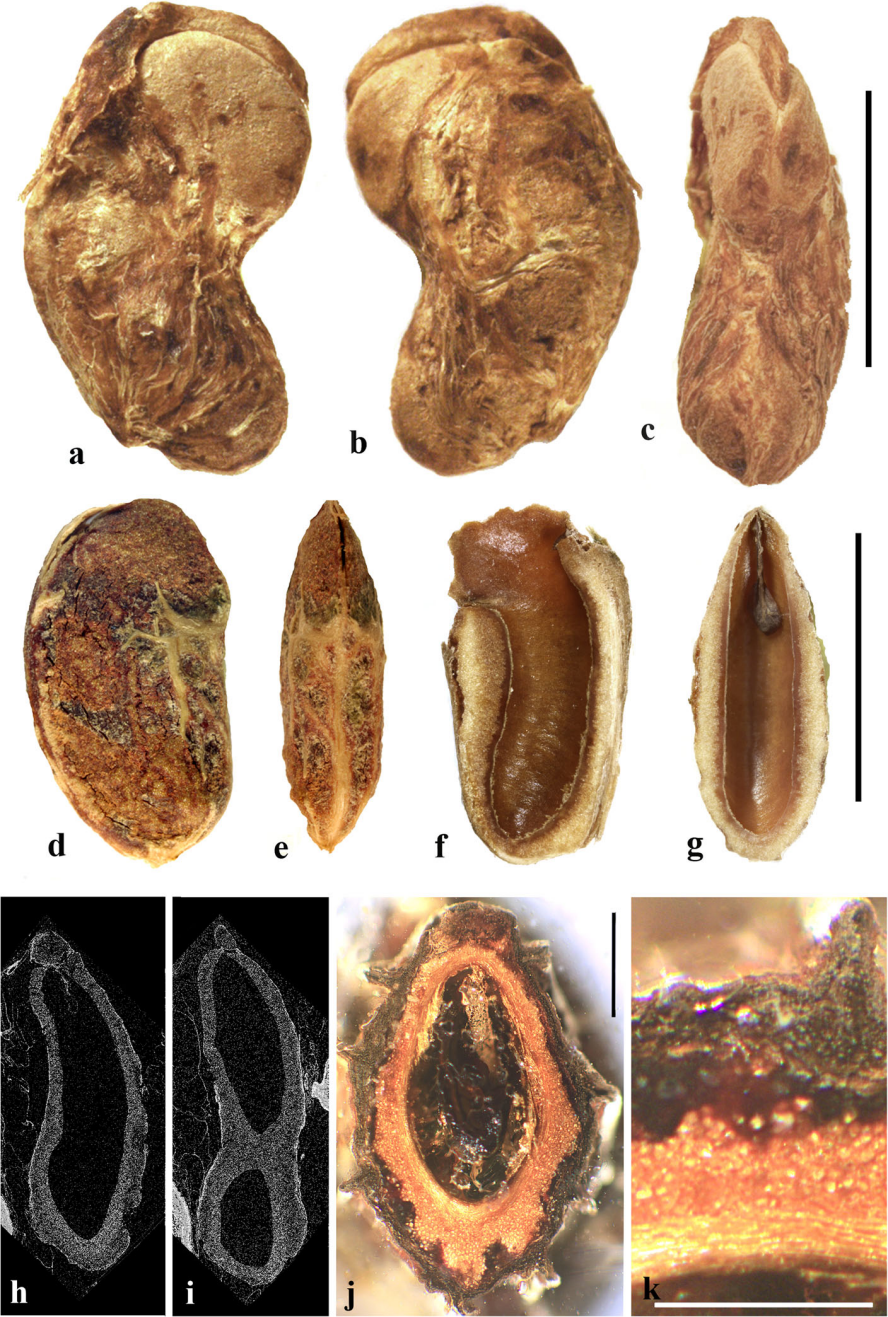
*Solenocarpus*. **a–c** Left and right lateral and front views of *S. indicus* (UC 1342531: T.R.Chand 2938; Assam, India). **d, e,** Lateral, and front views endocarp of *S. philippinensis* (US 3002912: Kostermans 10,502; East Borneo). **f, g** Sagittal and coronal sections of *S. philippinensis* (US 2450012: D. Mendoza 42,448; Mindanao, Philippines). **h, i** Digital coronal sections of *S. philippinensis* (A: J.S. Burley 4276; Sulawesi). **j, k** Transverse section of *S. philippinensis* (US 3002912). Scale bars 5 mm in c (also applies to a, b), g (also applies to d**–**f, h, i); 1 mm in j; 0.5 mm in k

The mature drupe is yellowish, with a thin fleshy mesocarp and a thin-walled (0.4**–**0.5 mm thick) woody endocarp (Fig. 25a**–**g). Endocarps of both species are morphologically similar, but those of *S. indicus* (Fig. 25a**–**c) are much larger than those of *S. philippinensis* (Fig. 25d**–**g). The endocarp is unilocular and laterally compressed with a keel in the plane of bisymmetry, and is slightly to strongly reniform when seen in lateral view (Fig. 25a, b, d, h, i). The endocarp surface has inconspicuous longitudinal and reticulate ribbing. Sagittal sections reveal an internal bulge on one side of the endocarp wall about which the long axis is somewhat curved (Fig. 25f). In transverse section (Fig. 25j) the endocarp and locule cavity are lenticular to elliptical in outline. Lacunae are absent. The germination mechanism is “external bilabiate” with a pair of valves located in the apical 1/4 of the endocarp (Fig. 25a, c, d**–**g). The germination valves are smooth, widely elliptical to reniform; they extend the full width of the fruit and split apart from each other along the apical keel. A prominent terete longitudinal rib of fibers extends along one of the lateral margins and over the apical keel.

The locular envelope is 0.1 mm thick, composed of encircling horizontal fibers, surrounded by endocarp tissue 0.3**–**0.6 mm thick composed of isodiametric sclereids (Fig. 25j, k). The layer of isodiametric sclereids is sculptured with some ribbing over the main body of the fruit, but the apical valves are smooth. Fibers are absent in the endocarp, contrasting with the anatomy of various other Spondioideae (Fig. 25h, i). The endocarp morphology and anatomy are very similar to that of *Haplospondias*.

### *Spondias* L. (Figs. 26–28)

This tree genus of about 18 species is distributed in the Neotropics, Madagascar, India, Myanmar, Sri Lanka, China, Thailand, Indochina, Malesia, and the South Pacific Islands. The type species, *Spondias mombin,* of Mexico to Bolivia and eastern Brazil, and six other species, were sampled. The New World species of the genus were recently monographed (Mitchell & Daly, 2015; this work also provided observations on Old World taxa). Permineralized fruits of *Spondias* have been recognized recently from the early Miocene of Panama (Herrera, 2014).

**Fig. 26:**
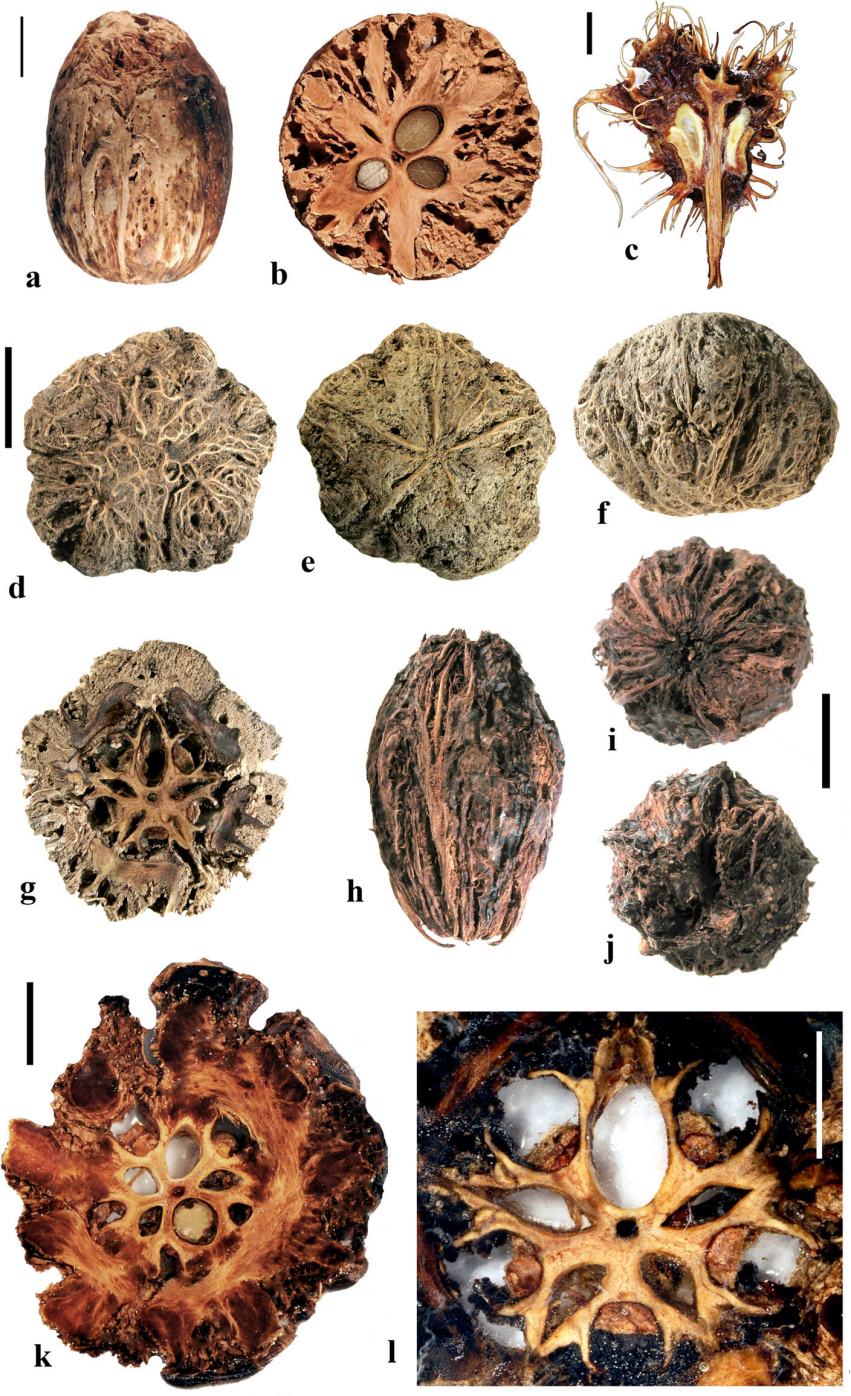
*Spondias*. **a–b** *S. dulcis* in lateral view and transverse section (US 3298668: F.R. Fosberg 60,401; Trak-Moen Is., Caroline Islands). **c** *S. dulcis* endocarp denuded of spongy tissue, in longitudinal section (UF 163: P Grote; Thailand). **d–g,** Apical, basal, lateral, and transverse views *S. globosa* (NY: Gentry 68,896; Tambopata, Peru). **h–k** *S. macrocarpa* drupe in lateral, basal, apical, and transverse equatorial views (NY: W.W. Thomas and R.T. Pennington 6823; Bahia, Brazil). **l** Detail of transverse section of g. Scale bars 1 cm in a (also applies to b), c, d (also applies to e**–**g), i (also applies to h, j); 5 mm in k, l

**Fig. 27:**
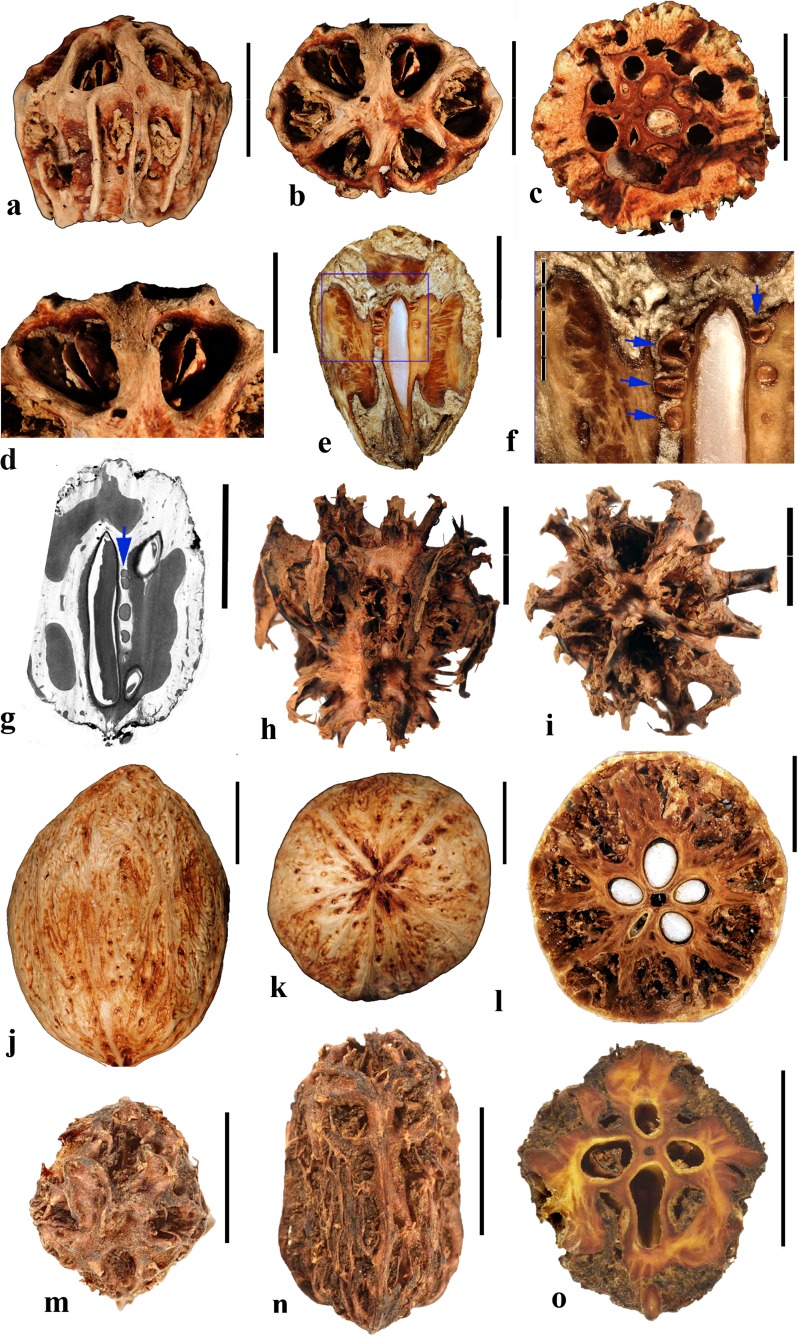
*Spondias*, continued. **a, b** *S*. *mombin* (UF 1601: V. Call s.n.; Florida), lateral and apical views. **c** *S. mombin* (UF 1594: V. Call s.n., Florida) transverse section. **d** Enlargement from b, showing recessed bilabiate germination mechanism**. e, f** *S*. *mombin* longitudinal section (UF 38: Dilcher s.n.; Guanacaste, Costa Rica), blue rectangle in **e** is enlarged in **f**, showing axial rows of globose secondary lacunae (arrows). **g** *S. mombin* (UF 1157: D.L. Dilcher s.n; Brazil), longitudinal section from CT scan data intercepting locule and adjacent row of orbicules (arrow). **h, i** *S*. *novoguineensis* (US 2484723: J. J Havel & N.G.F. School 17,382; New Guinea), oblique and apical views of endocarp. **j–l** *S. pinnata* lateral and basal views and transverse section (UF 40: P. Grote 162; Thailand. **m–o** *S. purpurea* apical and lateral views and transverse section (GH: E. Palmer 408; Jalisco, Mexico). Scale bars 1 cm in a-c, e, g-o, 5 mm in d, f

**Fig. 28:**
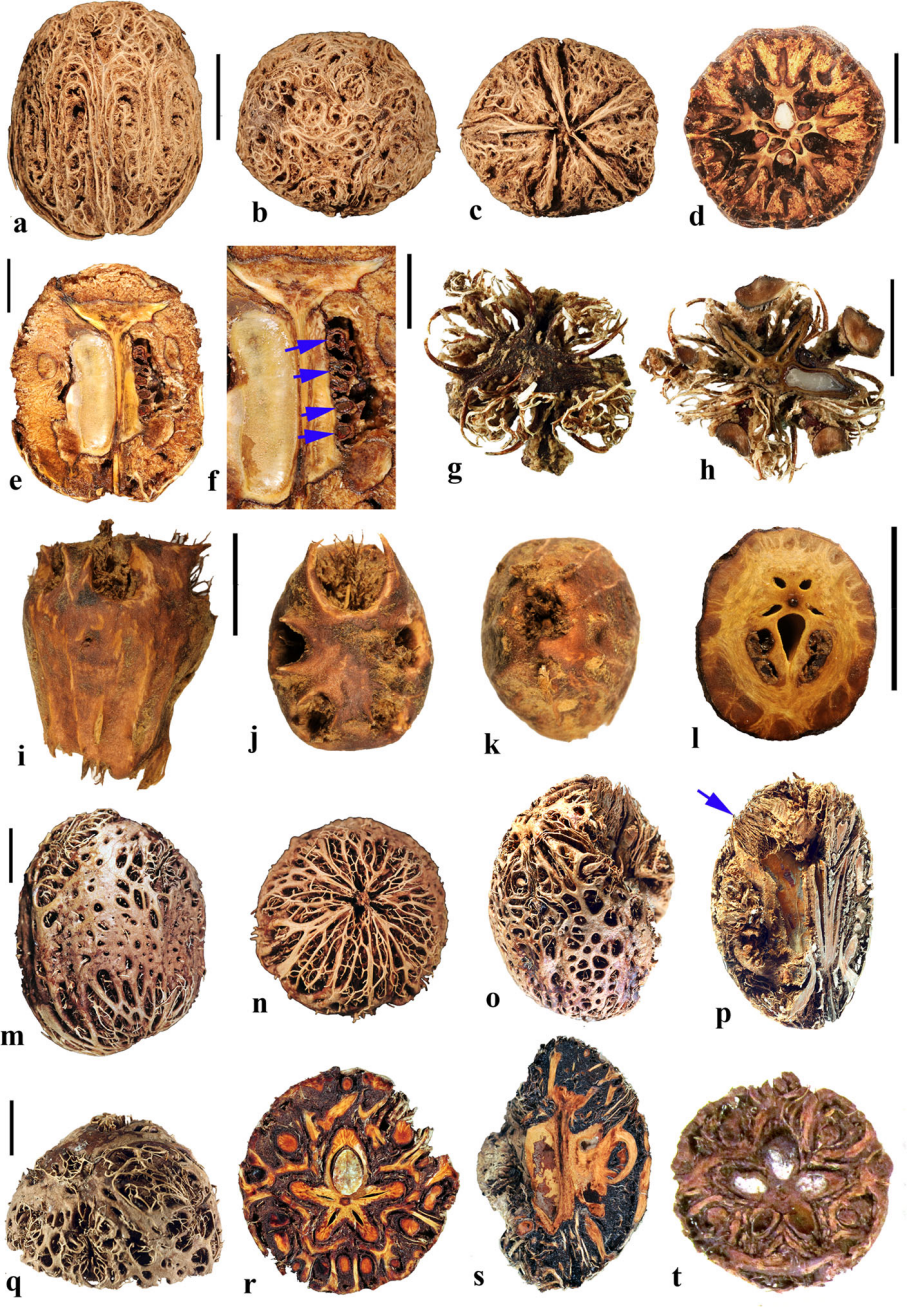
*Spondias*. **a–h** *S. purpurea* (UF 1435: S. Pell s.n.; Costa Rica) in lateral, apical, basal views, and transverse and sagittal sections. Note the vertical row of orbicules (arrows). **g** and **h** with soft tissue removed showing detail of cryptic pores in apical and basal views. **i–l** *S*. *tuberosa* (US 2701812: G. Eiten & L.T. Eiten; Maranhao, Brazil), in lateral, apical and basal views, and transverse section. **m–s** *S. bipinnata* in lateral and apical views and transverse and sagittal sections (UF 2219: S. Manchester s.n.; Chai, Badan, Thailand). Arrow indicates region of dense hair-like fibers occluding the endocarp pore connecting to the locule. **t** *S. bipinnata* transverse section. (A: S. Chongko 675, Kanchanaburi Prov., Thailand). Scale bars 1 cm in a (also applies to b, c), d**–**f, h (also applies to g), i (also applies to j, k), l, m (also applies to n**–**p), q (also applies to r-t)

*Spondias* fruits are typically large and prolate, but oblate fruits also occur in some species. The fruits have a thick to thin edible, fleshy mesocarp; the exocarp may be yellow-orange, red-purple, or greenish (Pell et al., 2011). The usual number of locules is 5, but 4 and 6 also occur. Fibrous tissue surrounds the locular envelopes to form a hardened central endocarp, and also radiates outward in the form of spines or bristles penetrating a spongy parenchymatous tissue, around which a peripheral fibrous network may be formed that lies inside the fleshy mesocarp. When the thin outer layer of fibers and the spongy tissue are physically removed, or naturally eroded, a resistant inner core of endocarp is revealed, composed of numerous radiating straight to curved spines (Figs. 26c, 27h, i and 28g, h). These well sclerified fibrous ribs and bristles, penetrating a softer tissue of parenchyma and/or brachysclereids are a defining feature of *Spondias* fruits. Endocarps of several species of *Spondias* were illustrated and described by Hladik & Hallé (1979) and Mitchell & Daly (2015). Typically, the fruits have cryptic pores (hidden within the spongy tissue) with recessed internal bilabiate germination valves.

We found that Neotropical species, including *S. globosa*, *S. macrocarpa*, *S. mombin*, *S. purpurea, S. radlkoferi* and *S. tuberosa* are also characterized by regular vertical rows of five to eight suborbicular cavities that alternate with the locules and lacunae (e.g. Fig. 27e**–**g and 28e, f). The function and contents of these orbicules, which are typically 1 to 2 mm in diameter, are unknown. Each is encased in a thin membrane that is in contact with sclerenchymatous tissue ventrally but with parenchymatous tissue or a larger lacunal area on its dorsal side. We did not observe these serial orbicules in Old World representatives including *S. bipinnata*, *S*. *dulcis* and *S. pinnata*.

Among the Neotropical species, *S*. *macrocarpa* (Fig. 26h**–**k) and *S. purpurea* (Fig. 27m**–**o and 28a**–**c) have oblong to slightly obovoid fruits. *Spondias globosa* has globose to oblate, rarely very slightly oblong or obovoid fruits (Fig. 26d**–**g, l). *Spondias mombin* has globose, oblong, ellipsoid, and oblong-ovoid to obovoid fruits (Fig. 27a–g). *Spondias tuberosa* has obovoid to subglobose fruits (Fig. 28i**–**l).

As viewed in transverse section, the central hardened portion of the endocarp in *S. globosa*, and *S*. *macrocarpa*, and *S. purpurea* resembles five deer antlers that are fused at the base and radiate from the center of the fruit (Fig. 26k, l and 28d). In this central structure, the five elliptical to polygonal locules alternate with five prominent lacunae and their orbicules. The external regions of the lacunae are not closed with sclerenchyma tissue in the manner of lacunae in other species of *Spondias*, so they are enclosed instead by spongy tissue; giving the characteristic antler-like pattern of the endocarp as seen in cross section. Within each lacuna, there is a ventral, longitudinal row of orbicules (ca. 1**–**2 mm in diameter) as mentioned above. The orbicules may be seen in successive transverse sections but the longitudinal rows of them are best documented in longitudinal sections (Figs. 27e–g and 28e, f).

In *S. purpurea*, the internal germination pores are located close to the apex of the ovoid endocarp and are only visible when the soft tissue is removed. The pores are cryptic, spinose in appearance, and are recognized by pair of hook-like projections of the endocarp over each locule (Fig. 28g, h). *Spondias mombin* has an endocarp surface that is more or less smooth to fibrous and prominently ridged (Fig. 27a, b). The soft tissue of the stone, inside the fleshy mesocarp, is composed of a loose fibrous to parenchymatous meshwork. Five to six large internal germination pores are visible at the apex of the endocarp Fig. 27b). Inside each pore is an recessed internal bilabiate valve (Fig. 27d). Transverse equatorial sections of this species show elliptical to nearly circular stones with unevenly developed locules and alternating large rounded lacunae (Fig. 27c). The series of orbicules within the lacunae are well seen in sagittal sections (Fig. 27e, f, g). The locules are straight.

The endocarp surface in *S. tuberosa* is mostly smooth with inconspicuous longitudinal ridges (Fig. 28i). The base of the stone has a large circular depression (Fig. 28k). Removal of the thin and fleshy mesocarp exposes the five germination pores at the apical end of the stone (Fig. 28j). One of the pores is usually larger than the other four. Short to long hook-like projections are present on the upper wall of the germination pores (Fig. 28i, j). The pores commonly are partially occluded with loose fibers. The endocarp is elliptical in transverse section with five unevenly developed locules that are elliptical to triangular in outline; the specimen that we sectioned enclosed only two large lacunae (Fig. 28l).

Among Old World species, *Spondias dulcis* and *S. pinnata* have prolate, obovoid, ellipsoid to oblong fruits (26a, b; Fig. 27j**–**l). The surface of the endocarp is more or less smooth with long, longitudinal to sinuous strands of fibers. Small, scattered depressions are also present on the surface of the endocarp. In *S. pinnata* a whorl of five small depressions is also present at the base of the endocarp (Fig. 27k). The fruits in both species are circular in transverse equatorial section (Fig. 26b). The endocarp is more or less star-shaped in transverse sections and is enclosed by a mesocarp composed of a loose matrix of fibers and soft tissue (Fig. 27l). The hard endocarp encloses up to five radially arranged locules that appear elliptical in transverse section (Fig. 26b). The conspicuously spinose sclerified portion of the endocarp is exposed when the spongy tissue is manually removed (Fig. 26c). In lateral view the endocarps appear obovate in outline. Spiny projections of variable length extend into the spongy tissue and radiate in all directions from the center of the fruit. Sagittal sections reveal short, more or less reniform locules, which are connected to internal germination pores (Fig. 26c).

Only one specimen of *Spondias novoguineensis* was studied in detail (Fig. 27h, i). When the fruit was boiled most of the spongy tissue was removable, exposing the strongly ribbed and spinose endocarp. Near the base, the endocarp projections are conspicuously spinose, and thin, with pronounced tips, while near the apex, the spines are truncate and stout. Five large recessed internal germination pores are present at the apex of the endocarp (Fig. 27i).

*Spondias bipinnata* has been indicated as distinct from other species based on its bipinnate rather than once-pinnate leaves, leaflets lacking an intramarginal vein, and its pubescent, connivent styles (Mitchell & Daly, 2015). Its fruits are larger than those of most other species and are oblong to obovoid with a rounded apex and base (Fig. 28m, o). The endocarp surface is highly reticulate. The reticulations are more pronounced at the base and apex of the endocarp while near the midsection they are less common. A spongy layer penetrates between the reticulations of the endocarp. The endocarp is circular in equatorial transverse section (Fig. 28r, t). Near the center of the endocarp are five radially arranged locules which are more or less elliptical in outline. Sagittal sections show short, straight locules (Fig. 28p, s). Cryptic pore-like structures can be observed by the interruption in the coarse surface reticulum with very fine hair-like fibrous processes, but the pores are mostly occluded by these dense loose fibers as seen in sagittal section (Fig. 28p).

In *Spondias* species the locular envelope is composed of horizontal encircling fibers forming a layer 100 to 300 μm thick. Between adjacent locular envelopes, the septum tissue is formed by isodiametric brachysclereids or parenchyma.

### *Tapirira* Aubl. (Fig. 29)

This is a Neotropical genus of eight or more species of trees or rarely shrubs distributed from southern Mexico to Bolivia, Paraguay and southeastern Brazil. The type species, *Tapirira guianensis*, was previously studied for pericarp and seed coat structure (Von Teichman, 1990). Four of the species sampled lack germination pores and valves. The species traditionally called *T*. *mexicana*, treated separately below, has a prominent germination pore and a recessed internal bilabiate dehiscence as well as rugose endocarps and therefore we do not believe that it belongs to this genus.

**Fig. 29:**
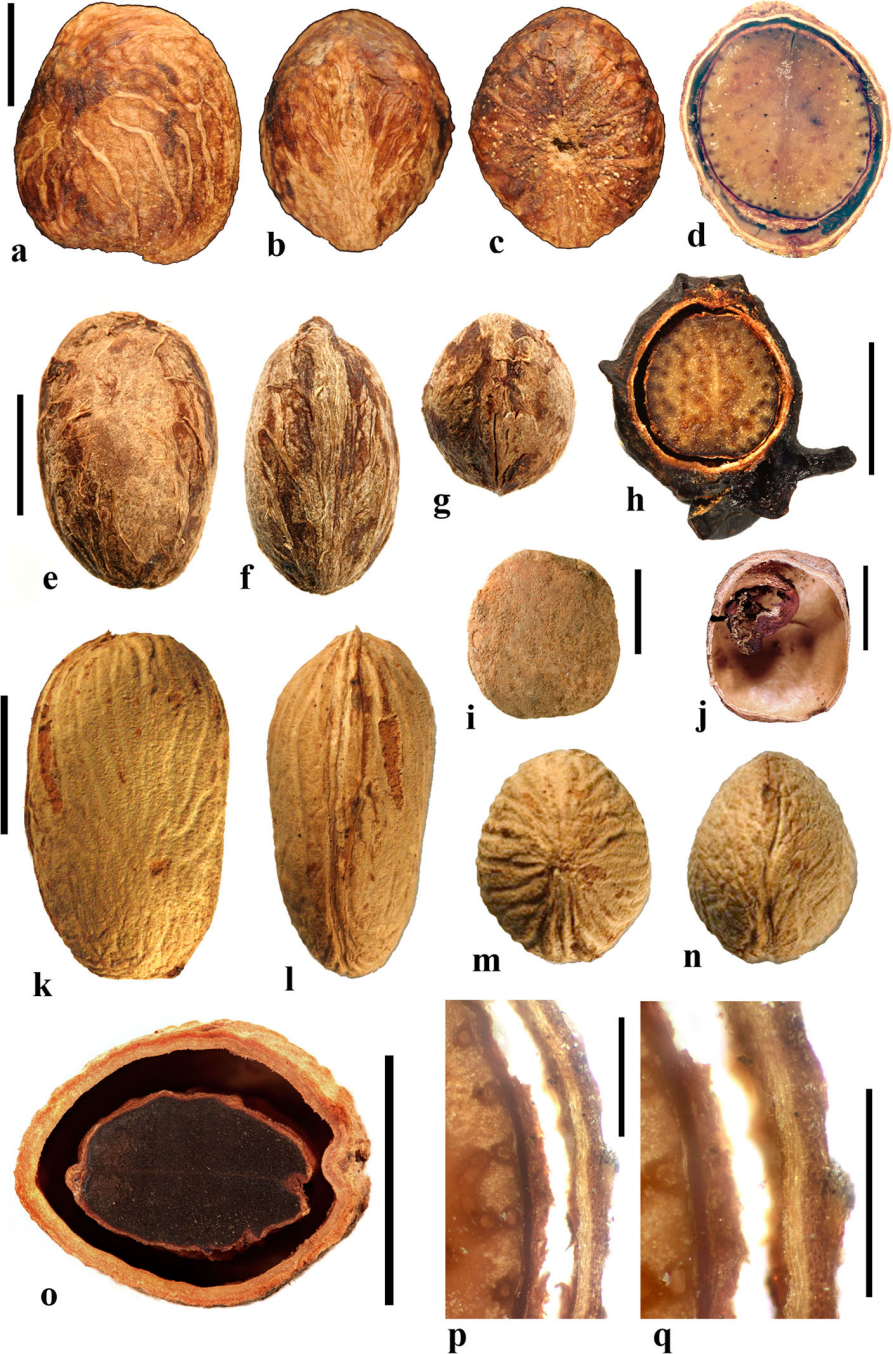
*Tapirira*. **a–d** *T. guianensis* stone in lateral, apical, and basal views and transverse section (FLAS 148032: M. Kimach, 175; Peru). **e–h** Lateral, frontal, apical views and transverse section of *T. guianensis* (P 5190727: Aubréville 388; R. Compte’; French Guiana). **i, j** Lateral view and sagittal longitudinal section of *T. guianensis* (UC 1442206: G. Hatschbach 41,738; Bairro Alto, Brazil). **k–o** *T. myriantha* stone in lateral, front, apical, and basal views, and transverse section (MO 2088147: Davidse & González 13,772; Venezuela). **p, q** *T. guianensis* transverse section. Scale bars 5 mm in a (also applies to b**–**d), e (also applies f, g), h**–**j; 1 cm in k (also applies to l**–**m), o; 1 mm in p, q

The mature fruit is black or purple. According to Von Teichman (1990) the mesocarp is thin and fleshy, and the outer part of the endocarp includes a network of vascular bundles and associated parenchyma. The endocarp surface is smooth, furrowed to reticulate (Fig. 29a**–**c, e**–**g, i, k**–**n). The endocarps are subglobose, ellipsoid, to ovoid, laterally compressed and bisymmetrical without an external keel (Fig. 29a**–**n). The stone is unilocular and relatively thin-walled in *T. guianensis* (Fig. 29d, h) but thicker-walled in *T. myriantha* (Fig. 29o). *Tapirira* stones are unusual among Spondioideae in the lack of specialized germination apparatus.

The locular envelope of *Tapirira guianensis* is 0.15 to 0.2 mm thick, and that of *T. myriantha* 0.4**–**0.5 mm thick, composed of horizontally encircling fibers (Fig. 29o**–**p). Outside the locular envelope is another layer of similar thickness, composed exclusively of isodiametric sclereids. A prominent whorl of secretory canals with purplish content occurs at the periphery of the cotyledons as seen in transverse sections of the seed in *T*. *guianensis* (Fig. 29d, h; Von Teichman, 1990), but this has not been observed in *T. myriantha* (Fig. 29p), nor in other genera of Spondioideae.

Von Teichman (1990, p. 438) commented on the possible adaptive significance of pericarp thickness: “*Tapirira* grows in a habitat in the tropical lowlands of South America which is generally warmer with greater moisture availability than in the savannas of southern Africa. This might possibly be one of the reasons why a massive protective endocarp has not developed in this species, but is present in the Spondioideae from South Africa.” (Von Teichman, 1990, p. 438).

### “*Tapirira*” *mexicana* Marchand (Fig. 30)

**Fig. 30:**
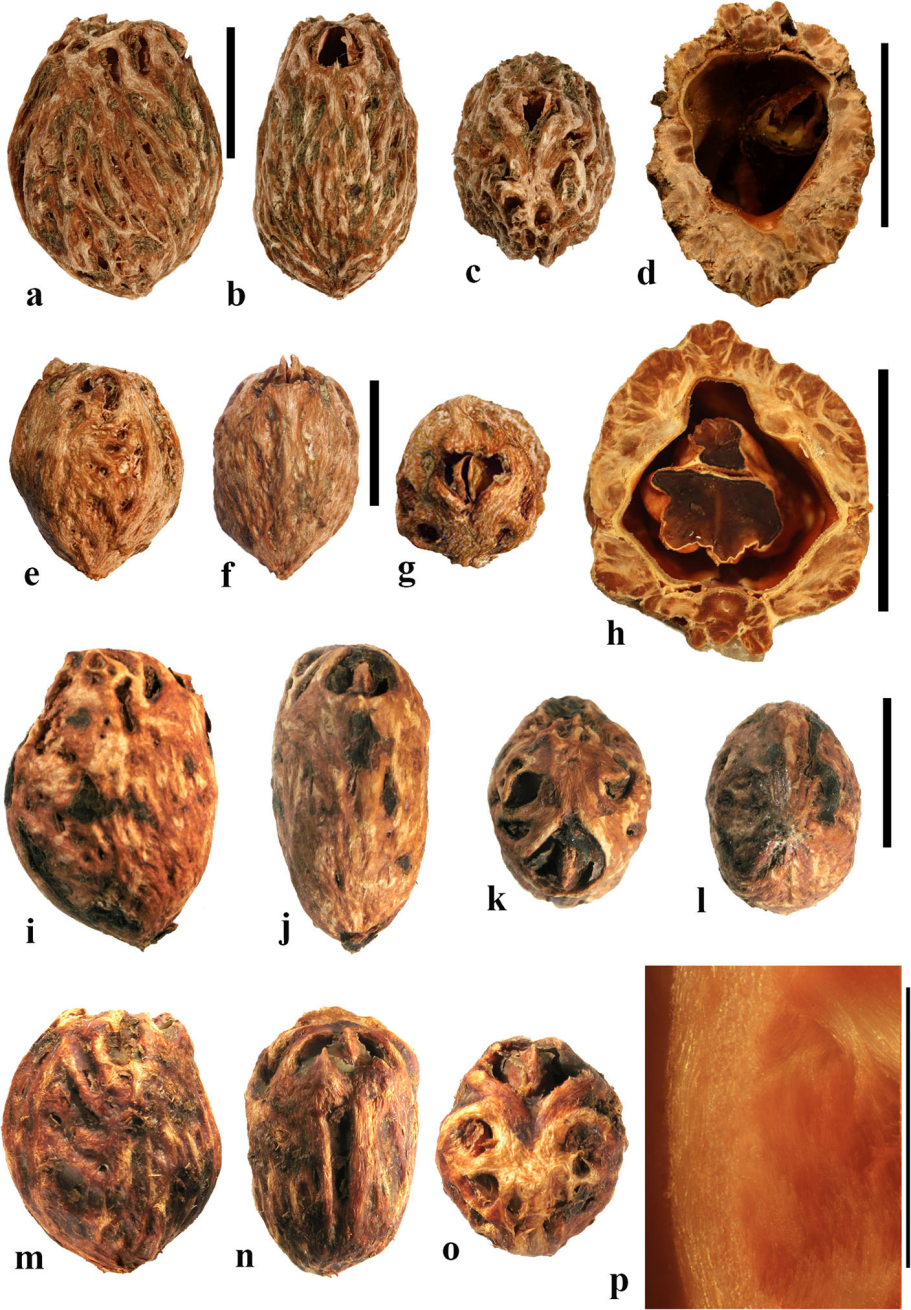
“*Tapirira*” *mexicana*. **a–d** Lateral, front, and apical views, and equatorial transverse section (MO 5045832: T Wendt et al. 5326; Altamirano, Chiapas, Mexico). **e–h** Lateral, front, and apical view and equatorial transverse section. (MO 4590657: W. Haber and W. Zuchowski 8726; Puntarenas, Costa Rica). Note “lips” of the recessed interal bilabiate germination. **i–l** Lateral, front, apical, and basal views (NY: B. Gutiérrez 3557; Veracruz, Mexico). **m–o** Lateral, front, and apical, and views (NY: Z. Fuentes 327; Puntarenas, Costa Rica). **p.** Detail of locular envelope in transverse section. Scale bars 1 cm in a (also applies to b, c), d, f (also applies to e, g), h, i (also applies to i**–**k, m**–**o); 1 mm in p

We studied several herbarium collections compatible with the type of *Tapirira mexicana* (Table 1). This species differs from the type species of the genus, and other species of *Tapirira,* by the presence of an apical pore with internal recessed bilabiate germination (Fig. 30a**–**c, e**–**g, i**–**o). The germination pore is elliptical in outline. The mature fruit is purple or yellow to orange, and the mesocarp is fleshy. The endocarp wall is thicker than in *T. guianensis* and *T. myriantha*. The stones are ellipsoid, oblong, to slightly ovoid (Fig. 30a, b, e, f, i, j). The endocarp surface is strongly rugose with abundant and prominent depressions at the apical end of the stone. These depressions do not appear to connect to the locule. Other minor depressions are also present on the lateral surfaces of the endocarp. The endocarp is unilocular and equatorial transverse sections show an irregularly shaped to more or less elliptical locule outline (Fig. 30d, h).

The locular envelope, ca. 0.18 mm thick, is composed of dense macrosclereids, rather than fibers (another difference from true *Tapirira*) grading outward into isodiametric parenchyma (Fig. 30d, h, p)*.* The remainder of the stone is formed by tortuous fibers in tracts arranged obliquely to anticlinally. In several respects, the endocarp of this species resembles that of *Cyrtocarpa caatingae.*

## Discussion

Detailed studies of fruit morphology across all genera of Spondioideae, as traditionally defined, plus the genera *Buchanania* and *Campnosperma* (suggested to be related on molecular grounds; Weeks et al. 2014), allows us to evaluate previous hypotheses of relationships and propose some new ones. At this stage, our understanding of relationships among these genera of Anacardiaceae is still in transition. Genera that have not been sampled for the molecular phylogeny include *Attilaea, Haematostaphis*, *Haplospondias*, *Koordersiodendron*, and *Solenocarpus*; and species sampling for DNA investigations remains limited for several of the component genera. Most of the currently recognized genera are readily distinguishable by the morphology of their endocarps (Table 2), and most, but not all, of these genera appear to be monophyletic based on uniformity of fruit morphology and anatomy among the species. *Tapirira mexicana* is clearly distinct from the type and other species of *Tapirira* by its apical endocarp pores with bilabiate germination.

*Cyrtocarpa* includes three different fruit types and is likely polyphyletic in its current circumscription. The typical endocarp morphology for this genus is smooth ellipsoidal with multiple locules and simple opercula that can be situated at different levels from apex to base of the fruit. However, *C. velutinifolia* has only one locule and a single prominent elliptical germination valve that is bisected by the fruit’s plane of bisymmetry. *C. caatingae* is also unilocular but differs further by the absence of a germination valve.

Based on fruit morphology, it seems likely that *Haplospondias* and *Solenocarpus* are sister taxa. Both genera have unilocular endocarps of similar size and morphology which are lenticular in cross section, with a pair of broadly elliptical germination valves at the apex, and a terete fibrous rib forming one of the lateral margins and continuing along the apical keel. We predict that molecular data, when they become available, will indicate a close relationship between these genera. Similar broadly elliptical germination valves occur also in *Lannea*, but usually in this genus there is only one germination valve*.*

We infer that two of the African genera, *Haematostaphis* and *Pseudospondias,* are closely related to each other. These are the only extant genera with bipartite, shutter-like opercula. Acquisition of sequence data for *Haematostaphis* would help to test this hypothesis. The distribution of this clade in Africa might be relictual, because the fossil genus *Pentoperculum* from the Eocene of North America and Europe, shares the same kind of bipartite operculum.

The phylogeny of Weeks et al. (2014), based on one nuclear and two chloroplast regions, indicates that some distinctive fruit morphological characters evolved multiple times. We failed to find morphological or anatomical fruit characters that would be helpful to distinguish the informally recognized clades, Spondioideae 1 and 2. Simple opercula evolved both in the *Spondias* complex, “Spondioideae 2” (*Dracontomelon*), and two or more times in the *Sclerocarya* complex, “Spondioideae 1” (*Cyrtocarpa*, *Sclerocarya*, *Antrocaryon*, and *Poupartia-Operculicarya*).

Within the *Sclerocarya* complex, *Tapirira* demonstrates a loss of specialized germination mechanism; it is depicted as sister to a clade in which the fruits have apical pores with recessed bilabiate germination (*Choerospondias* and *Pleiogynium*). A parallel loss may have occurred in the *Spondias* complex, where *Pegia*, which lacks a specialized germination mechanism, is sister to a clade with recessed bilabiate germination (*Allospondias* and *Spondias*). The fruit of *Pegia* is anomalous in comparison with all other Spondioideae because of the sticky substances incorporated within its pericarp, recalling *Anacardium* (Roth, 1977).

The strongly supported pairing of the Amazonian species of *Antrocaryon* with the Madagascar endemic, *Poupartiopsis* (Weeks et al., 2014)*,* is surprising given their greatly different endocarp morphologies, the former having hard oblate endocarps with well-defined opercula, and the latter having highly dissected spongy fibrous endocarps with recessed bilabiate germination. The phylogeny presented by Weeks et al. (2014) would indicate that similarities between fruits of *Poupartiopsis* and *Spondias* are due to homoplasy.

*Campnosperma,* which is sister to the large clade of Spondioideae 1 plus Anacardioideae (Weeks et al., 2014), does not conform to Spondioideae in its fruit morphology. It lacks a specialized germination apparatus and is unique in its strongly curved seed; the endocarp of *Campnosperma* also is anatomically unique, compared to all the Spondioideae and Anacardioideae that we have examined, being composed entirely of isodiametric sclereids, without any fibers, and lacks differentiation of a locular envelope.

Some of the main characters that can be potentially recognized in fossil endocarps and that are useful for the identification of genera include (Table 2), a) locule number, b) the type and shape of germination mechanism and its position along the endocarp (i.e, simple opercula, bipartite opercula, recessed bilabiate, external valves or flaps, and apical slit), c) number, shape and position of lacunae, d) ornamentation of the endocarp, and e) presence and position, or absence of apertures. The systematic and detailed survey of extant Spondioid fruits presented here should serve as a basis for the reexamination of the historic collections of Spondioid fossil endocarps (e.g., Reid & Chandler, 1933; Chesters, 1957) as well for the identification of new endocarp fossils (e.g., Collinson et al., 2012; Herrera, 2014). Fossil endocarps of *Antrocaryon*, *Choerospondias*, *Dracontomelon*, *Lannea*, *Pleiogynium*, and *Spondias* have been reported from Eocene to Pliocene deposits in Europe, Asia, Africa, Central America, and Australia (e.g., Chandler, 1961; Chesters, 1957; Collinson et al. 2012; Bonnefille & Letouzey, 1976; Herrera, 2014; Manchester et al., 2009; Reid & Chandler, 1933; Rozefelds et al., 2015; Tiffney et al., 1994). Although recent dated molecular studies (e.g., Weeks et al., 2014) suggest that Spondioideae should have evolved during the Middle-Late Cretaceous, pre-Eocene fruits conforming in morphology to Spondioideae still remain elusive.
